# Direct Synthesis of N‐formamides by Integrating Reductive Amination of Ketones and Aldehydes with CO_2_ Fixation in a Metal‐Organic Framework

**DOI:** 10.1002/chem.202303289

**Published:** 2023-12-12

**Authors:** Wenyuan Huang, Qingqing Mei, Shaojun Xu, Bing An, Meng He, Jiangnan Li, Yinlin Chen, Xue Han, Tian Luo, Lixia Guo, Joseph Hurd, Daniel Lee, Evan Tillotson, Sarah J. Haigh, Alex Walton, Sarah J. Day, Louise S. Natrajan, Martin Schröder, Sihai Yang

**Affiliations:** ^1^ Department of Chemistry University of Manchester Manchester M13 9PL UK; ^2^ Department of Chemical Engineering University of Manchester Manchester M13 9PL UK; ^3^ UK Catalysis Hub Research Complex at Harwell Rutherford Appleton Laboratory Harwell OX11 0FA UK; ^4^ College of Chemistry Beijing Normal University Beijing 100875 China; ^5^ Department of Materials University of Manchester Manchester M13 9PL UK; ^6^ Photon Science Institute University of Manchester Manchester M13 9PL UK; ^7^ Diamond Light Source Harwell Science Campus Oxfordshire OX11 0DE UK; ^8^ College of Chemistry and Molecular Engineering Beijing National Laboratory for Molecular Sciences Peking University Beijing 100871 China

**Keywords:** N-formylation, Reductive amination, MFM-300(Cr), SXPD, ssNMR

## Abstract

Formamides are important feedstocks for the manufacture of many fine chemicals. State‐of‐the‐art synthesis of formamides relies on the use of an excess amount of reagents, giving copious waste and thus poor atom‐economy. Here, we report the first example of direct synthesis of N‐formamides by coupling two challenging reactions, namely reductive amination of carbonyl compounds, particularly biomass‐derived aldehydes and ketones, and fixation of CO_2_ in the presence of H_2_ over a metal‐organic framework supported ruthenium catalyst, Ru/MFM‐300(Cr). Highly selective production of N‐formamides has been observed for a wide range of carbonyl compounds. Synchrotron X‐ray powder diffraction reveals the presence of strong host‐guest binding interactions via hydrogen bonding and parallel‐displaced π⋅⋅⋅π interactions between the catalyst and adsorbed substrates facilitating the activation of substrates and promoting selectivity to formamides. The use of multifunctional porous catalysts to integrate CO_2_ utilisation in the synthesis of formamide products will have a significant impact in the sustainable synthesis of feedstock chemicals.

## Graphical abstract

Ru/MFM‐300(Cr) was used as catalyst for the one‐pot N‐formylation of both aldehydes and ketones (30 substrates) in high yield. Synchrotron PXRD reveals the activation of carbonyl compounds via confined binding within MFM‐300(Cr). Nuclear magnetic resonance (NMR) spectroscopic analysis and control experiments suggest formate species as the intermediate derived from the reduction of CO_2_ and reveal the possible reaction pathway.

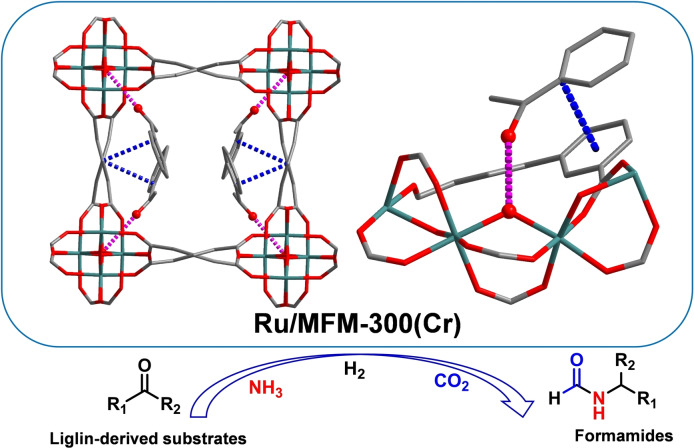

## Introduction

**Figure 1:**
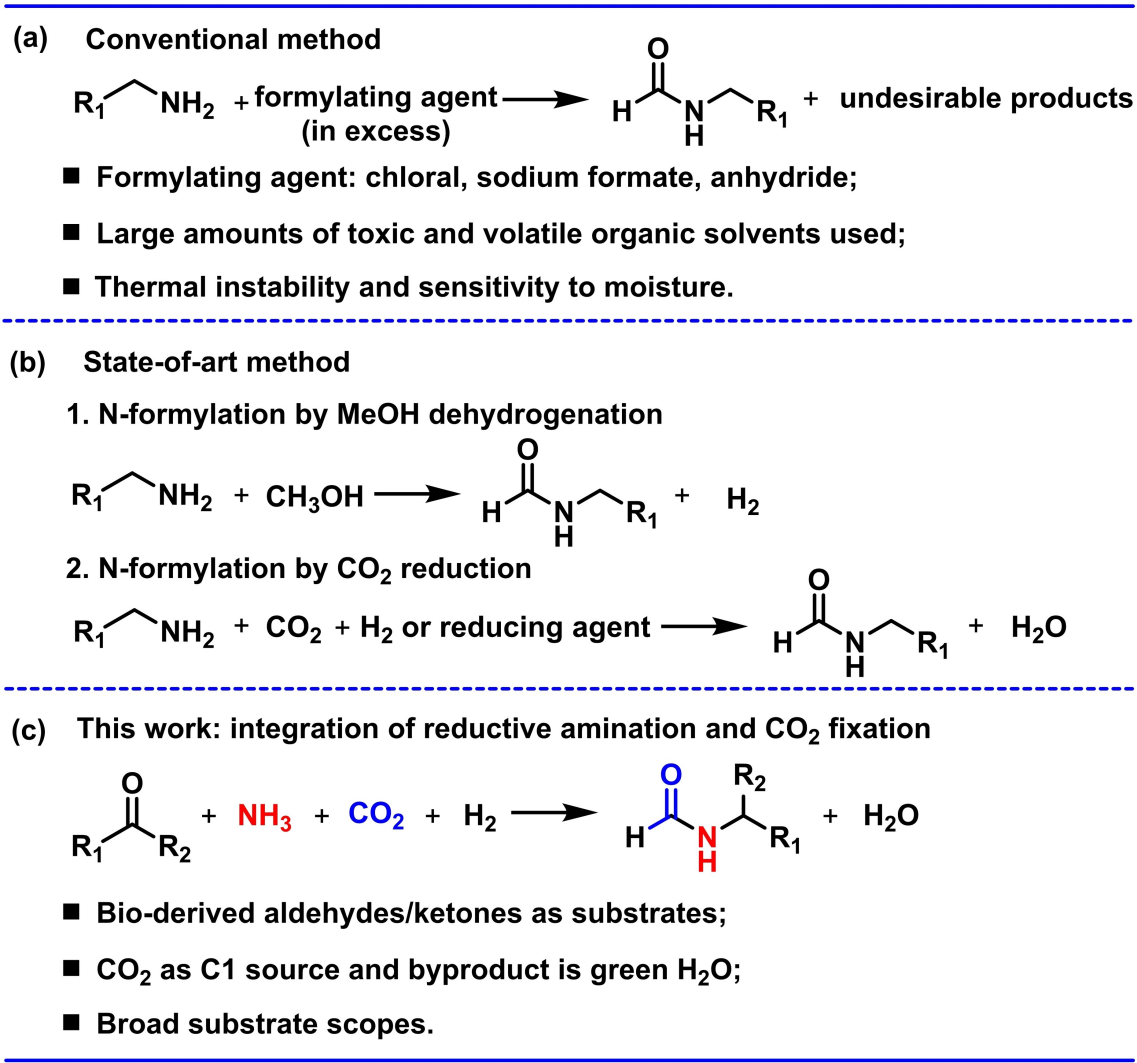
**Routes to the preparation of N‐formamides**. a. Conventional methods by reaction of excess formylating agents (chloral, sodium formate, formaldehyde, acetic formic anhydride and/or formic acid) with organic amines.^[7]^ b. State‐of‐the‐art methods involving MeOH derived from CO_2_ ^[31]^ and the direct use of CO_2_ ^[19]^ as the C1 source. c. One‐pot approach integrating the reductive amination of carbonyl compounds with CO_2_ fixation.

Formamides are important platform chemicals for the production of pharmaceuticals, fragrances, agrochemicals, dyes and industrial solvents.[^1^, ^2^, ^3^, ^4^] State‐of‐the‐art synthesis of formamides relies on the utilisation of an excess amount of the formylating agents [e. g., chloral (trichloroacetaldehyde), sodium formate, formaldehyde, acetic formic anhydride and formic acid] that react with organic amines to drive the formation of C−N bonds, yielding substantial amounts of waste and thus extremely poor atom‐economy (Figure 1a).[^2^, ^5^, ^6^, ^7^] In the context of development of sustainable chemistry, the utilisation of carbon dioxide (CO_2_)[^8^, ^9^, ^10^] and lignocellulosic biomass and their derivatives[^11^, ^12^, ^13^] for the manufacture of feedstock chemicals has attracted much interest. Although methanol derived from CO_2_ has been applied for the preparation of formamides,[^1^, ^14^, ^15^] the formylation of amines via the direct use of CO_2_ is a more promising but challenging target (Figure 1b).[^16^, ^17^, ^18^, ^19^] This can be achieved by reducing CO_2_ with H_2_ over noble metals, followed by condensation with an amine to form a new C−N bonded formamide. In parallel, a wide range of ketones and aldehydes can be readily obtained from biomass,^[20]^ but their conversion to organic amines via reductive amination is also a highly challenging process.[^21^, ^22^, ^23^, ^24^]

Here, we report the first example of direct synthesis of N‐formamides from carbonyl compounds, including various ketones and aldehydes derived from lignin (Figure S1), by reaction of CO_2_, NH_3_ and H_2_ over a metal‐organic framework (MOF) supporting a ruthenium catalyst, Ru/MFM‐300(Cr) (Figure 1c). This one‐pot approach effectively integrates two challenging reactions, reductive amination of carbonyl compounds and CO_2_ fixation, by developing a multifunctional porous reactor. MFM‐300(Cr) forms a support to achieve fine dispersion of Ru nanoparticles (0.54±0.29 nm), and also acts as gas reservoir for CO_2_ and NH_3_. More importantly, it also provides a unique platform to activate carbonyl substrates via confinement effects induced by the formation of specific host‐guest interactions within the pore. This has been analysed by in situ synchrotron X‐ray powder diffraction (SXPD) and Fourier‐transform infrared spectroscopy (FTIR), while nuclear magnetic resonance (NMR) spectroscopic analysis and control experiments suggest formate species as the intermediate derived from the reduction of CO_2_. This one‐pot system affords excellent catalytic performance as well as stability for the synthesis of a wide range of N‐formamides, which can be isolated readily from the reaction.

## Results and Discussion

MFM‐300(Cr),^[25]^ [Cr_2_(OH)_2_(L)] (H_4_L=biphenyl‐3,3’,5,5’‐tetracarboxylic acid), has been selected in this study due to its high stability in alkaline solution (Figure S2) and high adsorption uptakes of CO_2_ and NH_3_ (Figures S3 and S4). MFM‐300(Cr) was prepared by hydrothermal reaction of H_4_L and CrCl_3_ ⋅ 6H_2_O in water containing HCl at 210 °C. Power X‐ray diffraction (PXRD) analysis confirmed the purity of the bulk material (Supplementary Figure S5). Ruthenium has been widely demonstrated to show excellent activity for the reductive amination and N‐formylation reactions,[^19^, ^20^, ^21^, ^26^, ^27^] and is employed here to promote the one‐pot N‐formylation of carbonyl compounds. The Ru/MFM‐300(Cr) catalyst was prepared via incipient wetness impregnation of desolvated MFM‐300(Cr) using an aqueous solution of RuCl_3_, followed by reduction in 5 % H_2_/Ar at 250 °C for 6 h (see Experimental Section). Various other catalysts based upon MFM‐300(Al) and conventional metal oxides (Cr_2_O_3_, Al_2_O_3_, ZrO_2_) and noble metals of Pd, Rh have also been synthesised for comparison (Table 1).

**Table 1:** N‐formylation of benzaldehyde (**1a**) to N‐benzylformamide (**2a**) using various catalysts.

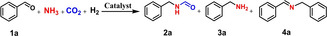				
Entry^[a]^	Catalyst	Yield (%)		
		**2a**	**3a**	**4a**
1	null	–	–	52
2	MFM‐300(Cr)	–	Trace	68
3	Ru/MFM‐300(Cr)	90	5	2
4	Ru/MFM‐300(Al)	82	17	Trace
5	Ru/ZrO_2_	68	19	8
6	Ru/Cr_2_O_3_	63	16	17
7	Ru/Al_2_O_3_	72	11	9
8	Rh/MFM‐300(Cr)	32	64	2
9	Pd/MFM‐300(Cr)	18	47	24
10	Ru nanoparticles	32	18	37
11	Ru nanoparticles+MFM‐300(Cr)	60	12	21
12^[b]^	Ru/MFM‐300(Cr)	Trance	65	18

[a] Reaction conditions: 7 mol/L NH_3_/MeOH solution (2 mL), benzaldehyde (1 mmol), 16 h, 433 K, catalyst (10 mg), H_2_ (4 MPa), CO_2_ (3 MPa). Yields were determined by GC using dodecane as the internal standard. [b] No CO_2_ was added.

**Figure 2:**
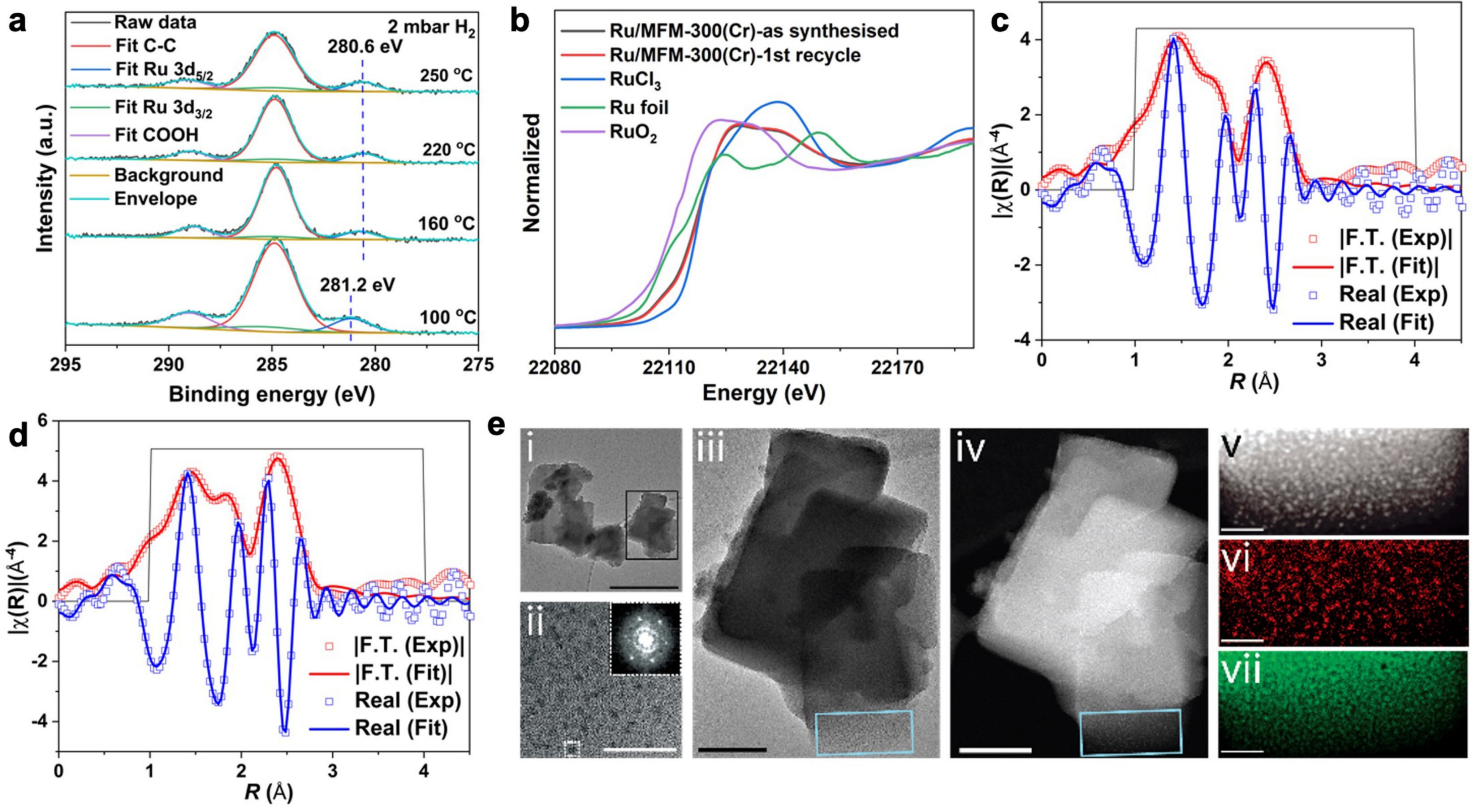
**Characterisation of the catalysts**. a: In situ Ru XPS spectra of Ru/MFM‐300(Cr) (having been exposed to air for one week) under exposure of H_2_ at 100, 160, 220 and 250 °C for 30 mins. b: Ru K‐edge XANES profiles of Ru/MFM‐300(Cr)‐as synthesised, Ru/MFM‐300(Cr)‐after 1st cycle, RuCl_3_, RuO_2_, and Ru foil. c: Experimental EXAFS spectra and fitting for Ru/MFM‐300(Cr)‐as synthesized in R space showing the magnitude of Fourier transform (red hollow squares, red line) and the real component (hollow squares, blue line). The fitting range is 1.0–4.0 Å in R space (within the black line). d: Experimental EXAFS spectra and fitting of Ru/MFM‐300(Cr)‐used in R space showing the magnitude of Fourier transform (red hollow squares, red line) and real components (hollow squares, blue line). The fitting range is 1.0–4.0 Å in R space (within the black line). e: Transmission electron microscopy (TEM) and scanning transmission electron microscopy (STEM) of Ru/MFM‐300(Cr). TEM images at different scale bars (i‐iii), high‐angle annular dark field (HAADF) STEM images (iv‐v), and EDS mapping of Ru (vi) and Cr (vii). Inset in ii): A fast Fourier transform (FFT) shows periodicities of 2.2 Å, consistent with the <1010> directions expected for Ru nanoparticles with the space group, P63/mmc. The scale bars are 500 nm for (i), 100 nm for (iii and iv), and 25 nm for (ii) and (v‐vii).

The absence of structural change of MFM‐300(Cr) upon loading of Ru and the absence of bulk Ru particles have been confirmed by PXRD analysis (Figure S5). Elemental analysis of Ru/MFM‐300(Cr) suggests a molar ratio of Cr/Ru of 5.2 (equivalent to 3 wt %), consistent with that obtained by thermogravimetric analysis (5.2) (Table S1 and Figure S6). N_2_ adsorption isotherms at 77 K confirm that the introduction of Ru leads to a decrease of the Brunauer‐Emmett‐Teller (BET) surface area from 1146 m^2^ g^−1^ for bare MFM‐300(Cr) to 832 m^2^ g^−1^ for Ru/MFM‐300(Cr) (Figure S7), consistent with the pores being partially occupied by Ru sites. In situ diffuse reflectance infrared Fourier transform spectroscopy (DRIFTs) of chemisorbed CO on Ru/MFM‐300(Cr) shows three υCO bands at 2134, 2074 and 2012 cm^−1^ (Figure S8), which can be assigned to multi‐carbonyl [Ru^n+^(CO)_x_], mono‐carbonyl (Ru^n+^‐CO) species adsorbed on partially oxidised Ru^n+^ sites and linearly adsorbed CO molecules on metallic Ru (Ru_x_‐CO), respectively.^[28]^ In situ X‐ray photoelectron spectroscopy (XPS, Figure 2a and Figure S9) and temperature‐programmed reduction (TPR, Figure S10) demonstrate that the oxidised ruthenium sites can be reduced at the reaction temperature, suggesting that surface oxygen on Ru has little influence on the catalytic performance under the reaction conditions.

To determine the electronic and local structures of Ru species, X‐ray absorption spectroscopy (XAS) at the Ru K‐edge, including the X‐ray absorption near edge structure (XANES) and the extended X‐ray absorption fine structure (EXAFS) analyses have been employed. Compared with the peak positions of Ru(0) in Ru foil and Ru(III) in RuCl_3_, the XANES spectrum of Ru/MFM‐300(Cr) shows features of both Ru(0) and Ru(III) (Figure 2b), indicating the presence of mixed valence states and consistent with the CO‐DRIFTs and XPS results. Fittings of the Fourier transformed (FT) k^2^‐weighted EXAFS regions afforded a coordination number of 2.36±0.56 for Ru−Ru, lower than that (12 coordination) of Ru in bulk metal. Such a low coordination number corresponds to an average Ru particle size of 0.54±0.29 nm, which is in excellent agreement with the pore size (ca. 0.7 nm) of MFM‐300(Cr) (Figures 2c, 2d, Figure S7, Table S2). It is worth noting that the presence of minor amounts of Ru−Cl species (coordination number=0.60±0.06) and Ru−O bonds (coordination number=5.90±1.26) result from incomplete reduction of the samples and will lower the average coordination number for Ru sites thus underestimating the size of Ru nanoparticles of Ru/MFM‐300(Cr). Transmission electron microscopy (TEM) images confirm the homogeneous distribution of nanosized Ru nanoparticles with the size of 0.42±0.12 nm throughout MFM‐300(Cr) (Figure 2e, Figures S11 and S12), which is consistent with the XANES analysis.

These catalysts were tested for the N‐formylation of benzaldehyde (**1a**) to N‐benzylformamide (**2a**) in a solution of NH_3_ in MeOH in the presence of CO_2_ (3 MPa) and H_2_ (4 MPa) at 160 °C for 16 h in batch mode as a model reaction (Table 1). MFM‐300(Cr) shows no catalysis to the target product. Importantly, 3 wt % Ru/MFM‐300(Cr) shows an excellent yield of 90 % for **2a** on the full conversion of **1a**, and is higher than the yields observed for 1 wt % and 5 wt % Ru‐loaded catalysts (Figure S13). In contrast, Rh/MFM‐300(Cr) and Pd/MFM‐300(Cr) show much lower yields of **2a** (32 % and 18 %, respectively) with benzylamine as the main product (yield of 64 % and 47 %, respectively), indicating a poor activity for the reduction of CO_2_. Interestingly, Ru/MFM‐300(Al) also shows nearly full conversion of **1a**, but a lower yield of **2a** (82 %), suggesting that the acidity of the [M−O(H)−M] (M = Cr, Al) moieties can impact the activation of substrates. Ruthenium nanoparticles supported on metal oxides, such as Ru/ZrO_2_, Ru/Al_2_O_3_ and Ru/Cr_2_O_3_, and commercial bulk Ru nanoparticles all give lower yields to N‐benzylformamide (68 %, 63 %, 72 % and 32 %, respectively). Interestingly, a powdered mixture of Ru nanoparticles and MFM‐300(Cr) shows a much higher yield of **2a** (60 %) than bulk Ru nanoparticles, indicating the key role of Ru/MFM‐300(Cr) in the activation of substrates likely via adsorption‐induced host‐guest interactions and the acidity of the MOF (Figure S14). Overall, these results confirm that the direct cooperation of ruthenium nanoparticles and MFM‐300(Cr) plays a vital role in the one‐pot N‐formylation reaction.

The scope of N‐formylation of various carbonyl compounds over Ru/MFM‐300(Cr) has been investigated (Table 2). A wide range of carbonyl compounds can be converted to the corresponding N‐formamides in high isolated yields of up to 98 %. Compared with benzaldehyde (Table 2, compound **1**), the conversion of 4‐methoxy‐ and 4‐methyl‐substituted aldehydes with electron‐donating groups shows similar yields of the corresponding formamides (84–90 %, Table 2, compound **2** and **11**). However, the conversion of 2‐methyl‐substituted aldehyde to N‐(2‐methylbenzyl)formamide gives a lower yield of 53 % (Table 2, compound **4**), indicating that the o‐methyl group hinders the adsorption of C=O group in compound **4** in the pore. The conversion of electron‐withdrawing 4‐fluoro‐ and 4‐chloro‐substituted benzaldehydes shows high yields of N‐formamides (98 % and 90 %, respectively; Table 2, compound **7** and 8). Furthermore, a wide range of aromatic ketones with higher reductive potentials than aldehydes, such as acetophenone (Table 2, compound **21**) and 4‐methoxyacetophenone (Table 2, compound **26**), can also be selectively transformed into the corresponding N‐formamides with high yields (80 % and 91 %, respectively). Importantly, the conversion of biomass‐derived furfural and 5‐methyl furfural (Table 2, compound **19** and **20**) shows high yields (83–86 %) of the corresponding formamides. More interestingly, aliphatic aldehydes and ketones can also be converted efficiently in this system to the corresponding formamides (Table 2, compound **16**–**18** and **30**). Overall, Ru/MFM‐300(Cr) demonstrates a high tolerance to functional groups of the substrates in this one‐pot process.

**Table 2:**
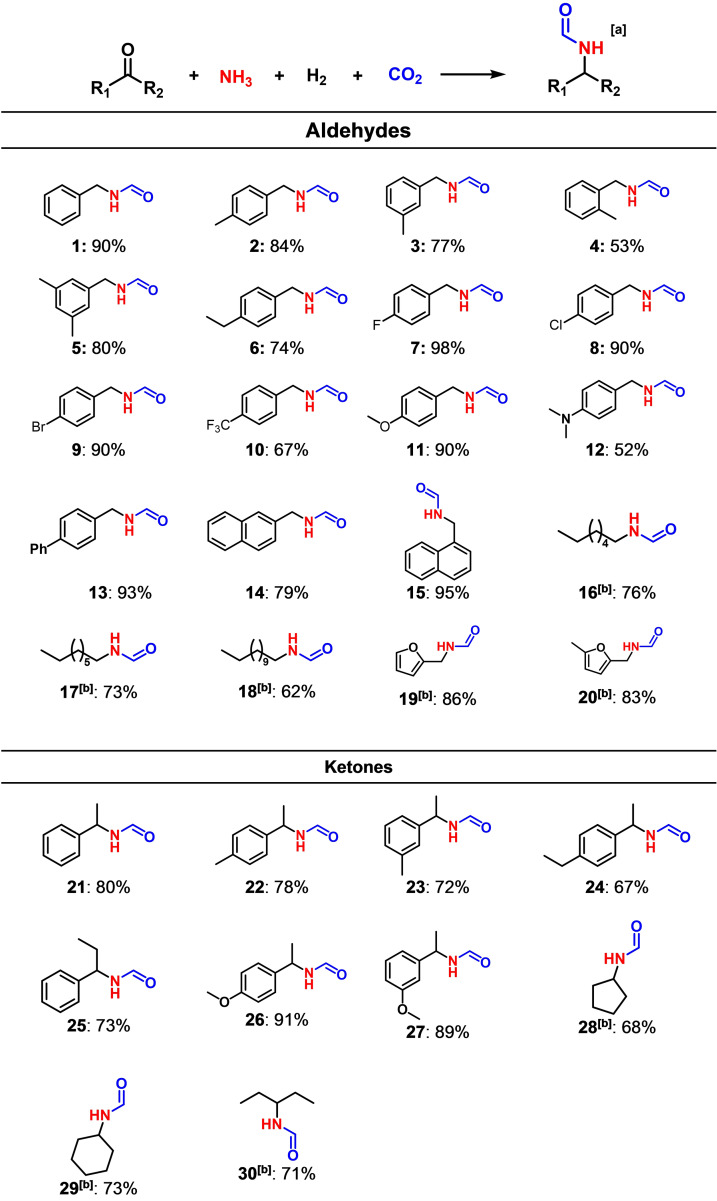
Yields from N‐formylation reactions

[a] Isolated yields as given. Reaction conditions: 7 mol/L NH_3_/MeOH solution (2 mL), substrates (1 mmol), 16 h, 433 K, catalyst (10 mg), H_2_ (4 MPa), CO_2_ (3 MPa). [b] Reaction conditions: 7 mol/L NH_3_/MeOH solution (2 mL), substrates (0.5 mmol), 24 h, 433 K, catalyst (10 mg), H_2_ (3 MPa), CO_2_ (1 MPa). Yields were determined by NMR spectroscopy using 1,3,5‐trimethoxybenzene as the internal standard.

**Figure 3:**
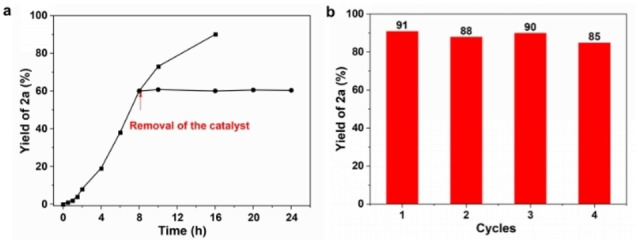
**Leaching test and catalyst reuse experiment**. a: the effect of removing Ru/MFM‐300(Cr) catalyst from the N‐formylation reaction of benzaldehyde. b: Reaction profiles for the cycling experiments using Ru/MFM‐300(Cr). Yields were determined by GC analysis.

The heterogeneous nature of the catalytic system has been confirmed by leaching experiments using **1a** as the model compound. The Ru/MFM‐300(Cr) catalyst was removed by centrifugation when the yield of **2a** reached 60 %, and little further production of **2a** was observed from the reaction system without Ru/MFM‐300(Cr) (Figure 3a). The concentrations of Ru and Cr in the filtrate were found to be negligible (below the detection limit) using inductively coupled plasma atomic emission spectroscopy (ICP‐AES), confirming the heterogeneous nature of the catalysis reaction. The catalyst can be readily retrieved from the reaction mixture by simple centrifugation with a recovery rate of 92 %. Furthermore, the recovered catalyst can be reused for at least four cycles, showing slightly reduced yields of **2a** (Figure 3b). The PXRD pattern of the fresh and used catalyst after the fourth reaction confirms the full retention of the crystal structure of MFM‐300(Cr) (Figure S5).

High resolution SXPD data has been collected for Ru/MFM‐300(Cr) loaded with benzaldehyde (**2a**), acetophenone (**3a**), 4‐methoxybenzaldehyde (**2k**), and biphenyl‐4‐carboxaldehyde (**2m**). Full structural analyses of the SXPD data have yielded highly satisfactory indicators of Rietveld refinements (Figure S15, Table S3). The guest‐loaded Ru/MFM‐300(Cr) materials display full retention of the framework structure (Figure S16). Hydrogen bonding between the bridging −OH groups of MFM‐300(Cr) and the carbonyl groups of substrates [Cr‐OH⋅⋅⋅O=C=2.91(3), 2.93(7), 2.81(5) and 2.42(2) Å in complexes of **2a**, **3a**, **2k**, and **2m**, respectively] are the primary interaction in all host‐guest structures. These adsorbed molecules are further stabilised by parallel‐displaced π⋅⋅⋅π interactions between the benzene rings of guest molecules and ligands of MFM‐300(Cr) with an inter‐planar distance of 3.57(3), 3.29(1), 3.31(1), and 3.42(6) Å for complexes of **2a**, **3a**, **2k**, and **2m**, respectively. Thus, the structural analyses confirm strong host‐guest interactions between Ru/MFM‐300(Cr) and the confined substrate molecules, enabling efficient pre‐activation of the substrates. The binding dynamics has been further studied by in situ FTIR, which shows a red shift of the νCO band upon adsorption of substrates (Figure S17), confirming a specific interaction between the carbonyl group and the host.^[29]^ Thus, the remarkable selectivity for N‐formamide formation by Ru/MFM‐300(Cr) originates from the strong adsorption and binding of substrate molecules.

**Figure 4:**
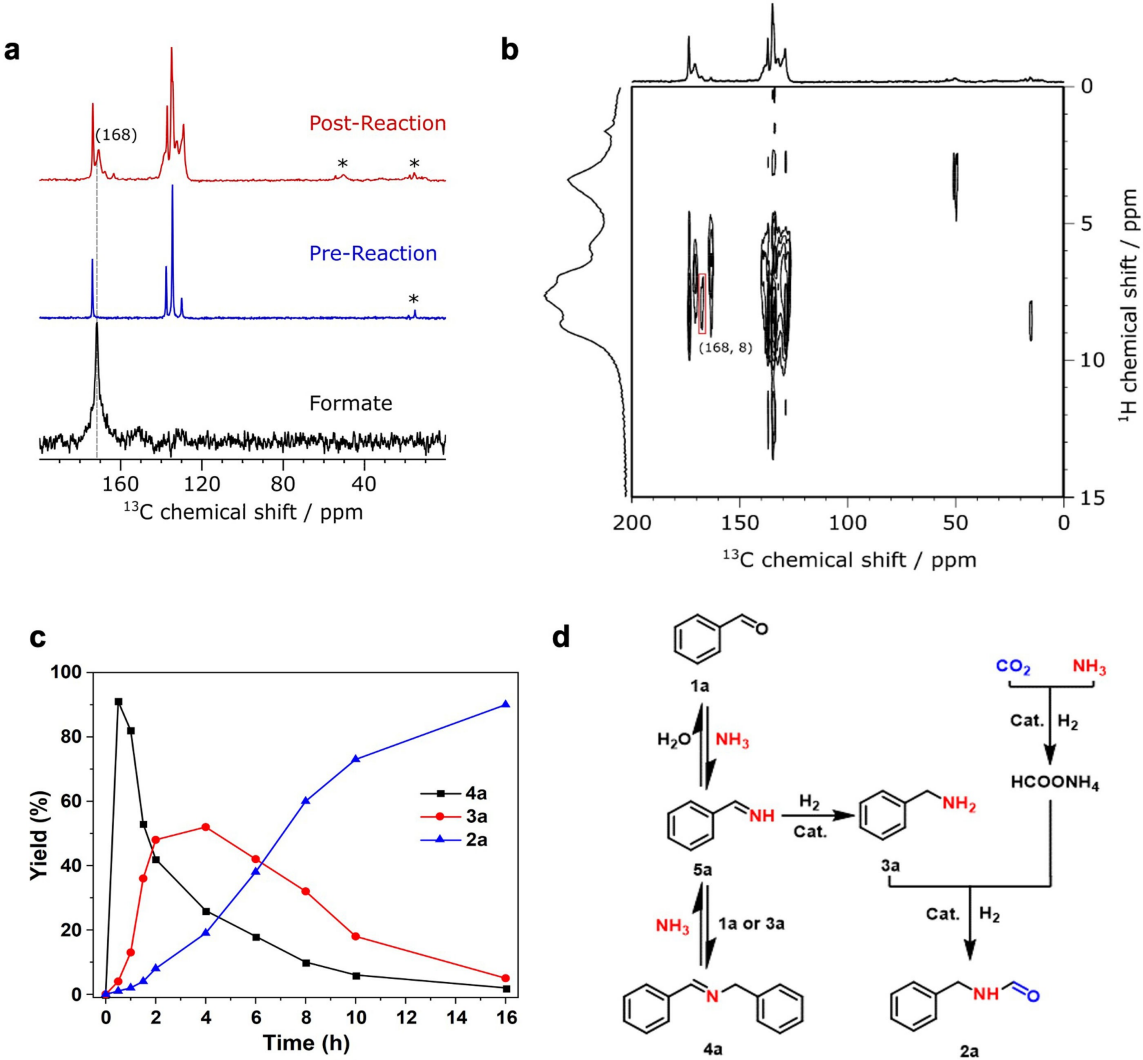
**NMR spectroscopic and reaction profile analysis**. Top: overall reaction scheme. a: ^13^C CP‐MAS ssNMR spectra before and after reaction (Ru/MFM‐300(Al)+NH_3_/MeOH+CO_2_+H_2_ for 3 h) showing the presence of formate species. * denotes spinning side band. Reaction condition: Ru/MFM‐300(Al) (10 mg), NH_3_/MeOH (7 mol/L, 2 mL), CO_2_ (3 MPa), H_2_ (4 MPa), 433 K. b: 2D ^1^H‐^13^C FSLG‐HETCOR NMR spectrum after reaction (Ru/MFM‐300(Al)+NH_3_/MeOH+CO_2_+H_2_ for 3 h) showing the presence of formate species. Reaction condition: Ru/MFM‐300(Al) (10 mg), NH_3_/MeOH (7 mol/L, 2 mL), CO_2_ (3 MPa), H_2_ (4 MPa), 433 K. c: Time course for N‐formylation of **1a** over Ru/MFM‐300(Cr) catalyst. Reaction conditions: catalyst (10 mg), **1a** (1 mmol), NH_3_/MeOH (7 mol/L, 2 mL), CO_2_ (3 MPa), and H_2_ (4 MPa), 433 K. d: Proposed reaction pathway. The transformation of benzaldehyde (**1a**) to the target product N‐benzylformamide (**2a**).

To investigate the intermediates produced from hydrogenation of CO_2_, the reaction of H_2_/CO_2_ in NH_3_/MeOH was conducted under reaction conditions and monitored by ^1^H and ^13^C NMR solution and solid‐state NMR (ssNMR) spectroscopy. The ^1^H and ^13^C solution NMR spectra showed the appearance of strong signals at 8.42 and 167.9 ppm, respectively, indicating the formation of formate species in the catalytic process (Figures S18 and S19).^[30]^ The two‐dimensional (2D) heteronuclear single quantum coherence (HSQC) NMR spectrum confirmed the correlation of the two signals (Figure S20). The Cr(III) centre is paramagnetic and causes fast relaxation and shifts of the NMR signals, so MFM‐300(Al)^[31]^ was used for the solid‐state NMR spectroscopic study. Solid‐state {^1^H‐}^13^C cross polarisation magic angle spinning (CP‐MAS) NMR analysis of Ru/MFM‐300(Al) after reaction showed a new peak at around 168 ppm. Furthermore, a 2D ^1^H‐^13^C heteronuclear dipolar correlation spectrum further confirms the presence of formate species on the used catalyst via a cross peak at δ{^1^H}~8 ppm / δ{^13^C}~168 ppm (Figure 4a and 4b). These results demonstrate that formic acid is formed by the hydrogenation of CO_2_ and stabilised as formate species in this system.

We sought to determine the reaction pathway based upon analysis of literature precedents[^10^, ^12^, ^14^, ^32^] and from control experiments. Figure 4c shows the time course of formation of N‐benzylformamide (**2a**) over Ru/MFM‐300(Cr). The yield of **4a** reached its maximum at 1 h, and on extending the reaction time the yield of **2a** and **3a** increases as the yield of **4a** decreases. **2a** then progressively becomes the main product with a decrease in the overall yields of **3a** and **4a**. The reaction pathway for the Ru/MFM‐300(Cr)‐catalysed synthesis of N‐formamide was evaluated by using **1a**, **3a** and **4a** as starting material (Figure S21). **1a** and **4a** can be transformed to **3a** in high yields in the absence of CO_2_. **2a** can be produced from **3a** and ammonium formate in a 98 % yield at 433 K, and a 72 % yield of **2a** can be obtained from **1a** and ammonium formate.

The mechanism of the conversion of benzyladhyde (**1a**) to N‐benzylformamide (**2a**) over Ru/MFM‐300(Cr) has been established (Figure 4d). First, the reversible reaction of **1a** with excess NH_3_ results in the formation of unstable N‐benzylideneamine (**5a**),^[21]^ which is hydrogenated to give benzylamine (**3a**). N‐Benzylidenebenzylamine (**4a**) is progressively produced by the subsequent reversible reaction of **3a** and **5a**. The intermediate **4a** is not further hydrogenated but is selectively transformed into **5a**, and further hydrogenated to **3a** under reaction conditions. Meanwhile, the hydrogenation of CO_2_ leads to the formation of formic acid that is stabilised by NH_3_ as ammonium formate, and **2a** is eventually produced by N‐formylation of **3a** with formic acid or ammonium formate.

## Conclusions

A new one‐pot approach has been developed to promote the synthesis of N‐formamides using biomass‐derived carbonyl compounds with CO_2_, H_2_ and NH_3_/MeOH over the multifunctional Ru/MFM‐300(Cr) catalyst. The reaction is suitable for a broad scope of substrates and the catalyst can be reused at least four times. In situ SXPD and FTIR studies confirm that carbonyl compounds can be preferentially adsorbed within the pores of MFM‐300(Cr), promoting the activation of substrates and improving the selectivity of target products by spatial confinement. Mechanistic studies suggest that ammonium formate is a key intermediate formed from CO_2_ and H_2_ under the reaction conditions, and reacts with the amine product to produce the target N‐formamides. The development of new porous microreactors, as demonstrated by Ru/MFM‐300(Cr), shows great potential in integrating multiple reactions within a one‐pot approach that minimises waste and efforts to separate reaction intermediates, thus driving the future sustainable production of fine chemicals.

## Experimental Section

**Materials**: All the chemicals and reagents used in this study were purchased from Fischer Scientific or Sigma Aldrich and used as received without further purification.

**Preparation of catalyst**: MFM‐300(Cr) was synthesised according to the literature method.^[25]^ H_4_BPTC (70 mg, 0.21 mmol), CrCl_3_ ⋅ 6H_2_O (200 mg, 0.75 mmol), deionised water (DI water, 10 mL) and 1 % HCl (1.5 mL) were mixed and transferred into a Teflon‐lined stainless‐steel autoclave, which was sealed and heated at 210 °C for 3 days. After cooling to room temperature, the resultant blue solid product was collected by centrifugation, washed with water and hot DMF several times, and dried at 60 °C under vacuum for 12 h. To remove DMF from within the pores, the as‐synthesized powder was processed by exchanging the guest solvent using Soxhlet extractor with acetone, followed by drying at 60 °C overnight.

MFM‐300(Al) was synthesized according to the literature method.^[31]^ H_4_BPTC (240 mg, 0.72 mmol), AlCl_3_ (480 mg, 3.6 mmol), were dissolved in a solution of DMF (60 mL) and 12 % HCl (12 mL) and heated in a Teflon‐lined stainless‐steel autoclave at 145 °C for 3 days. The procedure for post‐synthesis processing is the same as that of MFM‐300(Cr).

Ru/MFM‐300(Cr) was prepared by an incipient wetness impregnation‐reduction method. In a typical procedure, a solution containing the desired amount of RuCl_3_ dissolved in the minimum amount of water required to fully wet the sample of MFM‐300(Cr) was added dropwise to MFM‐300(Cr), stirred and left to evaporate. The sample was then reduced under 5 % H_2_/Ar flow (50 ml min^−1^) at 250 °C for 6 h. All the metal‐loaded samples were prepared by the same method except for the addition of different support material and/or the metal precursors.

### Characterisation

**PXRD measurement**. Powder X‐ray diffraction (PXRD) patterns were obtained using a Phillips X'pert Modular Powder Diffractometer fitted with a copper K‐α1 source (λ=1.5406 Å). The PXRD patterns were measured between 2θ values of 4° and 55° with a step size of 0.0167° and 34.9 s per degree.

**N_2_ sorption isotherms measurement**. N_2_ sorption isotherms were measured on a Micromeritics 3‐Flex gas sorption analyser at 77 K. Samples were acetone‐exchanged for 24 h and activated at 170 °C for 12 h under dynamic vacuum prior to the measurements. Thermal gravimetric analysis (TGA) curves were recorded using a TA Instruments SDT650 simultaneous thermal analyser. The sample was subjected to a heating regime of 5 °C min^‐1^ up to 800 °C under a flow of air to ensure the complete combustion of organic components.

**Elemental analysis**. Elemental analysis and determination of metal contents were performed using a Thermo Scientific iCAP 6000 Series ICP spectrometer and a Thermo Scientific Flash 2000 organic elemental analyser. The preparation for elemental analysis follows this method: the accurately weighed sample was placed into a 12 mL Teflon‐lined stainless‐steel autoclave, and 750 μL concentrated HNO_3_ and 2250 μL concentrated HCl were added. The autoclave was sealed and heated at 145 °C for 12 h. After cooling to room temperature, the mixture was diluted with deionised water and transferred to a 10 mL volumetric flask, and used for elemental analysis.

**CO‐DRIFTs measurement**. Diffuse reflectance infrared Fourier transform spectra of CO (CO‐DRIFTs)^[20]^ adsorbed on the catalyst were measured on a spectrometer (FT/IR‐6100, Jasco) equipped with a mercury‐cadmium‐tellurium (MCT) detector at a resolution of 4 cm^−1^. The samples were pre‐treated with N_2_ flow (20 mL min^−1^) at 160 °C for 2 h. After pre‐treatment, the sample was cooled to 25 °C in a N_2_ flow and measured as the background. Pure CO (99.99999 %, 20 mL min^−1^) was introduced to the system for 7 min, and a flow of N_2_ for 40 mins was used to remove adsorbed CO. The FT‐IR spectra of chemisorbed CO were recorded at 25 °C.

**NMR measurement**. ^1^H NMR and ^13^C NMR spectra were collected at room temperature in CDCl_3_ on Bruker B500 or B400 spectrometers.

**Synchrotron X‐ray powder diffraction measurement**. Collection of synchrotron X‐ray powder diffraction data was undertaken on Beamline I11 Diamond Light Source (Oxford, UK) [λ=0.826562(2) Å]. Desolvated Ru/MFM‐300(Cr) was obtained by heating the solvated sample under dynamic vacuum at 120 °C overnight. To prepare the substrate‐loaded samples, the desolvated Ru/MOF was dispersed in benzaldehyde (liquid) for 2 days, and the substrate‐loaded Ru/MOF was isolated and dried at room temperature. Other loaded MOF materials were prepared using the same procedure. Powder samples were loaded in a 0.7 mm borosilicate glass capillary, and high‐resolution synchrotron PXRD data were collected in the 2θ range 0–150° with 0.002° data steps using multi‐analyser crystal detectors at 25.0 °C. The PXRD patterns were refined using the Rietveld method in the TOPAS software. Stephen fitting^[33]^ was applied to describe the diffraction peaks and their anisotropic broadening. The scale factor and lattice parameters were allowed to refine for all the diffraction patterns. The refined structural parameters include the fractional coordinates (x, y, z) and isotropic displacement factors for all the atoms, and the site occupancy factors for guest molecules. The final stage of the Rietveld refinement involved soft restraints to the C−C bond lengths within the benzene rings, and rigid body was applied to the guest molecules in the pore. The quality of the Rietveld refinements was assured with low weighted profile factors and well‐fitting patterns with reasonable isotropic displacement factors within experimental errors. Trace amounts of free water molecules were found in the structures of **2a**‐loaded and **3a**‐loaded Ru/MFM‐300(Cr), interacting with adsorbed carbonyl substrates via hydrogen bonding.

**FTIT measurement**. FTIR spectra were recorded using a Bruker Alpha II FT‐IR spectrophotometer. Substrate‐loaded samples were prepared using the same method as described for the synchrotron X‐ray powder diffraction experiments.

**NH_3_ adsorption measurement**. Gravimetric sorption isotherms of NH_3_ were recorded at 273 and 293 K, maintained using a temperature‐programmed water bath and furnace, on a Hiden Isochema IGA‐003 system under ultra‐high vacuum (10^−9^ bar) using a turbo pumping system. Ultra‐pure research grade (99.999 %) NH_3_ was purchased from BOC. In a typical gas adsorption experiment, acetone‐exchanged MFM‐300(Cr) or Ru/MFM‐300(Cr) (80 mg) was loaded into the IGA system and activated at 423 K under dynamic high vacuum (10^−9^ bar) for 24 h to give fully desolvated MFM‐300(Cr) or Ru/MFM‐300(Cr).

**In situ XPS measurement**. XPS spectra were recorded with a SPECS Near Ambient Pressure XPS system employing a monochromatic Al Kα source (1486.6 eV) and a ‘Devi‐Sim’ cell‐type NAP environment attached to a SPECS Phoibos 150 NAP differentially pumped analyser. For XPS analysis, the powders were pressed into a gold foil substrate. The spectra were recorded at a pass energy of 30 eV and charge corrected to the main component of the C 1s peak at 284.8 eV for sp^3^ carbon in adventitious carbon contamination. In situ reduction of the catalyst was performed at 2 mbar H_2_ and the temperatures specified in Figure 2a, as measured by a type K thermocouple spot‐welded to the sample plate.

**XAS measurement**. The X‐ray absorption spectra (XAS) including X‐ray absorption near edge structure (XANES) and extended X‐ray absorption fine structure (EXAFS) at Ru K‐edge of the samples were measured on the B18 beamline at the Diamond Light Source, Didcot, UK. Measurements were performed in transmission mode using a QEXAFS setup with fast‐scanning Si(111) double crystal monochromators for the Ru K‐edge. Data were processed using the Athena and Artemis programs of the IFEFFIT package based on FEFF 6.[^34^, ^35^] Prior to merging, spectra were calibrated against the reference spectra by aligning the first peaks in the smoothed first derivative of the absorption spectrum, with background removed, and processed to obtain a normalized unit edge step. Fitting of the EXAFS regions were performed using the Artemis program of the IFEFFIT package. Fittings were performed with a k‐weight of 2 in R‐space. Refinement was performed by optimizing an amplitude factor *S*_0_^2^ and energy shift ΔE_0_, which are common to all paths, in addition to parameters for bond length (R) and Debye‐Waller factor (σ^2^).

**Transmission electron microscopy (TEM) and scanning transmission electron microscopy (STEM)**. TEM and STEM characterisation used a Thermo Fisher Talos F200A equipped with a Schottky field emission fun (FEG) and Super‐X energy dispersive x‐ray spectroscopy (EDS) system. The TEM data were acquired at 200 kV with a defocused probe and a total probe current of 400 pA. STEM data were obtained with a probe semi‐convergence angle of 10.5 mrad, a probe current of 734 pA, and an annular dark field detector inner angle of 22.5 mrad. STEM‐EDS mapping was obtained with a dwell time of 25 μs per pixel, and a total acquisition time of 955 s obtained over 240 frames.

**ssNMR measurement**. Solid‐state NMR (ssNMR) spectra were recorded using a Bruker 9.4 T (400 MHz ^1^H Larmor frequency) AVANCE III spectrometer equipped with a 4 mm HFX MAS probe. Samples were treated and packed into 4 mm o.d. zirconia rotors under inert conditions and sealed with a Kel‐F rotor cap. Experiments were acquired at ambient temperature using a MAS frequency of 12 kHz and the number of scans varied from 352 [for NH_4_ formate {^1^H‐}^13^C cross‐polarisation (CP)] to 3456 [for the sample after reaction of NH_3_/CO_2_/H_2_ {^1^H‐}^13^C cross‐polarisation (CP)]. For all experiments, ^1^H‐pulses of 100 kHz and ^13^C‐pulses of 50 kHz were used. For CPMAS NMR, ^13^C spin‐locking at ~50 kHz was applied for 2 ms, with corresponding ramped (70‐100 %) ^1^H spin‐locking at ~73 kHz, along with 100 kHz of SPINAL‐64^[36]^ heteronuclear ^1^H decoupling throughout signal acquisition. For the 2D ^1^H‐^13^C FSLG‐HETCOR^[37]^ dipolar correlation experiment, 1280 transients were acquired for each of 28 complex *t*_1_ increments, with a mixing time of 0.5 ms. The ^13^C NMR chemical shifts were referenced to neat TMS externally.

### N‐formylation of carbonyl compounds with CO_2_ and H_2_ in NH_3_/MeOH

In a typical procedure, the catalyst was added to 2 mL of NH_3_/MeOH (7 N) and 1 mmol of carbonyl compound was added into the reactor which was then sealed. The reactor was flushed with CO_2_ three times to remove air, and CO_2_ (3 MPa) and H_2_ (4 MPa) were introduced into the reactor and heated at 160 °C for 16 h under stirring. When the reaction was completed, the reactor was cooled to room temperature using an ice bath and degassed slowly to ambient pressure. The yield of resultant product was measured by GC, NMR spectroscopy or isolated by column chromatography.

## Supporting Information

PXRD, N_2_, CO_2_ and NH_3_ adsorption‐desorption, TGA, In situ DRIFT, XPS, TPR, SPXRD, FT‐IR, solid state NMR, ICP‐AES, catalytic results, ^1^H and ^13^C NMR spectroscopic data. (PDF)

The authors have cited additional references within the Supporting Information.[^38^, ^39^, ^40^, ^41^, ^42^, ^43^, ^44^, ^45^]

## CCDC

Deposition Numbers 2211020 (for benzaldehyde‐loaded Ru/MFM‐300(Cr)), 2210901 (for acetophenone‐loaded Ru/MFM‐300(Cr)), 2210900 (for 4‐methoxybenzaldehyde‐loaded Ru/MFM‐300(Cr)), 2210903 (for biphenyl‐4‐carboxaldehyde‐loaded Ru/MFM‐300(Cr)), xxxxx (for X) contain the supplementary crystallographic data for this paper. These data are provided free of charge by the joint Cambridge Crystallographic Data Centre and Fachinformationszentrum Karlsruhe Access Structures service.

## Conflict of interest

The authors declare no conflict of interest.

1

## Supporting information

As a service to our authors and readers, this journal provides supporting information supplied by the authors. Such materials are peer reviewed and may be re‐organized for online delivery, but are not copy‐edited or typeset. Technical support issues arising from supporting information (other than missing files) should be addressed to the authors.

Supporting Information

## Data Availability

The data that support the findings of this study are available from the corresponding author upon reasonable request.
