# Supplementary material for: Direct Synthesis of N‐formamides by Integrating Reductive Amination of Ketones and Aldehydes with CO2 Fixation in a Metal‐Organic Framework
Source: Chemistry. 2023 Dec 12;30(7):e202303289. doi: 10.1002/chem.202303289 (PMC10952134; doi:10.1002/chem.202303289)
Supplement: Supplementary file 1 — Supporting Information [file CHEM-30-0-s001.pdf]

Chemistry–A European Journal

Supporting Information

Direct Synthesis of N-formamides by Integrating Reductive Amination of Ketones and Aldehydes with CO_2 Fixation in a Metal-Organic Framework

Wenyuan Huang, Qingqing Mei, Shaojun Xu, Bing An, Meng He, Jiangnan Li, Yinlin Chen, Xue Han, Tian Luo, Lixia Guo, Joseph Hurd, Daniel Lee, Evan Tillotson, Sarah J. Haigh, Alex Walton, Sarah J. Day, Louise S. Natrajan, Martin Schröder,* and Sihai Yang*

Table of Contents

1. **Supplementary Figures**
2. **Supplementary Tables**
3. **^{1}H and ^{13}C NMR Results**
4. **References**

1. Supplementary Figures

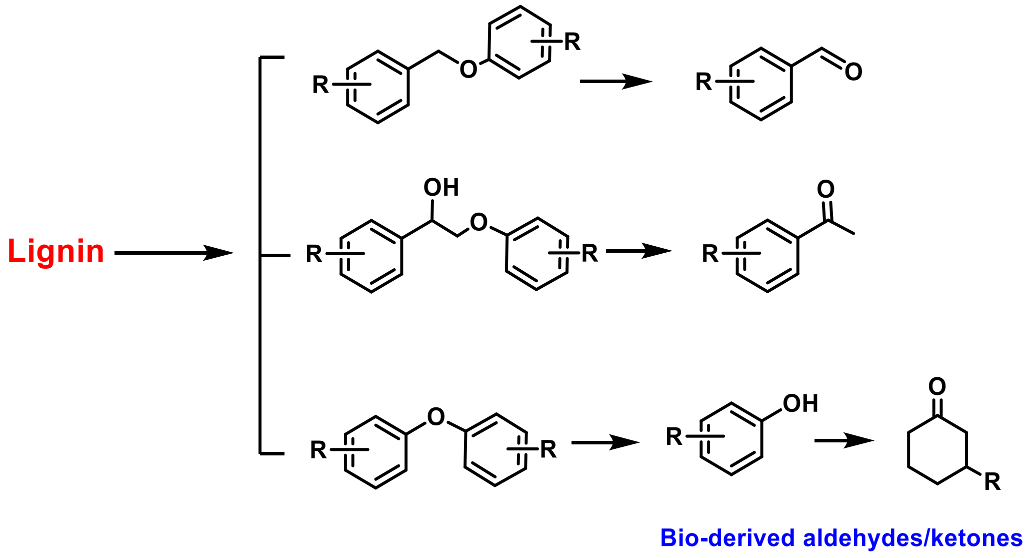

Figure S1: Examples of aldehydes and ketones derived from lignin.

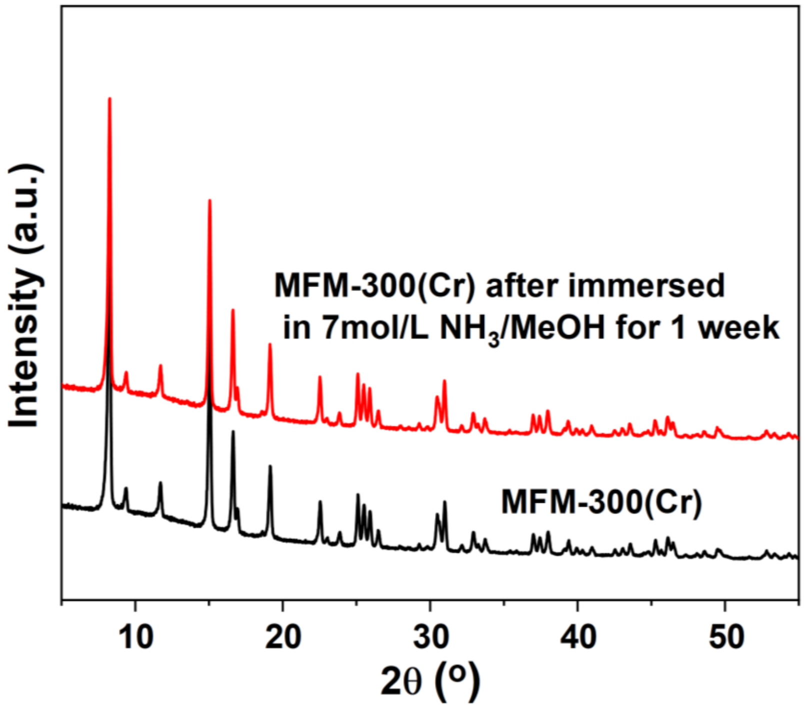

Figure S2: PXRD patterns of (i) MFM-300(Cr), (ii) MFM-300(Cr) after immersed in 7 mol/L NH_3/MeOH for 1 week.

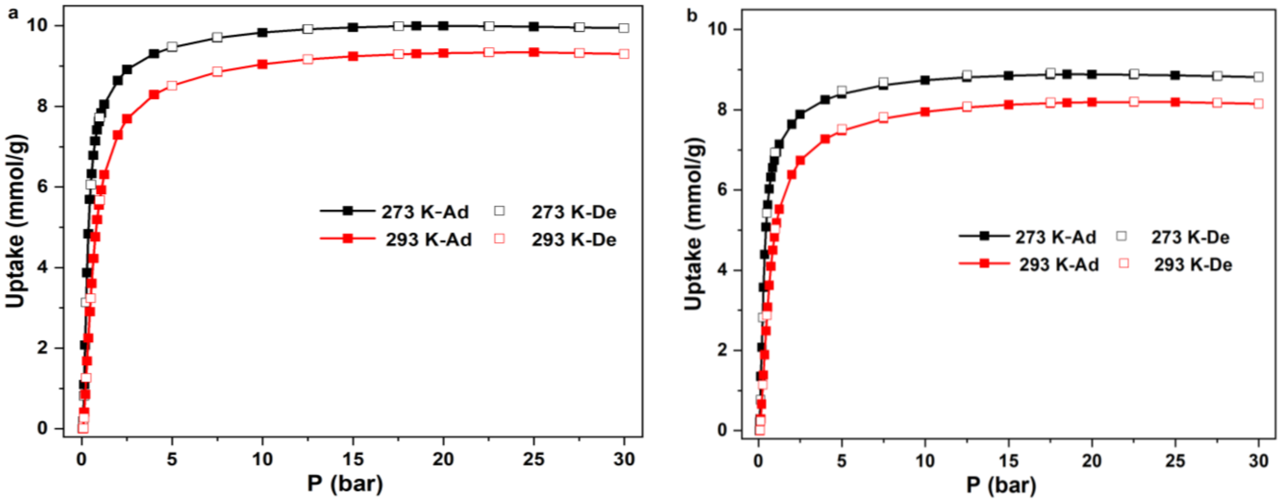

Figure S3: CO_2 adsorption-desorption isotherms of (a) MFM-300(Cr) and (b) Ru/MFM-300(Cr) at 273 and 293 K.

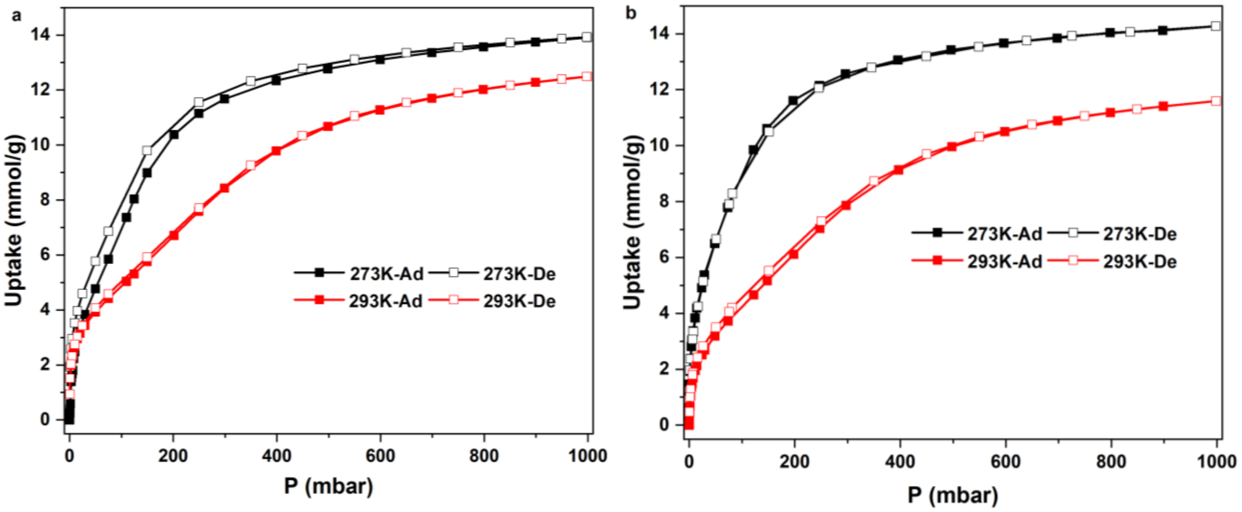

Figure S4: NH_3 adsorption-desorption isotherms of (a) MFM-300(Cr) and (b) Ru/MFM-300(Cr) at 273 and 293 K.

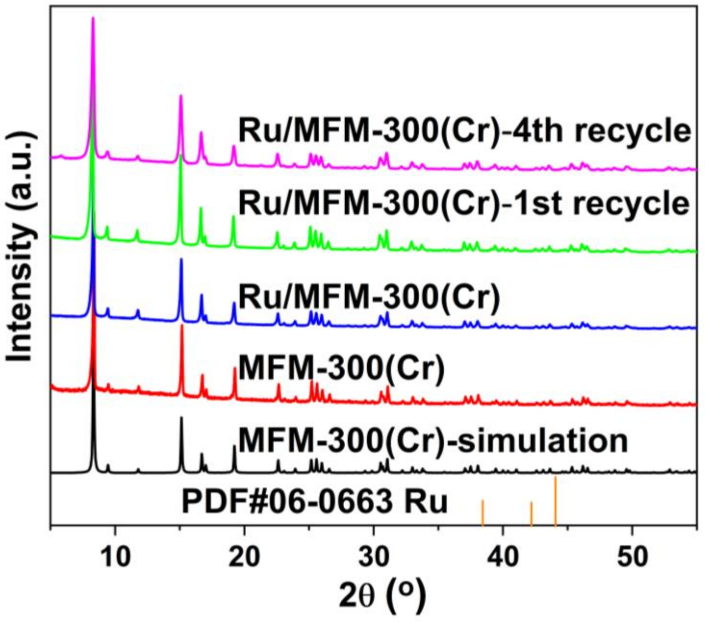

Figure S5: PXRD patterns of MFM-300(Cr), fresh Ru/MFM-300(Cr), Ru/MFM-300(Cr) after N-formylation of **1a** after first cycle (16h), and Ru/MFM-300(Cr) after the N-formylation of **1a** after the fourth cycle (64 h).

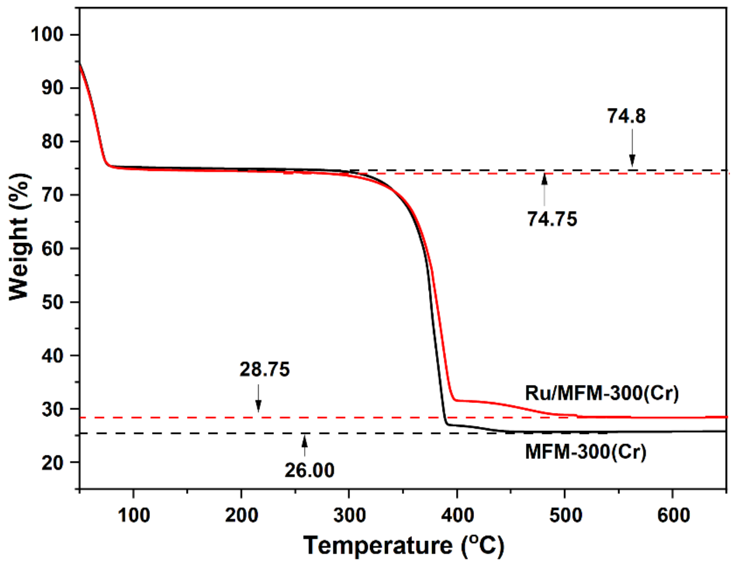

Figure S6: TGA plots of MFM-300(Cr) and Ru/MFM-300(Cr) samples.

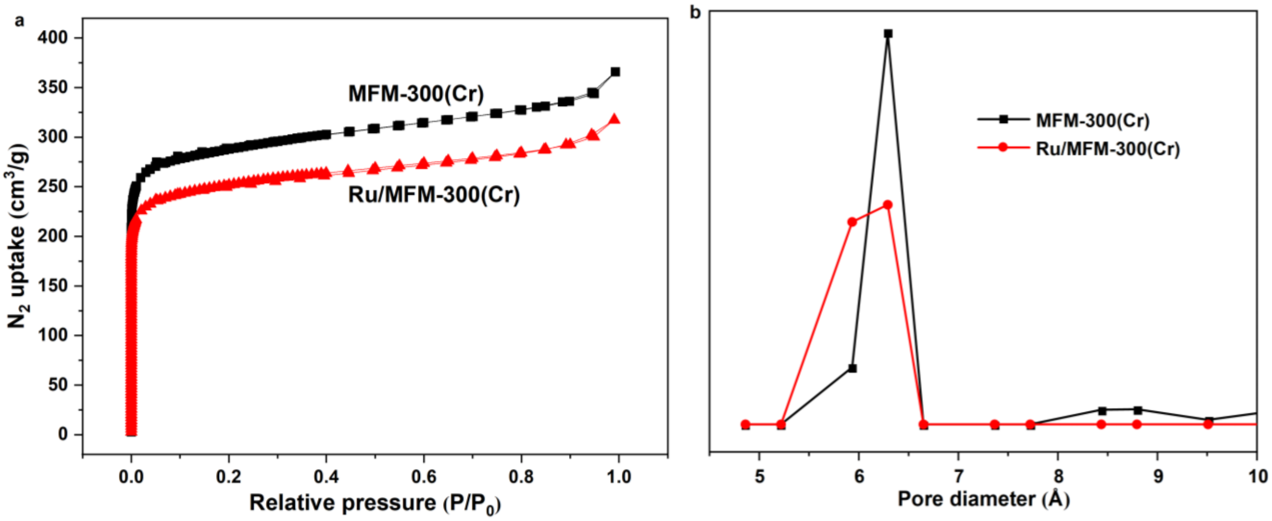

Figure S7: N_2 sorption isotherms (a) and pore size distribution (b) of MFM-300(Cr) and Ru/MFM-300(Cr) at 77 K.

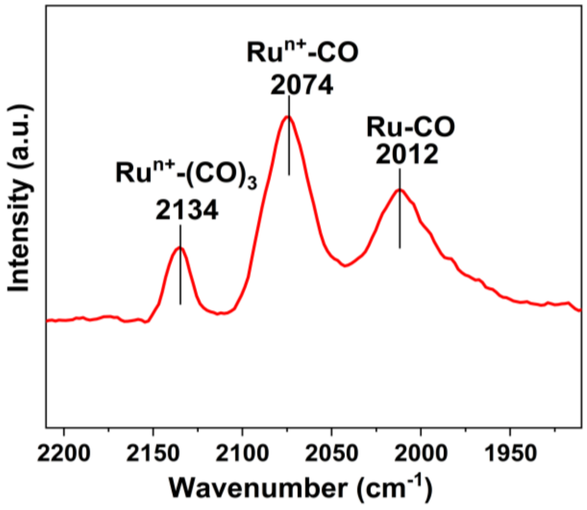

Figure S8: *In situ* DRIFT spectra of CO adsorbed on Ru/MFM-300(Cr).

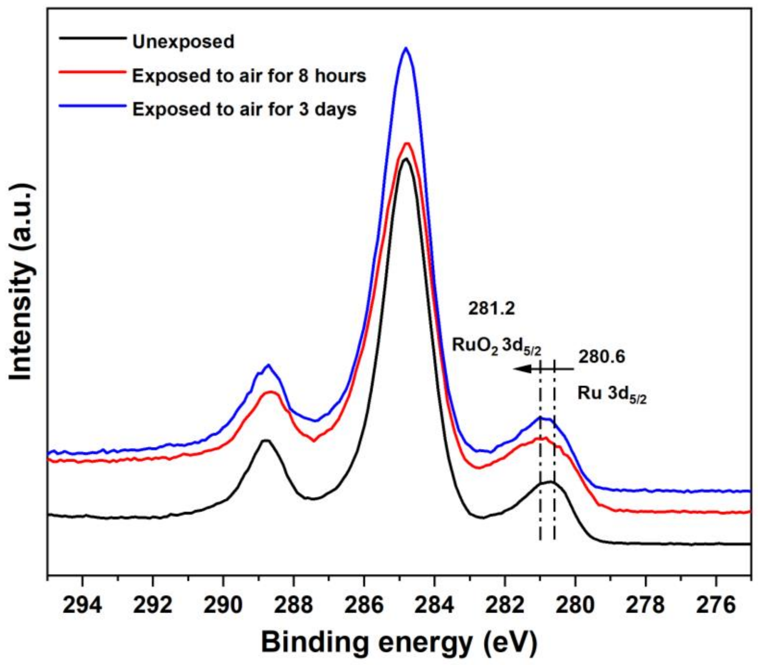

Figure S9: Ru XPS spectra of Ru/MFM-300(Cr) unexposed to air or exposed to air for 8 h or 3 days. The Ru/MFM-300(Cr) unexposed to air shows signal attributed to metallic Ru (Ru $3d_{5/2}$, 280.6 eV), while both samples exposed to air reveal combined signals of metallic (Ru $3d_{5/2}$, 280.6 eV) and oxidised Ru species (RuO_2 $3d_{5/2}$, 281.2 eV), suggesting partial oxidation of Ru occurs on exposure to air. In addition, the sample exposed to air for 3 days has a higher degree of oxidation, consistent with the TPR result. (The other two peaks located around 288.8 and 285.0 eV can be attributed to C 1s of MOF MFM-300(Cr)).

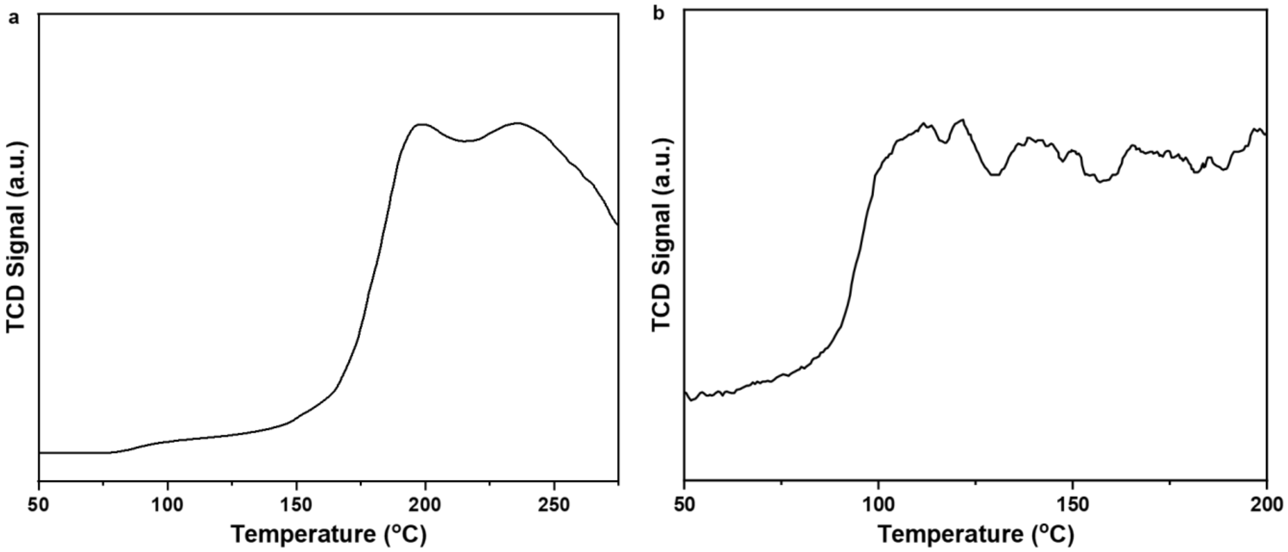

Figure S10: Temperature programmed reduction (TPR) profile of a) $RuCl_3{\cdot}xH_2O$/MFM-300(Cr) and b) Ru/MFM-300(Cr) exposed to air for 3 days. Samples of Ru/MFM-300(Cr) require no more than 125 ^{o}C for complete reduction. Due to the N-formylation reaction being performed at 160 ^{o}C, the oxidised Ru centres are reduced during the reaction.

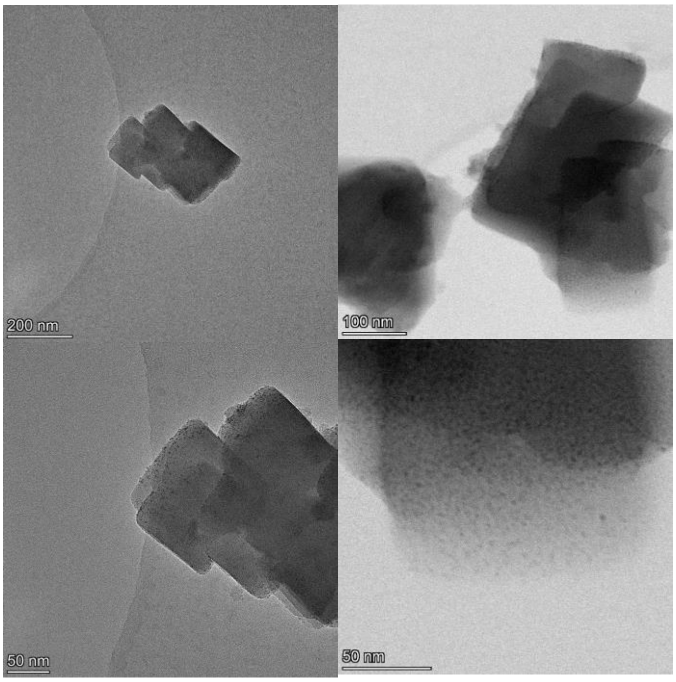

Figure S11: STEM images of Ru/MFM-300(Cr).

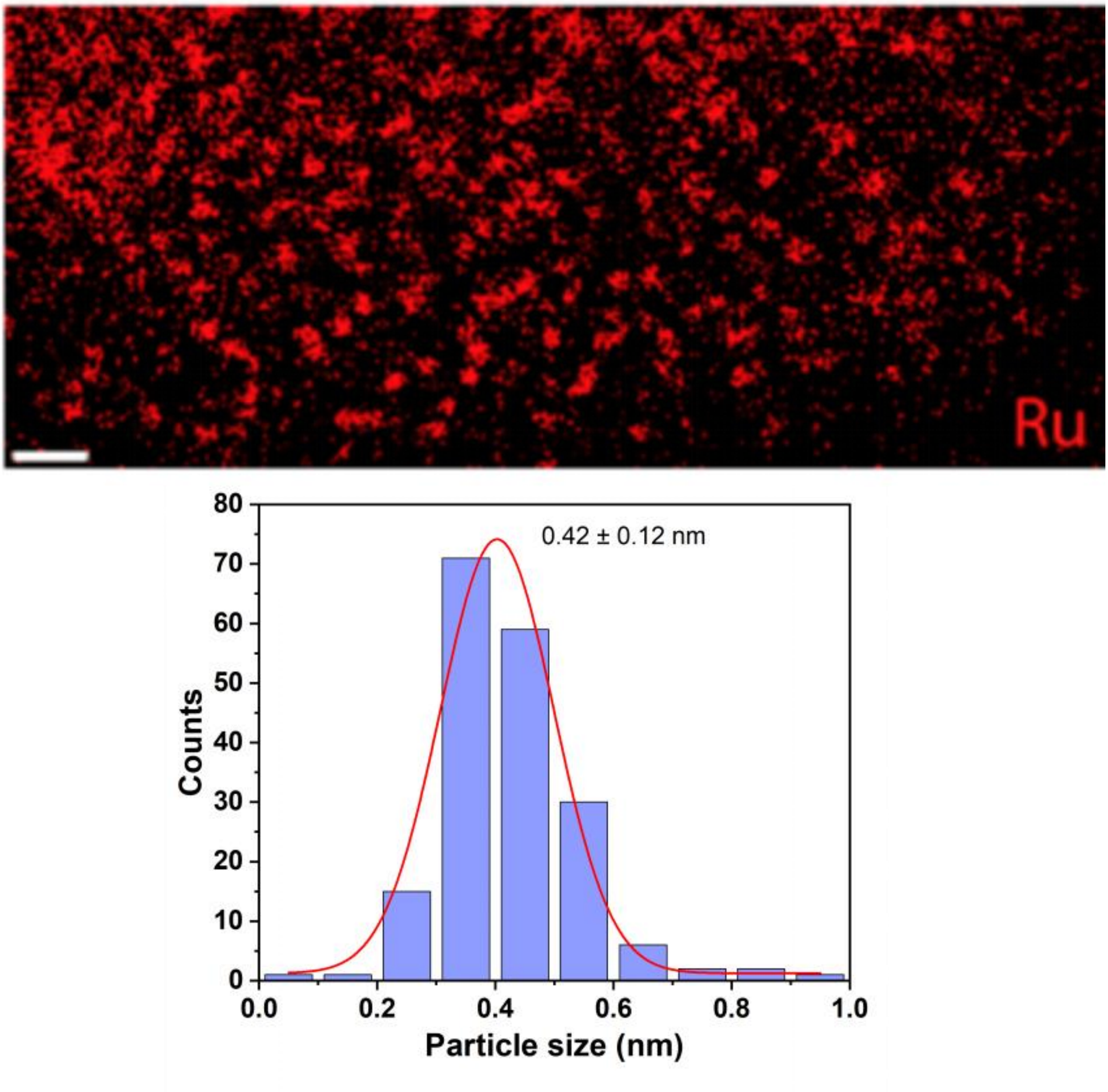

Figure S12: Particle size distribution (bottom) extracted from EDS mapping of Ru (up).

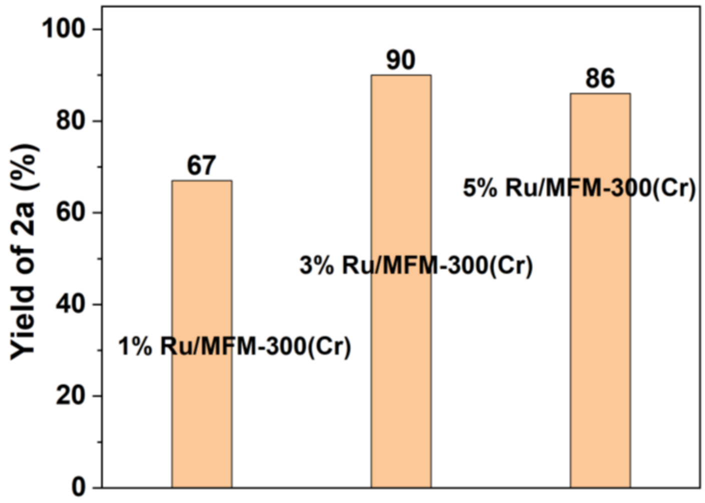

Figure S13: Catalytic study of N-benzylformamide (**2a**) produced with different amount Ru (1wt%, 3wt% and 5wt%)-loaded MFM-300(Cr).

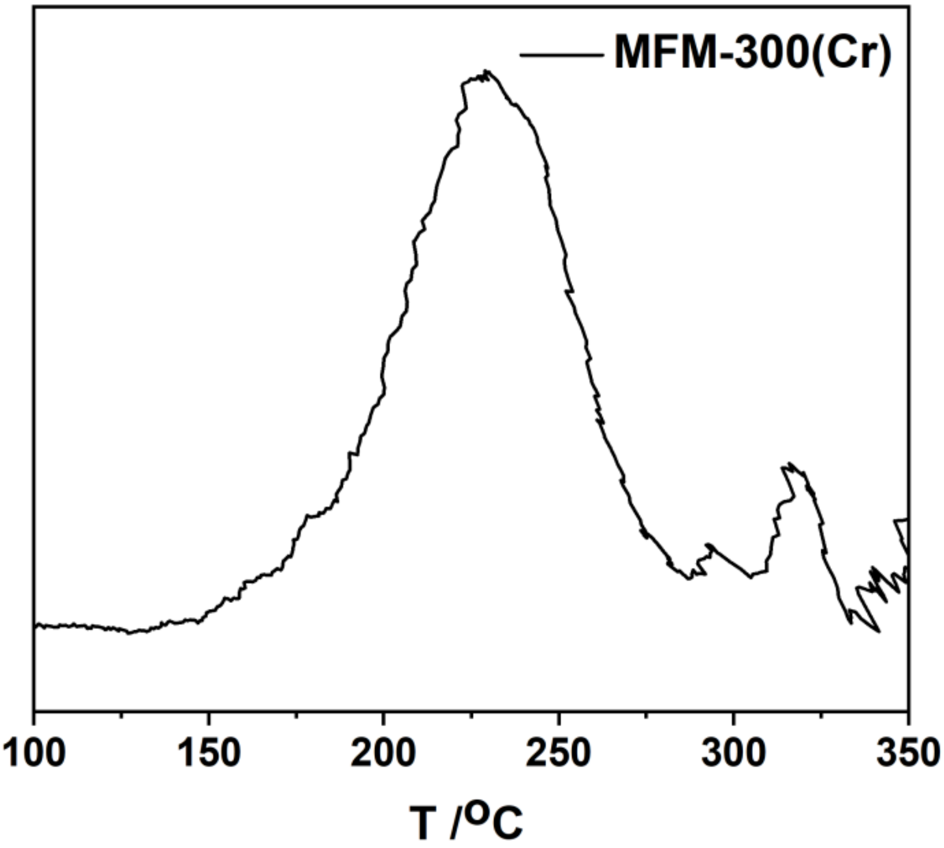

Figure S14: NH_3-TPD plots of MFM-300(Cr).

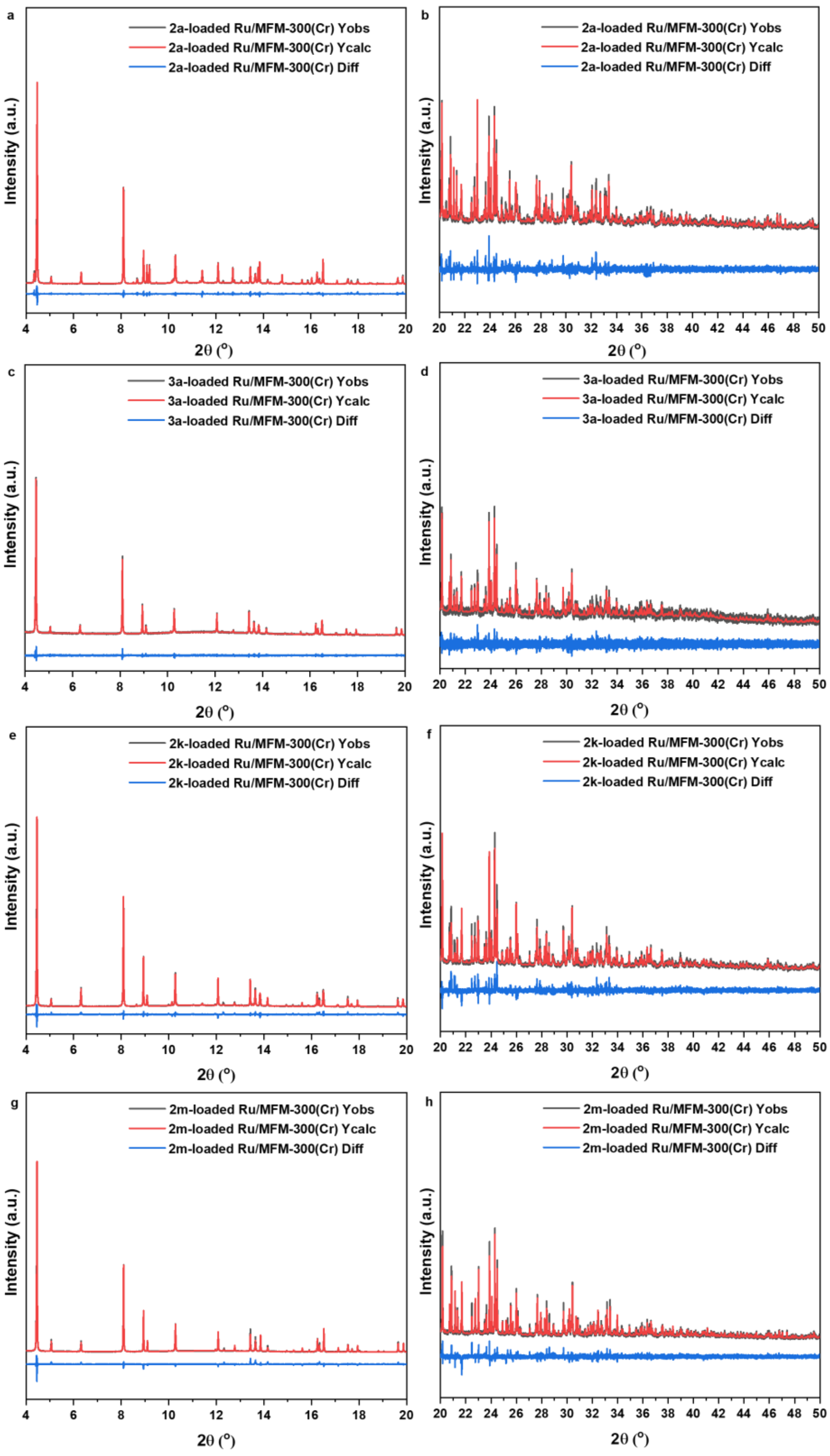

Figure S15: Experimental data (black line) and Rietveld refinement (red line) and the difference pattern (blue line) for SPXRD patterns of (a,b) **2a**-loaded Ru/MFM-300(Cr) (c,d) **3a**-loaded Ru/MFM-300(Cr), (e,f) **2k**-loaded Ru/MFM-300(Cr) and (g,h) **2m**-loaded Ru/MFM-300(Cr) at room temperature at a 2θ range of (a,c,e,g) 3 - 20° and (b,d,f,h) 20 - 50° [λ = 0.826562(2) Å]. Benzaldehyde (2a), acetophenone (3a), 4-methoxybenzaldehyde (2k) and biphenyl-4-carboxaldehyde (2m).

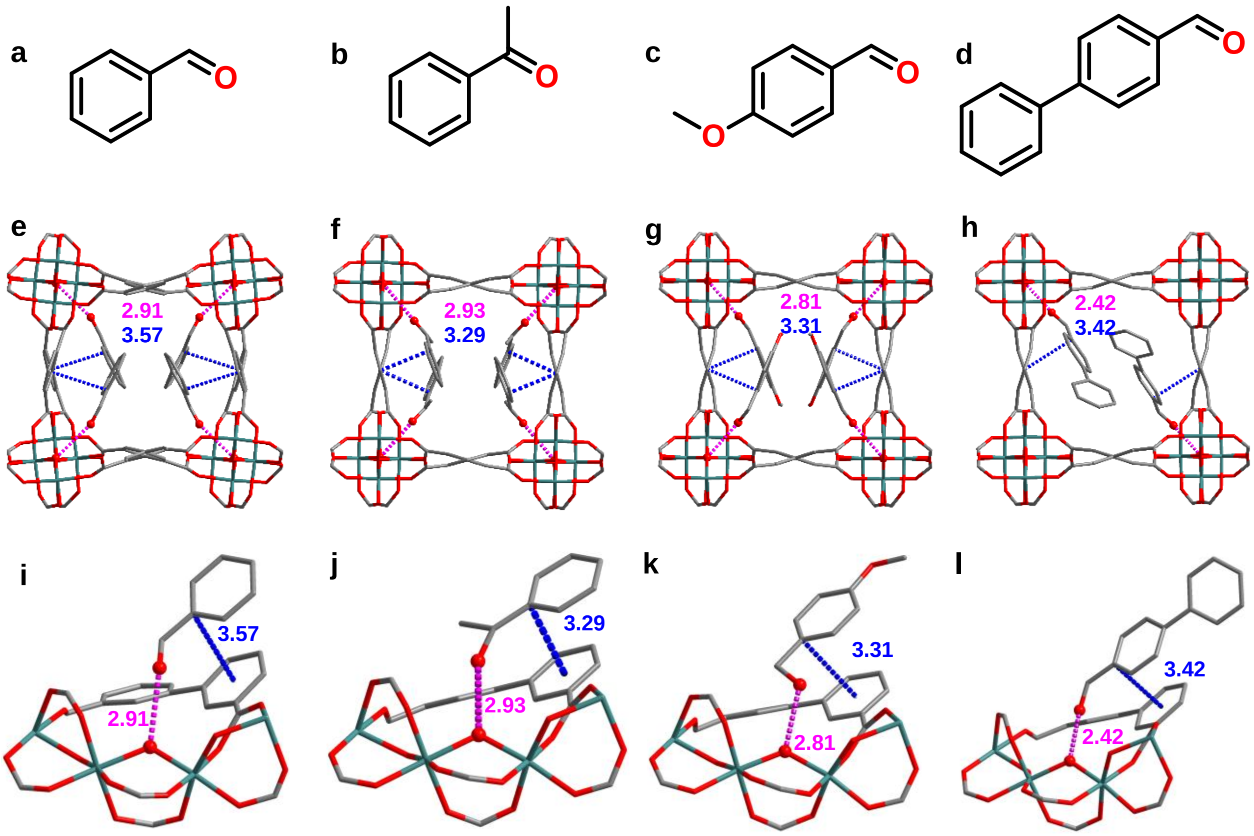

Figure S16: Views of crystal structures of Ru/MFM-300(Cr) loaded with benzaldehyde (2a), acetophenone (3a), 4-methoxybenzaldehyde (2k), biphenyl-4-carboxaldehyde (2m). All models were obtained from Rietveld refinements based on SPXRD data. Hydrogen atoms are omitted for clarity. Distances for hydrogen bonding are given as the donor-to-acceptor distances given the uncertainty in the position of protons. (a-d) Chemical structures of **2a**, **3a**, **2k** and **2m**; (e-h) view of the structures of **2a**-loaded, **3a**-loaded, **2k**-loaded and **2m**-loaded Ru/MFM-300(Cr), respectively; (i-l) views of host-guest binding of adsorbed **2a**, **3a**, **2k** and **2m** within Ru/MFM-300(Cr). Minor amounts of free water molecules in (e, f, g, h) are omitted for clarity.

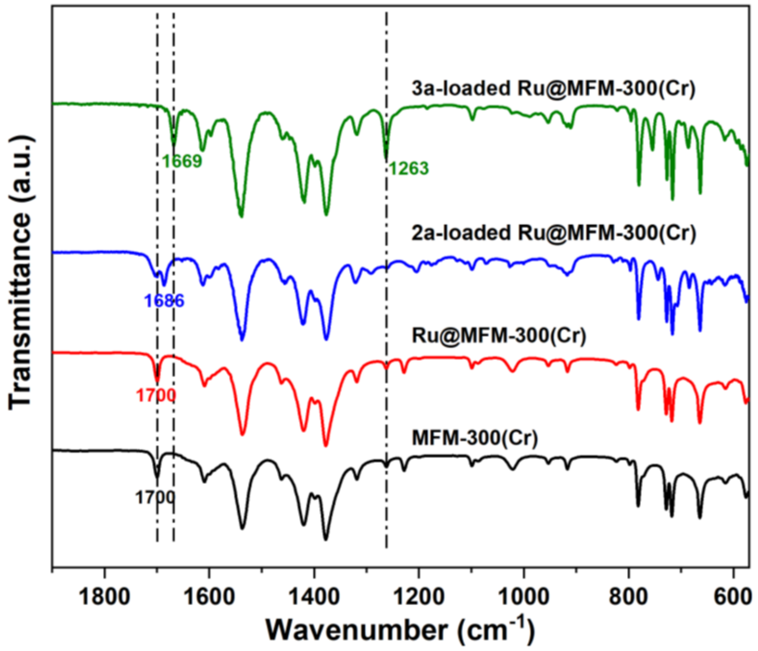

Figure S17: FT-IR spectra of as-synthesised MFM-300(Cr) (black), Ru/MFM-300(Cr) (red), **2a**-loaded Ru/MFM300(Cr) (blue) and **3a**-loaded Ru/MFM-300(Cr) (green) in the region of 2000-1000 cm^{-1}. Benzaldehyde (2a) and acetophenone (3a).

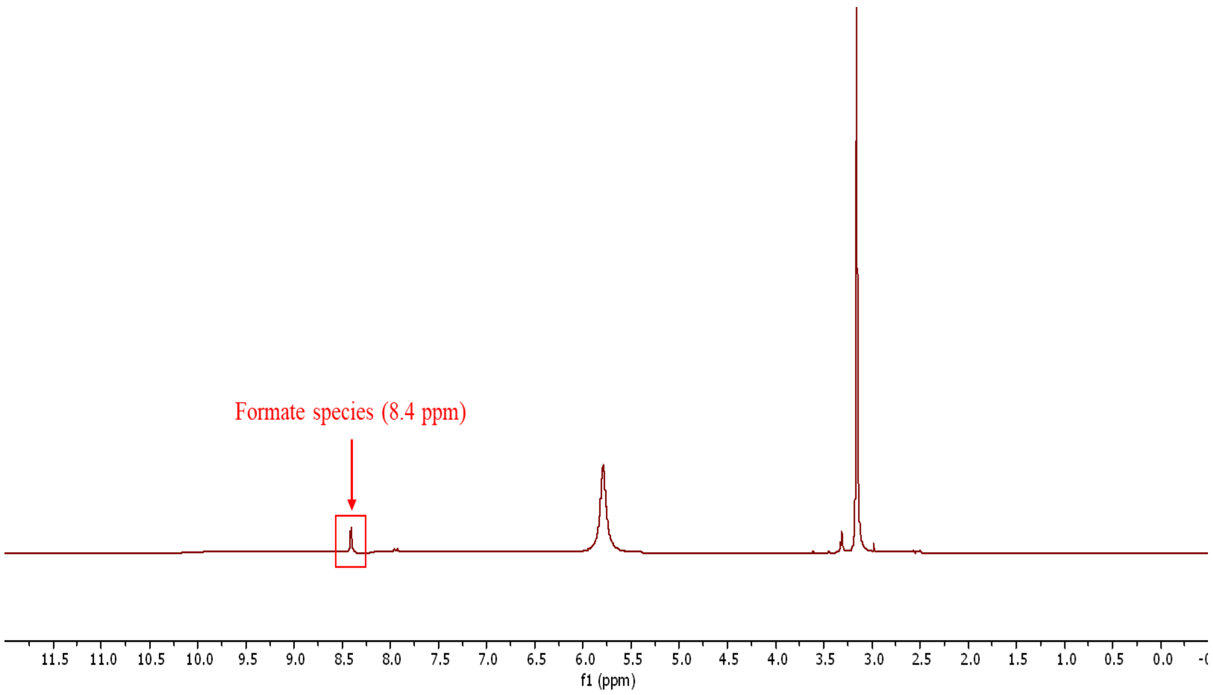

Figure S18: ^{1}H NMR spectra (400 MHz, DMSO-d_6) of the reaction mixture of catalyst suspension after catalysis (catalyst + NH_3/MeOH + CO_2 + H_2). Reaction conditions: catalyst (10 mg), NH_3/MeOH (7 mol/L, 2 mL), CO_2 (3 MPa), and H_2 (4 MPa), 433 K.

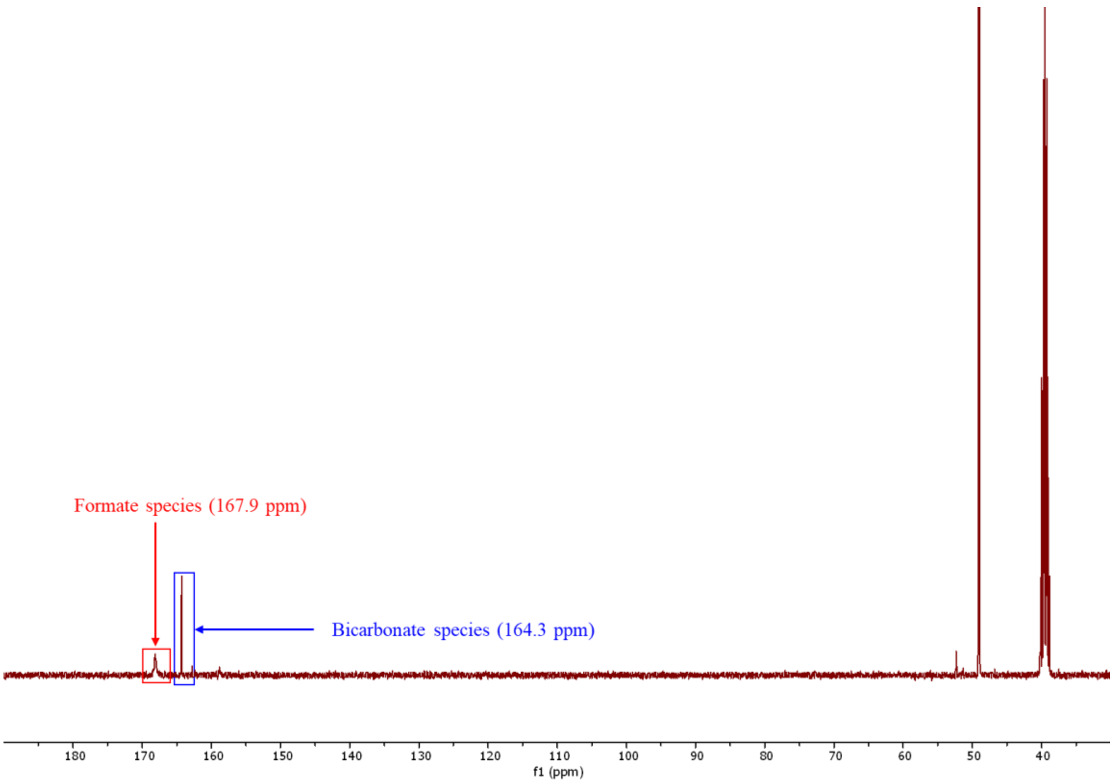

Figure S19: ^{13}C NMR spectra (400 MHz, DMSO-d_6) of the reaction mixture of catalyst suspension after catalysis (catalyst + NH_3/MeOH + CO_2 + H_2). Reaction conditions: catalyst (10 mg), NH_3/MeOH (7 mol/L, 2 mL), CO_2 (3 MPa), and H_2 (4 MPa), 433 K.

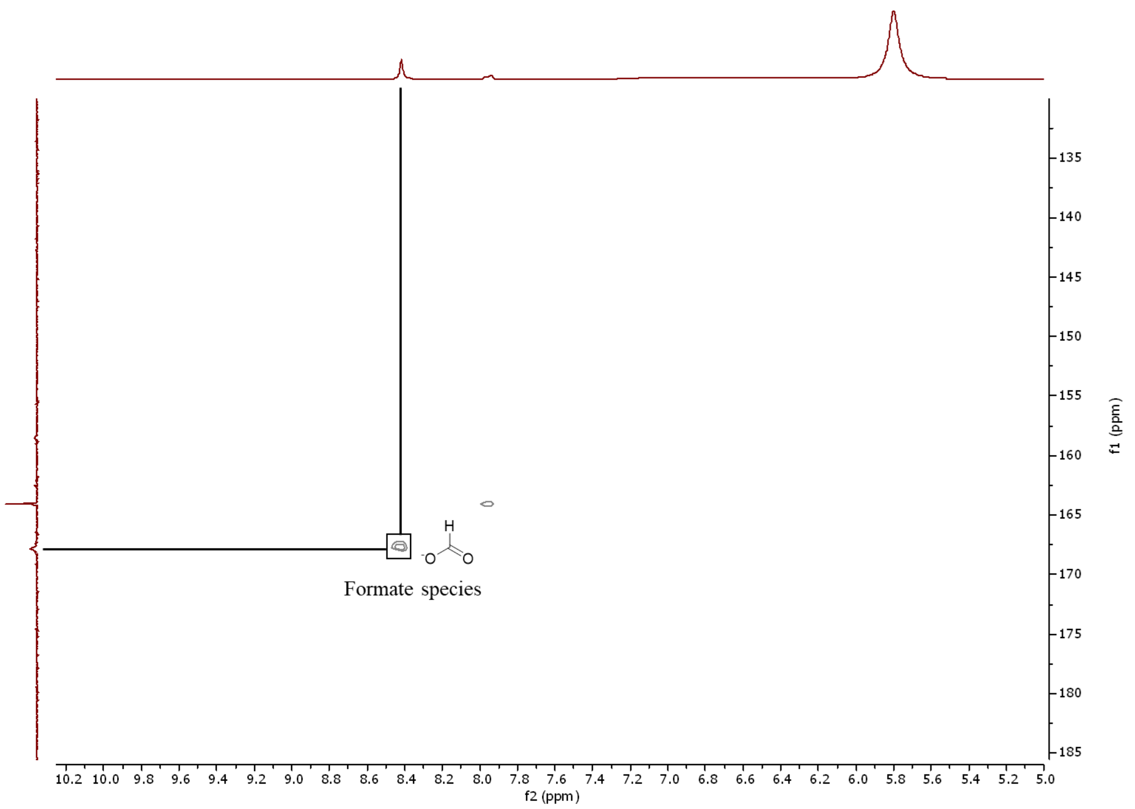

Figure S20: 2D HSQC NMR spectra (400 MHz, DMSO-d_6) of the reaction mixture of catalyst suspension after catalysis (catalyst + NH_3/MeOH + CO_2 + H_2). Reaction conditions: catalyst (10 mg), NH_3/MeOH (7 mol/L, 2 mL), CO_2 (3 MPa), and H_2 (4 MPa), 433 K.

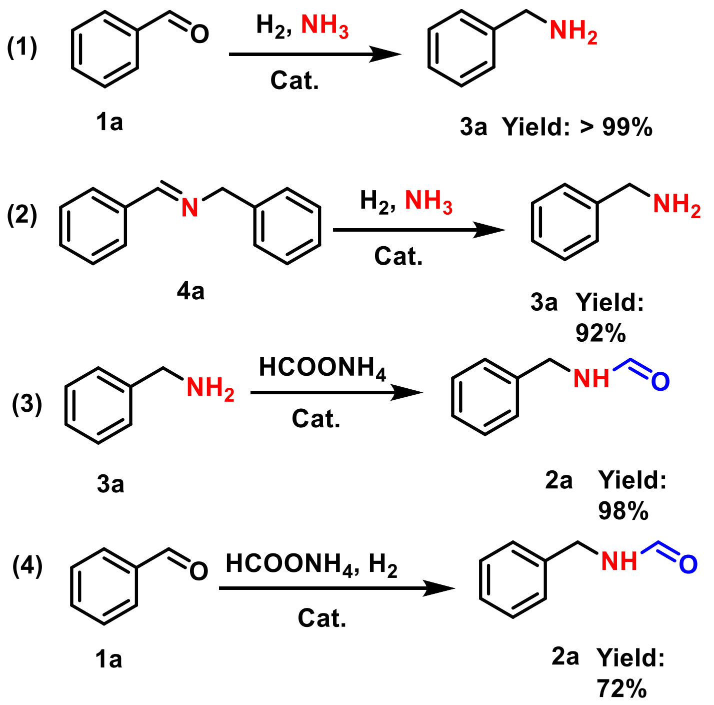

Figure S21: Analysis of the reaction pathway over Ru/MFM-300(Cr). Yields were determined by GC analysis.

2. Supplementary Tables

Table S1. Elemental analysis of Ru/support catalysts.

Samples	Loading amount of metal (wt%)
Ru/MFM-300(Cr)	2.9
Ru/MFM-300(Al)	2.8
Ru/Cr_2O_3	2.5
Ru/Al_2O_3	2.8
Ru/ZrO_2	2.9
Rh/MFM-300(Cr)	1.7
Pd/MFM-300(Cr)	3.1

Table S2. First shell EXAFS fitting for various samples.

Sample	path	CN[a]	R (Å)	ΔE_0 (eV)	S_0^2	σ^2	R factor
Ru/MFM-300(Cr)-as synthesised	Ru-Ru	2.36±0.56	2.70±0.04	-0.25±1.45	1 [b]	0.009±0.002	0.008
	Ru-Cl	0.60±0.06	2.35±0.008			0.009±0.011	
	Ru-O	5.90±1.26	2.02±0.05			0.016±0.003	
Ru/MFM-300(Cr)-used	Ru-Ru	2.51±0.40	2.70±0.03	-0.62±1.03	1 [b]	0.007±0.001	0.005
	Ru-Cl	0.70±0.06	2.34±0.03			0.012±0.014	
	Ru-O	7.88±1.51	2.02±0.06			0.020±0.004	
$RuCl_3$	Ru-Cl	6	2.36±0.02	4.74±0.52	1 [b]	0.003±0.0009	0.009
Ru foil	Ru-Ru	12	2.67±0.005	-5.46±1.22	0.99	0.002±0.001	0.016

[a]CN: coordination number; [b]set value = 1.

Calculation of particle size from EXAFS results. The coordination numbers (CN) from EXAFS fitting for Ru/MFM-300(Cr) were converted to particle sizes using the following formula developed by Miller et al[1]:

$\text{Log}_{10}(1000/d) = 2.5763 - 0.1319 \times (\text{CN})$

The CN of Ru-Ru (2.36 ± 0.56) in Ru/MFM-300(Cr)-as synthesised gave an average particle size of 0.54 ± 0.29 nm, and the CN of Ru-Ru (2.51 ± 0.40) in Ru/MFM-300(Cr)-used gave an average particle size of 0.57 ± 0.30 nm, indicating that the size of Ru nanoparticles does not change significantly on reaction.

Table S3. Crystallographic data and details of bare and substrate-loaded Ru/MFM-300(Cr).

Sample	**2a**-loaded Ru/MFM-300(Cr)	**3a**-loaded Ru/MFM-300(Cr)	**2k**-loaded Ru/MFM300(Cr)	**2m**-loaded Ru/MFM300(Cr)
CCDC	2211020	2210901	2210900	2210903
Crystal system	Tetragonal	Tetragonal	Tetragonal	Tetragonal
Space group	$P4_12_12$	$I4_122$	$I4_122$	$I4_122$
Chemical formula	$CrC_8O_5H_4 \cdot 0.768\ C_7OH_6 \cdot 0.923\ H_2O$	$CrC_8O_5H_4 \cdot 0.644\ C_8OH_8 \cdot 1.276\ H_2O$	$CrC_8O_5H_4 \cdot 0.782\ C_8O_2H_8$	$CrC_8O_5H_4 \cdot 0.500\ C_{13}OH_{10}$
2θ range for refinement	3-50°	3-50°	3-50°	3-50°
Refinement methods	Rietveld Method	Rietveld Method	Rietveld Method	Rietveld Method
a (Å)	14.97296(10)	14.99276(5)	15.00068(4)	14.99432(4)
c (Å)	11.97563(8)	11.97229(8)	11.97377(6)	11.95032(3)
V (Å^3)	2684.81(4)	2691.20(3)	2694.34(2)	2686.79(1)
R_{wp} (%)	7.391	7.251	7.921	8.152
R_p(%)	5.617	5.552	5.810	6.062
R_{Bragg}(%)	2.099	1.302	3.420	4.089
R_{exp} (%)	3.853	5.274	3.530	4.132
Gof χ^2	1.918	1.375	2.244	1.973

3. ^{1}H and ^{13}C NMR spectroscopic data

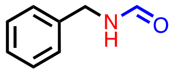

1

***N*-benzylformamide**[2]**:** ^{1}H NMR (400 MHz, $CDCl_3$) δ 8.26 (major rotamer, s, 0.85H), 8.17 (minor rotamer, d, *J* = 11.9 Hz, 0.15H), 7.40-7.27 (m, 5.00H), 6.14 (br s, 2H), 4.49 (major rotamer, d, *J* = 5.9 Hz, 1.7H), 4.42 (minor rotamer, d, *J* = 6.5 Hz, 0.3H);

^{13}C NMR (101 MHz, $CDCl_3$) major rotamer: δ 161.1, 128.8, 127.8, 127.7, 42.20; minor rotamer: δ 164.8,

137.6, 137.5, 129.0, 128.0, 127.0, 45.7,

HRMS (ESI): m/z $[M+H]^+$ calcd for $C_8H_{10}NO$ 136.0757, found 136.0754.

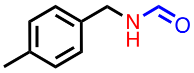

2

N-(4-methylbenzyl)formamide: ^{1}H NMR (500 MHz, $CDCl_3$) δ 8.17 (major rotamer, s, 0.85H), 8.08 (minor rotamer, d, *J* = 12.0 Hz, 0.15H), 7.16-7.10 (m, 4H), 6.31 (br s, 1H), 4.38 (major rotamer, d, *J* = 5.9 Hz, 1.7H), 4.31 (minor rotamer, d, *J* = 6.4 Hz, 0.3H), 2.34 (minor rotamer, s, 0.45H), 2.32 (major rotamer, s, 2.55H).

^{13}C NMR (126 MHz, $CDCl_3$) major rotamer: δ 161.2, 137.3, 134.6, 129.4, 127.8, 41.9, 21.1; minor rotamer: δ 164.8, 137.7, 134.5, 129.5, 126.9, 45.4.

HRMS (ESI): m/z $[M+Na]^+$ calcd for $C_9H_{11}ONNa$ 172.0733, found 172.0729.

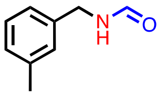

3

N-(3-methylbenzyl)formamide[3]: NMR Yield 77%. ^{1}H NMR (400 MHz, $CDCl_3$) δ 8.36 (major rotamer, s, 0.85H), 8.28 (minor rotamer, d, *J* = 11.9 Hz, 0.15H), 7.40-7.33 (m, 1H), 7.25-7.10 (m, 3H), 6.19 (br s, 1H), 4.56 (major rotamer, d, *J* = 5.8 Hz, 1.7H), 4.49 (minor rotamer, d, *J* = 6.5 Hz, 0.3H), 2.48 (minor rotamer, s, 0.45H), 2.47 (major rotamer, s, 2.55H).

^{13}C NMR (101 MHz, $CDCl_3$) major rotamer: δ 161.1, 138.6, 137.5, 128.7, 128.5, 124.9, 42.2, 21.3; minor rotamer: δ 164.8, 138.8, 137.4, 128.9, 128.6, 124.0, 45.7, 21.4.

HRMS (ESI): m/z $[M+H]^+$ calcd for $C_9H_{12}ON$ 150.0913, found 150.0911.

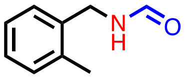

4

N-(2-methylbenzyl)formamide[3]: ^1H NMR (500 MHz, $CDCl_3$) δ 8.24 (major rotamer, s, 0.83H), 8.15 (minor rotamer, d, J = 12.0 Hz, 0.17H), 7.23-7.17 (m, 4H), 5.76 (br s, 1H), 4.48 (major rotamer, d, J = 5.4 Hz, 1.66H), 4.39 (minor rotamer, d, J = 6.2 Hz, 0.34H), 2.33 (major rotamer, s, 2.49H), 2.32 (minor rotamer, s, 0.51H).

^{13}C NMR (126 MHz, $CDCl_3$) major rotamer: δ 160.9, 136.5, 135.2, 130.7, 128.7, 128.1, 126.4, 40.4, 19.1; minor rotamer: δ 164.7, 135.7, 135.3, 130.8, 128.2, 127.4, 126.6, 43.7.

HRMS (ESI): m/z $[M+Na]^+$ calcd for $C_9H_{11}ONNa$ 172.0733, found 172.0729.

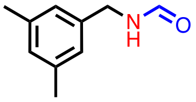

5

N-[(3,5-dimethylphenyl)methyl]formamide: ^1H NMR (400 MHz, $CDCl_3$) δ 8.20 (major rotamer, s, 0.84H), 8.10 (minor rotamer, d, J = 12.0 Hz, 0.16H), 6.96-6.80 (m, 3H), 6.23 (br s, 1H), 4.37 (major rotamer, d, J = 5.8 Hz, 1.68H), 4.29 (minor rotamer, d, J = 6.3 Hz, 0.32H), 2.31 (minor rotamer, s, 0.96H), 2.29 (major rotamer, s, 5.04H).

^{13}C NMR (101 MHz, $CDCl_3$) major rotamer: δ 161.1, 138.4, 137.4, 129.2, 125.6, 42.1, 21.2; minor rotamer: δ 164.8, 138.6, 137.5, 129.5, 124.8, 45.6.

HRMS (ESI): m/z $[M+H]^+$ calcd for $C_{10}H_{14}ON$ 164.1070, found 164.1066.

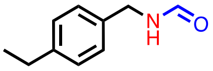

6

N-(4-ethylbenzyl)formamide: ^1H NMR (400 MHz, $CDCl_3$) δ 8.23 (major rotamer, s, 0.85H), 8.15 (minor rotamer, d, J = 11.9 Hz, 0.15H), 7.24-7.12 (m, 4H), 5.98 (br s, 1H), 4.43 (major rotamer, d, J

= 5.8 Hz, 1.7H), 4.36 (minor rotamer, d, *J* = 6.4 Hz, 0.3H), 2.63 (q, *J* = 7.6 Hz, 2H), 1.22 (t, *J* = 7.6 Hz, 3H).

^{13}C NMR (101 MHz, $CDCl_3$) major rotamer: δ 161.0, 143.9, 134.8, 128.3, 127.9, 42.0, 28.6, 28.5, 15.7; minor rotamer: δ 164.7, 144.2, 134.7, 128.5, 127.1, 45.5, 28.5.

HRMS (ESI): m/z $[M+H]^+$ calcd for $C_{10}H_{14}ON$ 164.1070, found 164.1067.

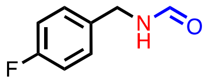

7

N-(4-Fluorobenzyl)formamide[4]: ^{1}H NMR (400 MHz, $CDCl_3$) δ 8.20 (major rotamer, s, 0.86H), 8.12 (minor rotamer, d, *J* = 11.9 Hz, 0.14H), 7.29-7.20 (m, 2H), 7.10-6.95 (m, 2H), 6.71 (br s, 1H), 4.41 (major rotamer, d, *J* = 6.0 Hz, 1.72H), 4.37 (minor rotamer, d, *J* = 6.4 Hz, 0.28H).

^{13}C NMR (101 MHz, $CDCl_3$) major rotamer: δ 163.4, 161.3, 133.5, 129.4, 115.5, 41.3; minor rotamer: δ 164.7, 160.90, 133.3, 128.7, 115.8, 45.0.

HRMS (ESI): m/z $[M+H]^+$ calcd for C_8H_9ONF 154.0663, found 154.0659.

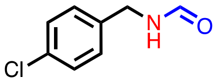

8

N-(4-Chlorobenzyl)formamide[4,5]: ^{1}H NMR (500 MHz, $CDCl_3$) δ 8.22 (major rotamer, s, 0.88H), 8.12 (minor rotamer, d, *J* = 11.9 Hz, 0.12H), 7.35-7.25 (m, 2H), 7.22-7.14 (m, 2H), 6.26 (br s, 1H), 4.40 (major rotamer, d, *J* = 6.1 Hz, 1.76H), 4.35 (minor rotamer, d, *J* = 6.5 Hz, 0.24H).

^{13}C NMR (126 MHz, $CDCl_3$) major rotamer: 161.2, 136, 133.5, 129.1, 128.9, 41.4. minor rotamer: δ 164.7, 136.0, 133.8, 131.8, 128.3, 45.0.

HRMS (ESI): m/z $[M-H]^-$ calcd for C_8H_7ONCl 168.0222, found 168.0204.

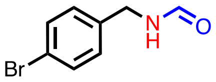

9

N-(4-bromobenzyl)formamide[3]: 1H NMR (500 MHz, $CDCl_3$) δ 8.22 (major rotamer, s, 0.87H), 8.11 (minor rotamer, d, J = 11.9 Hz, 0.13H), 7.50-7.41 (m, 2H), 7.16-7.07 (m, 2H), 6.29 (br s, 1H), 4.38 (major rotamer, d, J = 6.0 Hz, 1.74H), 4.34 (minor rotamer, d, J = 6.5 Hz, 0.26H).

^{13}C NMR (126 MHz, $CDCl_3$) major rotamer: δ 161.2, 136.7, 131.9, 129.5, 121.6, 41.5. minor rotamer: δ 164.7, 136.6, 132.1, 128.7, 121.9, 45.1.

HRMS (ESI): m/z $[M-H]^-$ calcd for C_8H_7ONBr 211.9716, found 211.9702.

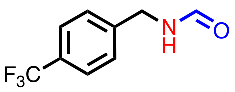

10

N-(4-(trifluoromethyl)benzyl)formamide: ^{1}H NMR (500 MHz, $CDCl_3$) δ 8.26 (major rotamer, s, 0.88H), 8.15 (minor rotamer, d, J = 11.8 Hz, 0.12H), 7.64-7.34 (m, 4H), 6.35 (br s, 1H), 4.50 (major rotamer, d, J = 6.1 Hz, 1.76H), 4.46 (minor rotamer, d, J = 6.6 Hz, 0.24H).

^{13}C NMR (126 MHz, $CDCl_3$) major rotamer: δ 161.3, 141.8, 129.9, 127.9, 125.7, 123.0, 41.6; minor rotamer: δ 164.8, 141.7, 129.0, 127.3, 125.9, 122.9, 45.2.

HRMS (ESI): m/z $[M+Na]^{2+}$ calcd for $C_{10}H_{10}ONF_3Na$ 240.0607, found 240.0601.

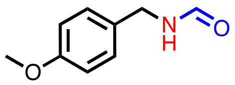

11

N-(4-Methoxybenzyl)formamide[4]**:** ^{1}H NMR (500 MHz, $CDCl_3$) δ 8.18 (major rotamer, s, 0.85H), 8.12 (minor rotamer, d, J = 11.9 Hz, 0.15H), 7.22-7.12 (m, 2H), 6.90-6.80 (m, 2H), 6.17 (br s, 1H), 4.37 (major rotamer, d, J = 5.7 Hz, 1.70H), 4.31 (minor rotamer, d, J = 6.3 Hz, 0.30H), 3.79 (minor rotamer, s, 0.45H), 3.77 (major rotamer, s, 2.55H).

^{13}C NMR (126 MHz, $CDCl_3$) major rotamer: δ 161.1, 159.1, 129.2, 128.4, 114.1, 55.3, 41.7; minor rotamer: δ 164.6, 159.3, 129.7, 114.3, 55.4, 45.2.

HRMS (ESI): m/z $[M+Na]^+$ calcd for $C_9H_{11}O_2Na$ 188.0678, found 188.0682.

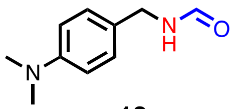

12

N-(4-(dimethylamino)benzyl)formamide[3]: ^{1}H NMR (500 MHz, $CDCl_3$) δ 8.18 (major rotamer, s, 0.82H), 8.13 (minor rotamer, d, *J* = 12.1 Hz, 0.18H), 7.19-7.07 (m, 2H), 6.71-6.65 (m, 2H), 5.90 (br s, 1H), 4.35 (major rotamer, d, *J* = 5.7 Hz, 1.64H), 4.27 (minor rotamer, d, *J* = 6.4 Hz, 0.36H), 2.94 (minor rotamer, s, 1.08H), 2.93 (major rotamer, s, 4.92H).

^{13}C NMR (126 MHz, $CDCl_3$) major rotamer: δ 160.9, 150.2, 129.1, 125.2, 112.7, 45.3, 40.6; minor rotamer: δ 164.5, 150.3, 128.1, 124.9, 45.2, 41.8.

HRMS (ESI): m/z $[M+H]^+$ calcd for $C_{10}H_{15}ON_2$ 179.1179, found 179.1172.

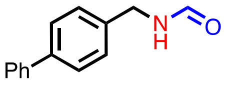

13

N-([1,1'-biphenyl]-4-ylmethyl)formamide[6]**:** ^{1}H NMR (500 MHz, $CDCl_3$) δ 8.25 (major rotamer, s, 0.85H), 8.17 (minor rotamer, d, *J* = 11.9 Hz, 0.15H), 7.57 (d, J = 4.7 Hz, 4H), 7.44 (t, J = 7.6 Hz, 2H), 7.35 (d, J = 8.3 Hz, 3H), 6.33 (br s, 1H), 4.50 (major rotamer, d, *J* = 5.9 Hz, 1.70H), 4.42 (minor rotamer, d, *J* = 6.3 Hz, 0.30H).

^{13}C NMR (126 MHz, $CDCl_3$) major rotamer is given; δ 161.2, 140.6, 140.4, 136.7, 128.8, 128.2, 127.5, 127.4, 127.1, 41.7.

HRMS (ESI): m/z $[M+Na]^+$ calcd for $C_{14}H_{13}ONNa$ 234.0889, found 234.0881.

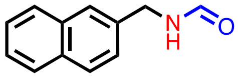

14

N-(naphthalen-2-ylmethyl)formamide[6]**:** ^{1}H NMR (500 MHz, $CDCl_3$) δ 7.86 (major rotamer, s, 0.88H), 7.74 (minor rotamer, d, *J* = 12.0 Hz, 0.12H), 7.54-7.39 (m, 3H), 7.30 (s, 1H), 7.21-7.10 (m, 2H), 6.99 (dd, *J* = 8.4, 1.8 Hz, 1H), 6.32 (major rotamer, br s, 0.88H), 6.09 (minor rotamer, br s, 0.12H), 4.19 (major rotamer, d, *J* = 6.0 Hz, 1.76H), 4.02 (minor rotamer, d, *J* = 6.5 Hz, 0.24H).

^{13}C NMR (126 MHz, $CDCl_3$) major rotamer is given; δ 161.3, 135.1, 133.2, 132.6, 128.4, 127.7, 127.6, 126.3, 126.2, 125.9, 125.7, 42.1.

HRMS (ESI): m/z $[M+H]^+$ calcd for $C_{12}H_{12}ON$ 186.0913, found 186.0907.

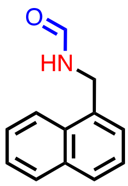

15

N-(napthalen-1-ylmethyl)formamide[6]**:** ^{1}H NMR (500 MHz, $CDCl_3$) δ 8.18 (major rotamer, s, 0.9H), 8.17 (minor rotamer, s, 0.1H), 7.97 (d, *J* = 8.4 Hz, 1H), 7.91-7.85 (m, 1H), 7.80 (dt, *J* = 7.2, 3.6 Hz, 1H), 7.59- 7.48 (m, 2H), 7.46-7.36 (m, 2H), 6.00 (br s, 1H), 4.88 (major rotamer, d, *J* = 5.5 Hz, 1.8H), 4.80 (minor rotamer, d, *J* = 6.2 Hz, 0.20H).

^{13}C NMR (126 MHz, $CDCl_3$) major rotamer is given; δ 160.9, 133.9, 132.9, 131.3, 128.9, 128.8, 126.9, 126.8, 126.1, 125.4, 123.4, 40.3.

HRMS (ESI): m/z $[M+Na]^+$ calcd for $C_{12}H_{11}ONNa$ 208.0733, found 208.0727.

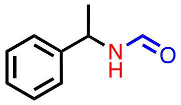

21

N-(1-phenylethyl)formamide[4]: ^{1}H NMR (500 MHz, $CDCl_3$) δ 8.05 (major rotamer, s, 0.80H), 8.03 (minor rotamer, s, 0.20H), 7.54-6.91 (m, 5H), 6.37 (minor rotamer, br s, 0.20H), 6.14 (major rotamer, br s, 0.80H), 5.11 (major rotamer, m, *J* = 7.3 Hz, 0.80H), 4.59 (minor rotamer, m, *J* = 7.2 Hz, 0.20H), 1.47 (minor rotamer, d, *J* = 6.9 Hz, 0.60H), 1.43 (major rotamer, d, *J* = 7.0 Hz, 2.40H).

^{13}C NMR (126 MHz, $CDCl_3$) major rotamer: δ 160.4, 142.7, 128.7, 127.5, 126.2, 47.6, 21.8; minor rotamer: δ 164.3, 142.9, 128.9, 127.8, 125.8, 51.8, 23.6.

HRMS (ESI): m/z $[M+H]^+$ calcd for $C_9H_{12}NO$ 150.0913, found 150.0908.

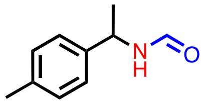

22

N-(1-(p-tolyl)ethyl)formamide: ^{1}H NMR (500 MHz, $CDCl_3$) δ 8.13 (major rotamer, s, 0.81H), 8.10 (minor rotamer, s, 0.19H), 7.22-7.19 (m, 2H), 7.17-7.12 (m, 2H), 6.26 (minor rotamer, br s, 0.19H), 6.09 (major rotamer, br s, 0.81H), 5.16 (major rotamer, m, *J* = 7.2 Hz, 0.81H), 4.67-4.58 (minor rotamer, m, 0.19H), 2.34 (minor rotamer, s, 0.57H), 2.32 (major rotamer, s, 2.43H), 1.53 (minor rotamer, d, *J* = 6.9 Hz, 0.57H), 1.49 (major rotamer, d, *J* = 6.9 Hz, 2.43H).

^{13}C NMR (126 MHz, $CDCl_3$) major rotamer is given; δ 160.32, 139.67, 137.23, 129.40, 126.10, 47.36, 21.75, 21.07.

HRMS (ESI): m/z $[M+H]^+$ calcd for $C_{10}H_{14}ON$ 164.1070, found 164.1065.

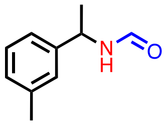

23

N-(1-(m-tolyl)ethyl)formamide: ^{1}H NMR (500 MHz, $CDCl_3$) δ 8.16 (major rotamer, s, 0.80H), 8.13 (minor rotamer, s, 0.20H), 7.23 (t, *J* = 7.6 Hz, 1H), 7.15-7.04 (m, 3H), 6.01 (minor rotamer, br s, 0.20H), 5.86 (major rotamer, br s, 0.80H), 5.18 (major rotamer, p, *J* = 7.2 Hz, 0.80H), 4.65 (minor rotamer, dt, *J* = 13.8, 7.1 Hz, 0.20H), 2.36 (minor rotamer, s, 0.60H), 2.35 (major rotamer, s, 2.40H), 1.55 (minor rotamer, d, *J* = 6.9 Hz, 0.60H), 1.51 (major rotamer, d, *J* = 6.9 Hz, 2.40H).

^{13}C NMR (126 MHz, $CDCl_3$) major rotamer is given; δ 160.2, 142.5, 138.5, 128.7, 128.3, 123.0, 123.1, 47.6, 21.8, 21.5.

HRMS (ESI): m/z $[M+Na]^+$ calcd for $C_{10}H_{13}ONNa$ 186.0889, found 186.0882.

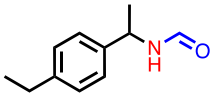

24

N-[1-(4-ethylphenyl)ethyl]formamide: ^{1}H NMR (500 MHz, $CDCl_3$) δ 8.13 (major rotamer, s, 0.79H), 8.12 (minor rotamer, s, 0.21H), 7.25-7.22 (m, 2H), 7.20-7.15 (m, 2H), 6.20 (minor rotamer, br s, 0.21H), 6.02 (major rotamer, br s, 0.79H), 5.18 (major rotamer, p, *J* = 7.2 Hz, 0.79H), 4.70-4.59 (minor rotamer, m, 0.21H), 2.69-2.57 (m, 2H), 1.54 (minor rotamer, d, *J* = 6.9 Hz, 0.63H), 1.50 (major rotamer, d, *J* = 6.9 Hz, 2.37H), 1.23 (td, *J* = 7.6, 4.5 Hz, 3H).

^{13}C NMR (126 MHz, $CDCl_3$): major rotamer: δ 160.3, 143.6, 139.8, 128.2, 126.2, 47.4, 28.5, 21.7, 15.6; minor rotamer: δ 164.2, 143.9, 140.1, 128.4, 125.8, 51.5, 28.5, 23.6, 15.6.

HRMS (ESI): m/z $[M+H]^+$ calcd for $C_{11}H_{16}ON$ 178.1226, found 178.1220.

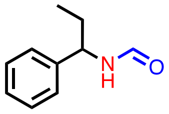

25

N-(1-phenylethyl)formamide: ^{1}H NMR (500 MHz, $CDCl_3$) δ 8.14 (major rotamer, s, 0.78H), 8.07 (minor rotamer, d, *J* = 11.9 Hz, 0.22H), 7.34-7.27 (m, 2H), 7.25-7.18 (m, 3H), 6.39 (minor rotamer, br s, 0.22H), 6.07 (major rotamer, br s, 0.78H), 4.91 (major rotamer, q, *J* = 7.6 Hz, 0.78H), 4.32 (minor rotamer, td, *J* = 8.4, 6.2 Hz, 0.22H), 1.81 (dqd, *J* = 10.2, 7.1, 3.9 Hz, 2H), 0.88 (dt, *J* = 19.1, 7.4 Hz, 3H).

^{13}C NMR (126 MHz, $CDCl_3$) major rotamer: δ 160.6, 141.6, 128.7, 127.5, 126.6, 53.8, 29.1, 10.7; minor rotamer: δ 164.6, 141.8, 128.9, 127.8, 126.2, 58.2, 30.3.

HRMS (ESI): m/z $[M+Na]^+$ calcd for $C_{10}H_{13}ONNa$ 186.0889, found 186.0885.

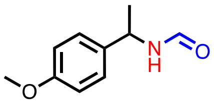

26

N-(1-(4-methoxyphenyl)-ethyl)formamide[7]**:** ^{1}H NMR (500 MHz, $CDCl_3$) δ 8.13 (major rotamer, s, 0.80H), 8.11 (minor rotamer, s, 0.20H), 7.24 (d, *J* = 8.6 Hz, 2H), 6.86 (d, *J* = 8.7 Hz, 2H), 6.10 (minor rotamer, br s, 0.20H), 5.92 (major rotamer, br s, 0.80H), 5.13-5.19 (major rotamer, m, 0.80H), 4.61-4.66 (minor rotamer, m, 0.20H), 3.80 (minor rotamer, s, 0.60H), 3.78 (major rotamer, s, 2.40H), 1.53 (minor rotamer, d, *J* = 6.9 Hz, 0.60H), 1.49 (major rotamer, d, *J* = 6.9 Hz, 2.40H).

^{13}C NMR (126 MHz, $CDCl_3$) major rotamer: δ 160.3, 159.0, 134.7, 127.4, 114.1, 55.3, 47.1, 21.7; minor rotamer: δ 164.1, 159.1, 134.9, 127.1, 114.3, 55.4, 51.1, 23.6.

HRMS (ESI): m/z $[M+H]^+$ calcd for $C_{10}H_{14}O_2N$ 180.1019, found 180.1012.

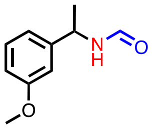

27

N-[1-(3-Methoxyphenyl)ethyl]formamide[8]**:** ^{1}H NMR (500 MHz, $CDCl_3$) δ 8.17 (major rotamer, s, 0.79H), 8.14 (minor rotamer, s, 0.21H), 7.27 (d, *J* = 8.0 Hz, 1H), 6.91 (ddt, J = 7.7, 1.7, 0.8 Hz, 1H), 6.86 (q, *J* = 2.2 Hz, 1H), 6.83-6.79 (m, 1H), 5.96 (minor rotamer, br s, 0.21H), 5.82 (major rotamer, br s, 0.79H), 5.19 (major rotamer, m, 0.79H), 4.66 (minor rotamer, m, 0.21H), 3.81 (minor rotamer, s, 0.63H), 3.80 (major rotamer, s, 2.37H), 1.55 (minor rotamer, d, *J* = 6.9 Hz, 0.63H), 1.51 (major rotamer, d, *J* = 6.9 Hz, 2.37H).

^{13}C NMR (126 MHz, $CDCl_3$) major rotamer: δ 160.3, 160.0, 144.3, 129.9, 118.4, 112.8, 112.3, 55.3, 47.7, 21.8; minor rotamer: δ 164.1, 160.1, 144.5, 130.1, 118.1, 112.9, 111.9, 55.4, 51.6, 23.7.

HRMS (ESI): m/z $[M+H]^+$ calcd for $C_{10}H_{14}O_2N$ 180.1019, found 180.1014.

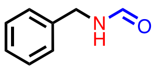
N
H
O

1

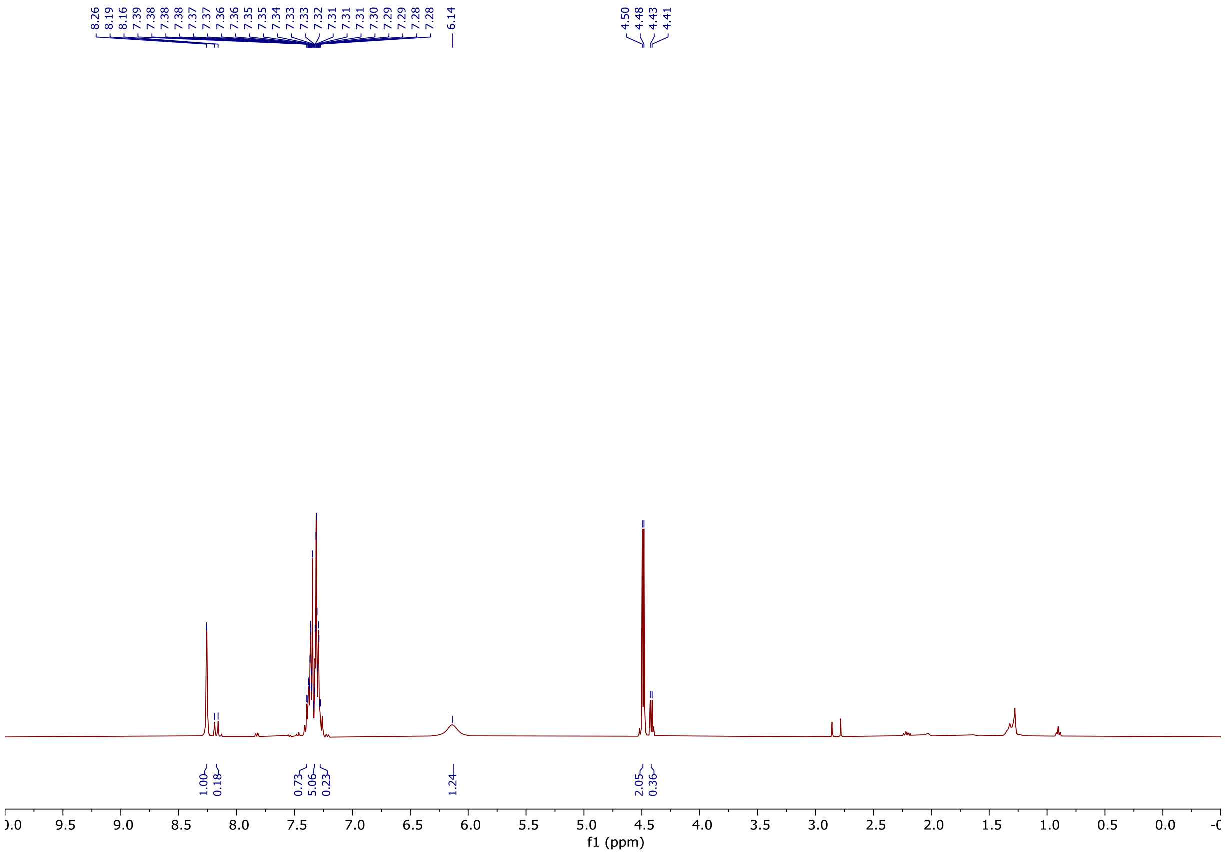
8.26
8.19
8.16
7.39
7.38
7.38
7.38
7.37
7.37
7.36
7.36
7.35
7.35
7.34
7.33
7.33
7.32
7.31
7.31
7.31
7.30
7.29
7.29
7.28
7.28
6.14
4.50
4.48
4.43
4.41
1.00
0.18
0.73
5.06
0.23
1.24
2.05
0.36
f1 (ppm)

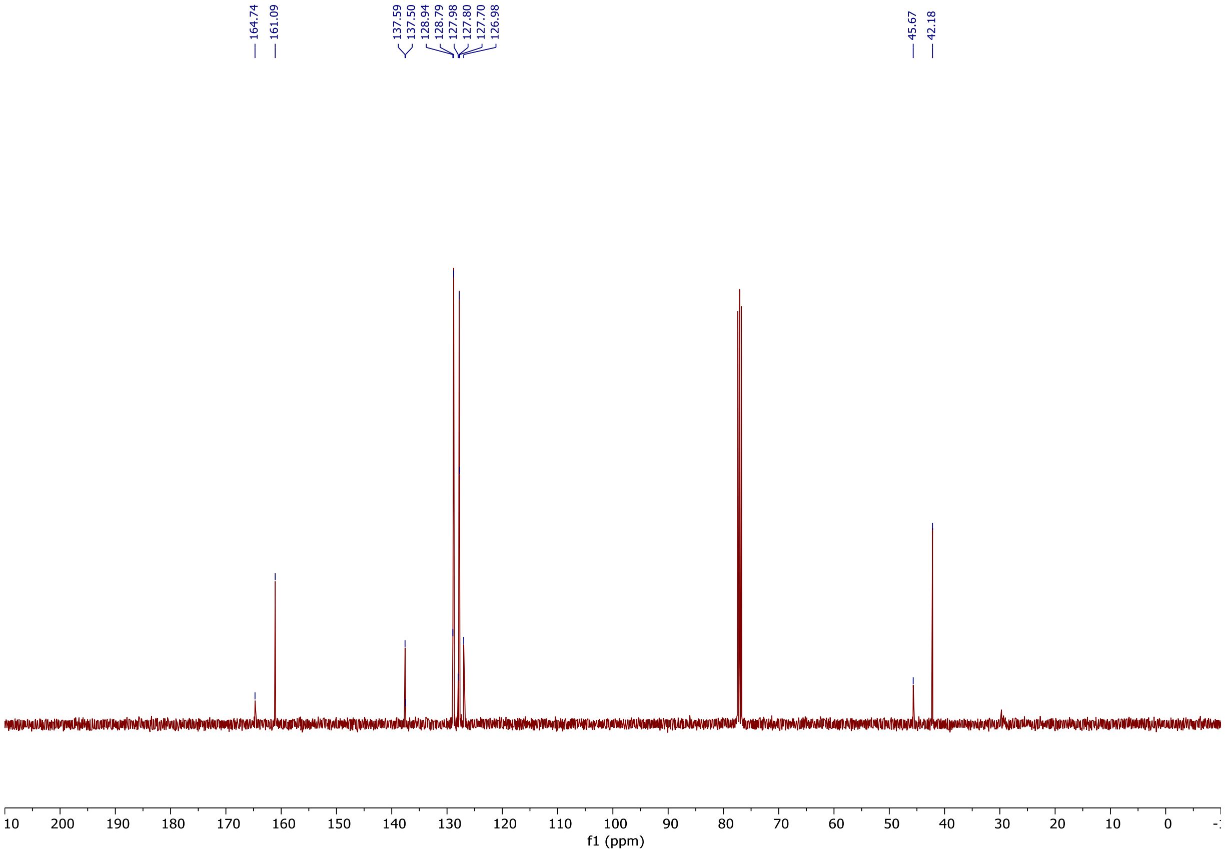
164.74
161.09
137.59
137.50
128.94
128.79
127.98
127.80
127.70
126.98
45.67
42.18
f1 (ppm)

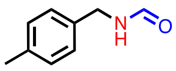
N
H
O

2

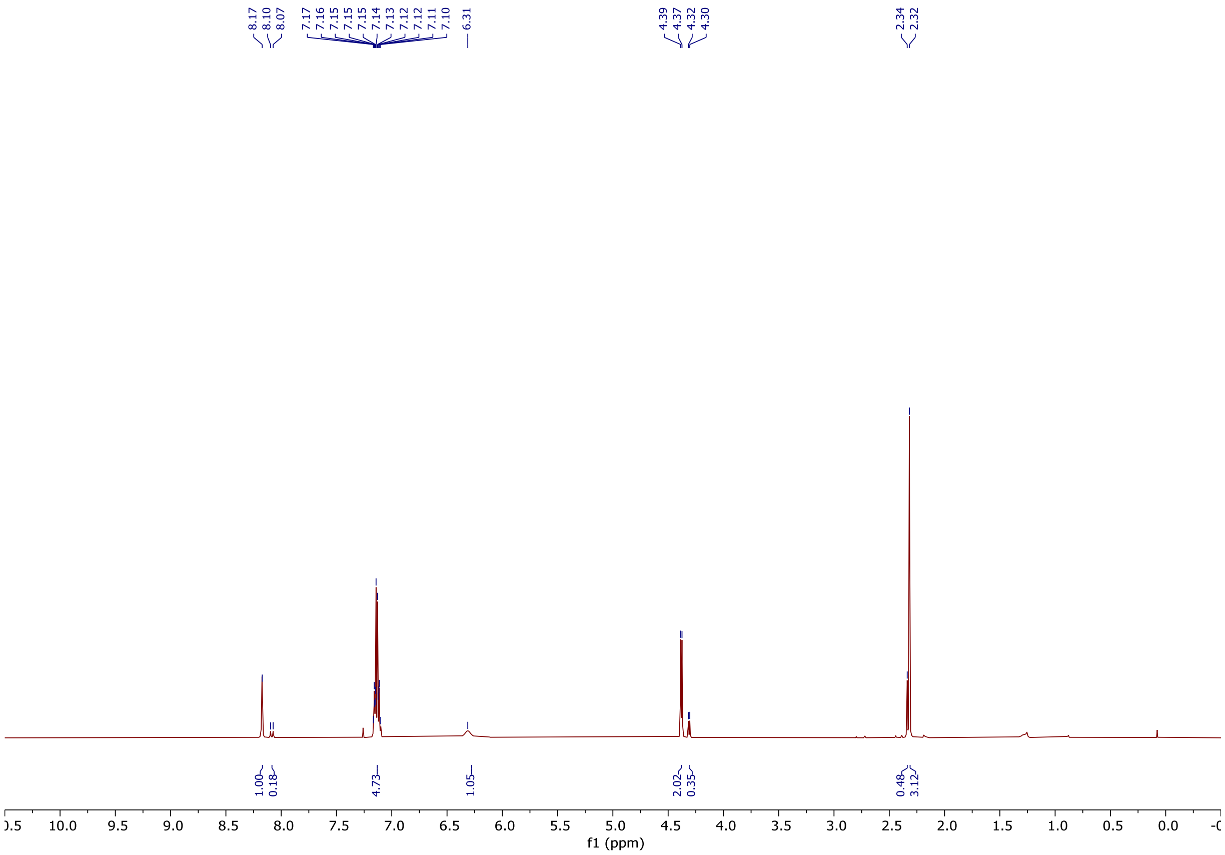
8.17
8.10
8.07
7.17
7.16
7.15
7.15
7.15
7.14
7.13
7.12
7.12
7.11
7.10
6.31
4.39
4.37
4.32
4.30
2.34
2.32
1.00
0.18
4.73
1.05
2.02
0.35
0.48
3.12
f1 (ppm)

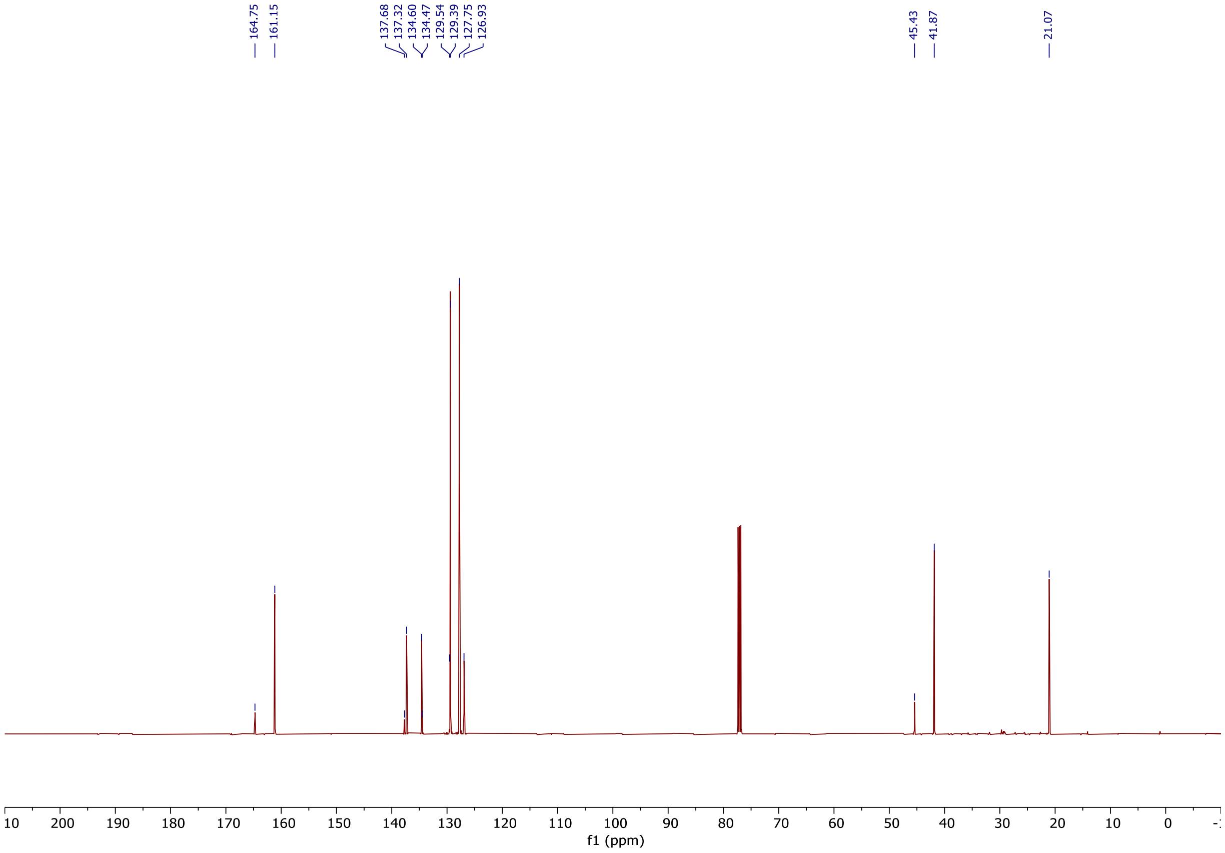
164.75
161.15
137.68
137.32
134.60
134.47
129.54
129.39
127.75
126.93
45.43
41.87
21.07
f1 (ppm)

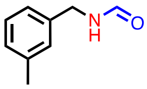
N
H
O

3

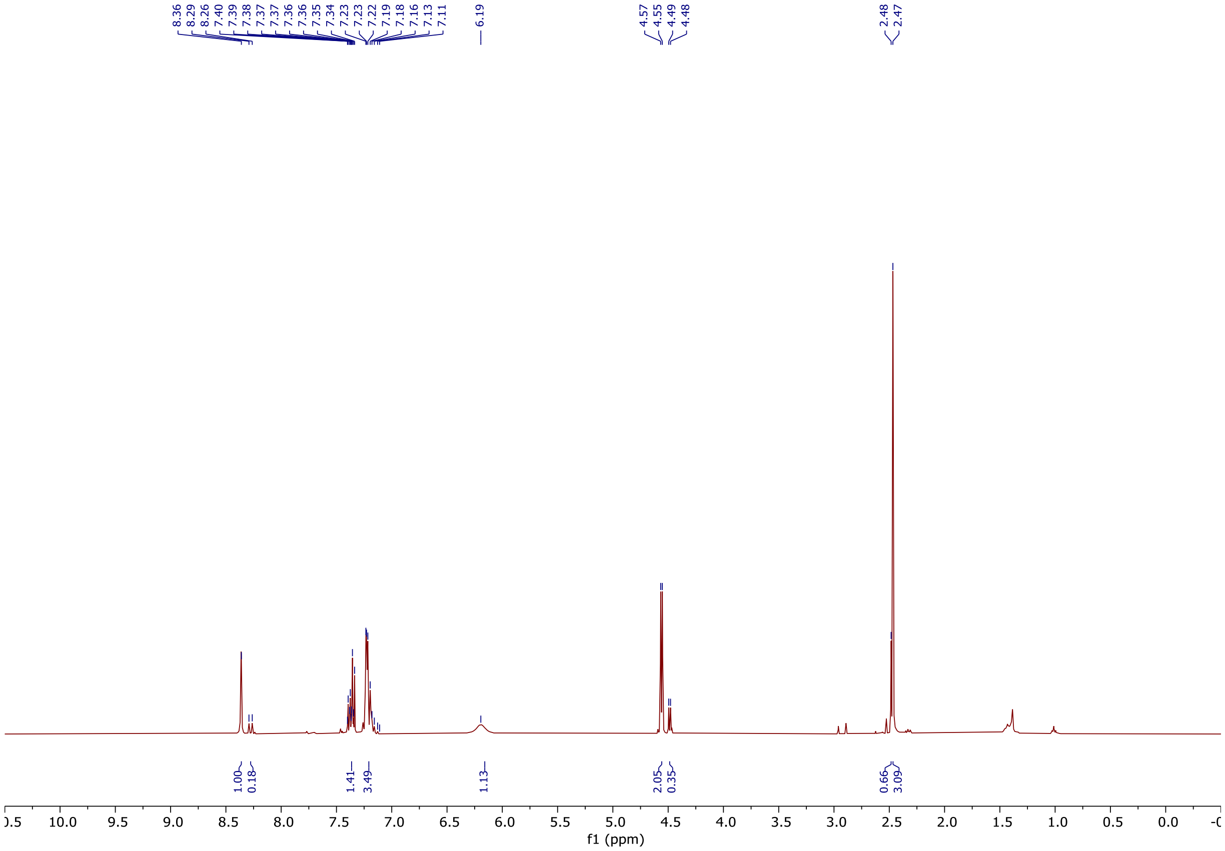
8.36
8.29
8.26
7.40
7.39
7.38
7.37
7.37
7.36
7.36
7.35
7.34
7.23
7.23
7.22
7.19
7.18
7.16
7.13
7.11
6.19
4.57
4.55
4.49
4.48
2.48
2.47
1.00
0.18
1.41
3.49
1.13
2.05
0.35
0.66
3.09
f1 (ppm)

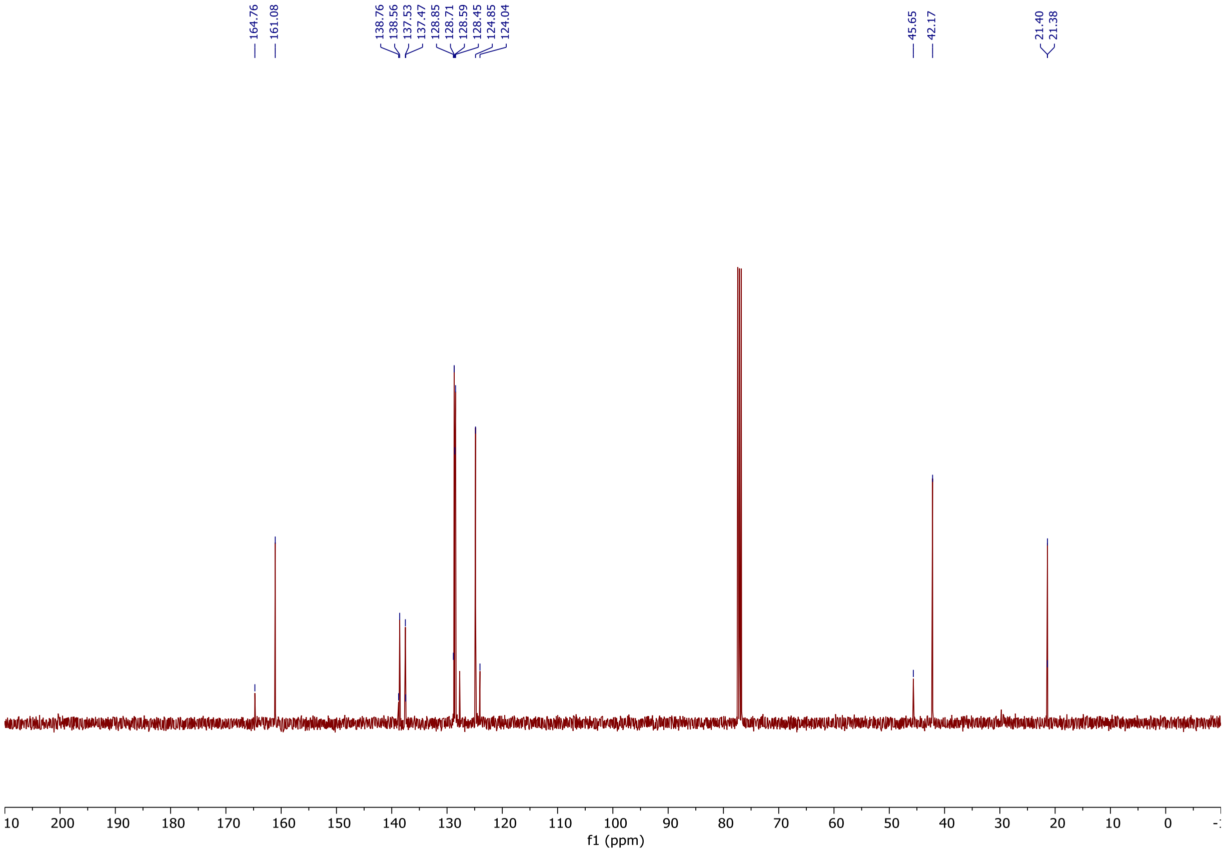
164.76
161.08
138.76
138.56
137.53
137.47
128.85
128.71
128.59
128.45
124.85
124.04
45.65
42.17
21.40
21.38
f1 (ppm)

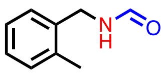
N
H
O

4

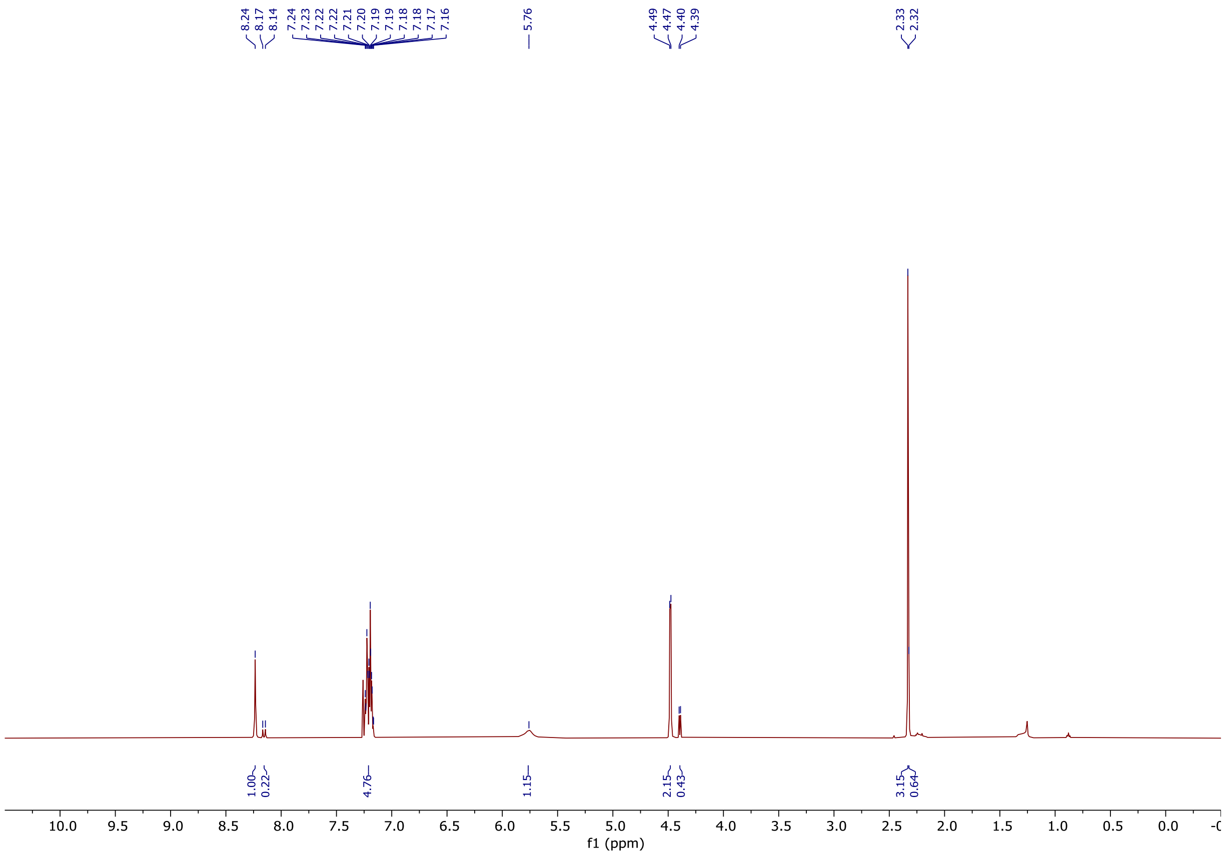
8.24
8.17
8.14
7.24
7.23
7.22
7.22
7.21
7.20
7.19
7.19
7.18
7.18
7.17
7.16
5.76
4.49
4.47
4.40
4.39
2.33
2.32
1.00
0.22
4.76
1.15
2.15
0.43
3.15
0.64
10.0 9.5 9.0 8.5 8.0 7.5 7.0 6.5 6.0 5.5 5.0 4.5 4.0 3.5 3.0 2.5 2.0 1.5 1.0 0.5 0.0
f1 (ppm)

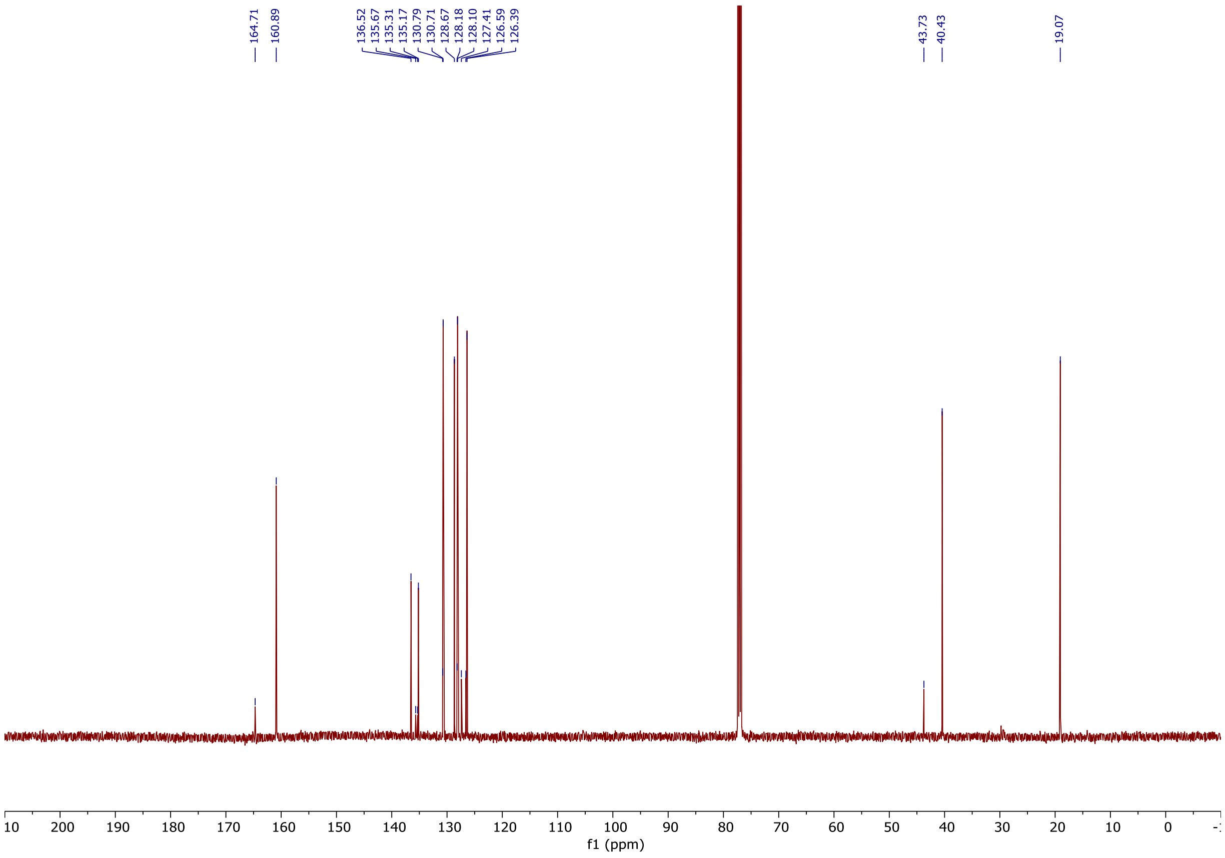
164.71
160.89
136.52
135.67
135.31
135.17
130.79
130.71
128.67
128.18
128.10
127.41
126.59
126.39
43.73
40.43
19.07
200 190 180 170 160 150 140 130 120 110 100 90 80 70 60 50 40 30 20 10 0
f1 (ppm)

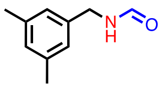
N
H
O

5

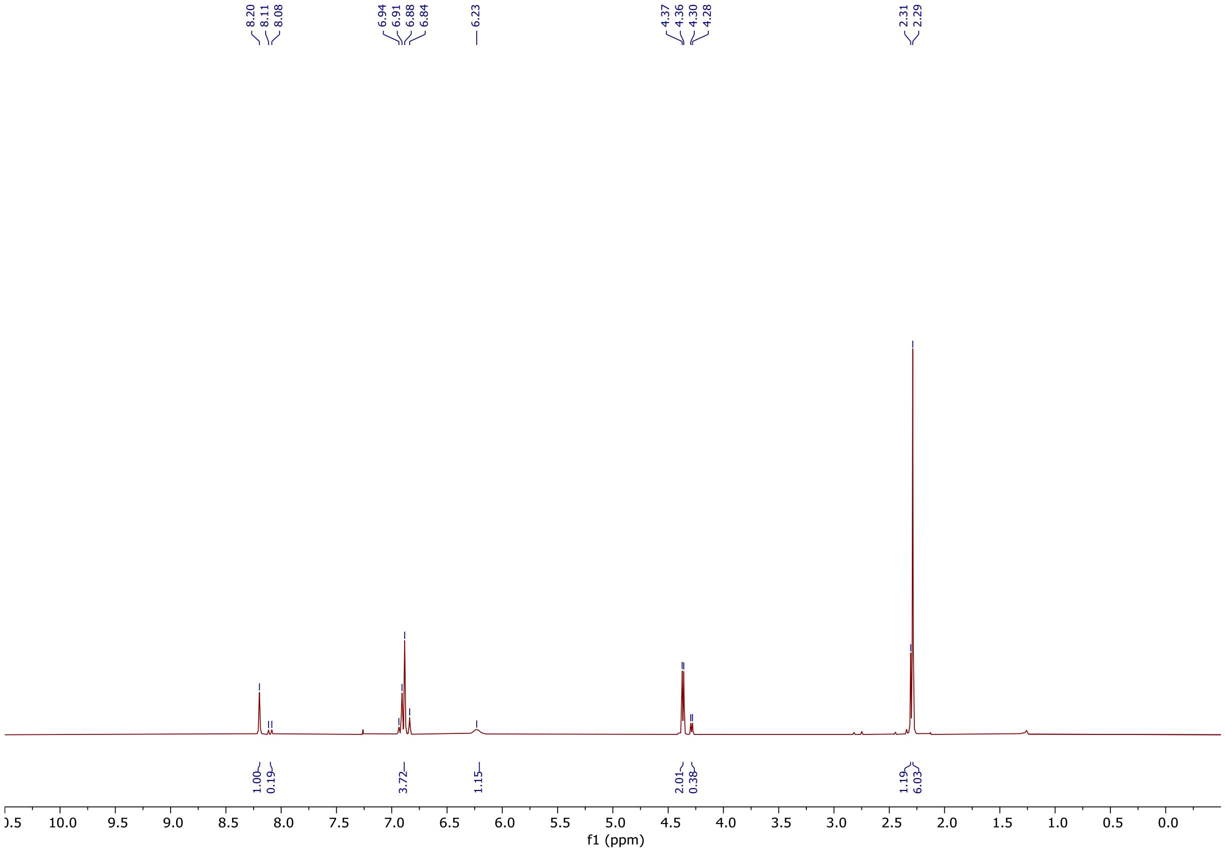
8.20
8.11
8.08
6.94
6.91
6.88
6.84
6.23
4.37
4.36
4.30
4.28
2.31
2.29
1.00
0.19
3.72
1.15
2.01
0.38
1.19
6.03
f1 (ppm)

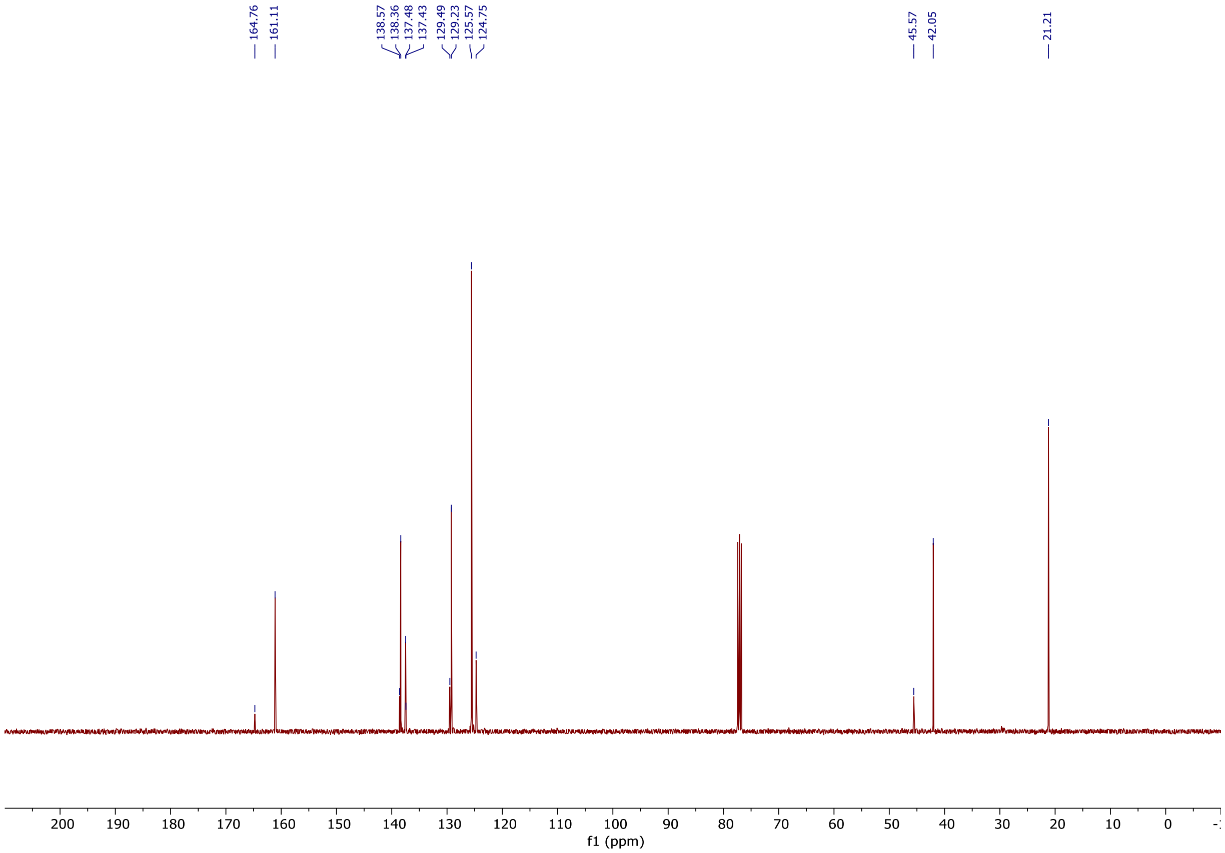
164.76
161.11
138.57
138.36
137.48
137.43
129.49
129.23
125.57
124.75
45.57
42.05
21.21
f1 (ppm)

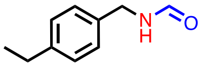
N
H
O

6

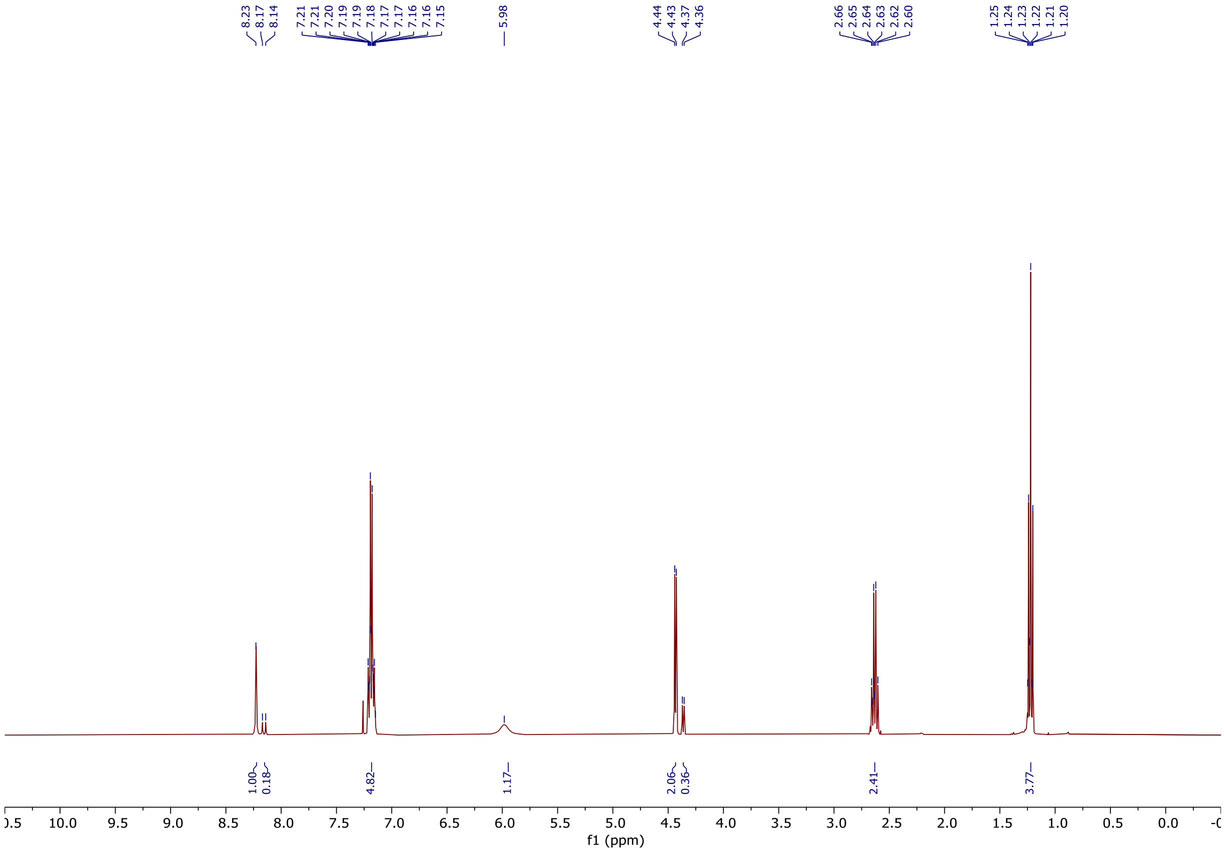
8.23
8.17
8.14
7.21
7.21
7.20
7.19
7.19
7.18
7.17
7.17
7.16
7.16
7.15
5.98
4.44
4.43
4.37
4.36
2.66
2.65
2.64
2.63
2.62
2.60
1.25
1.24
1.23
1.22
1.21
1.20
1.00
0.18
4.82
1.17
2.06
0.36
2.41
3.77
f1 (ppm)

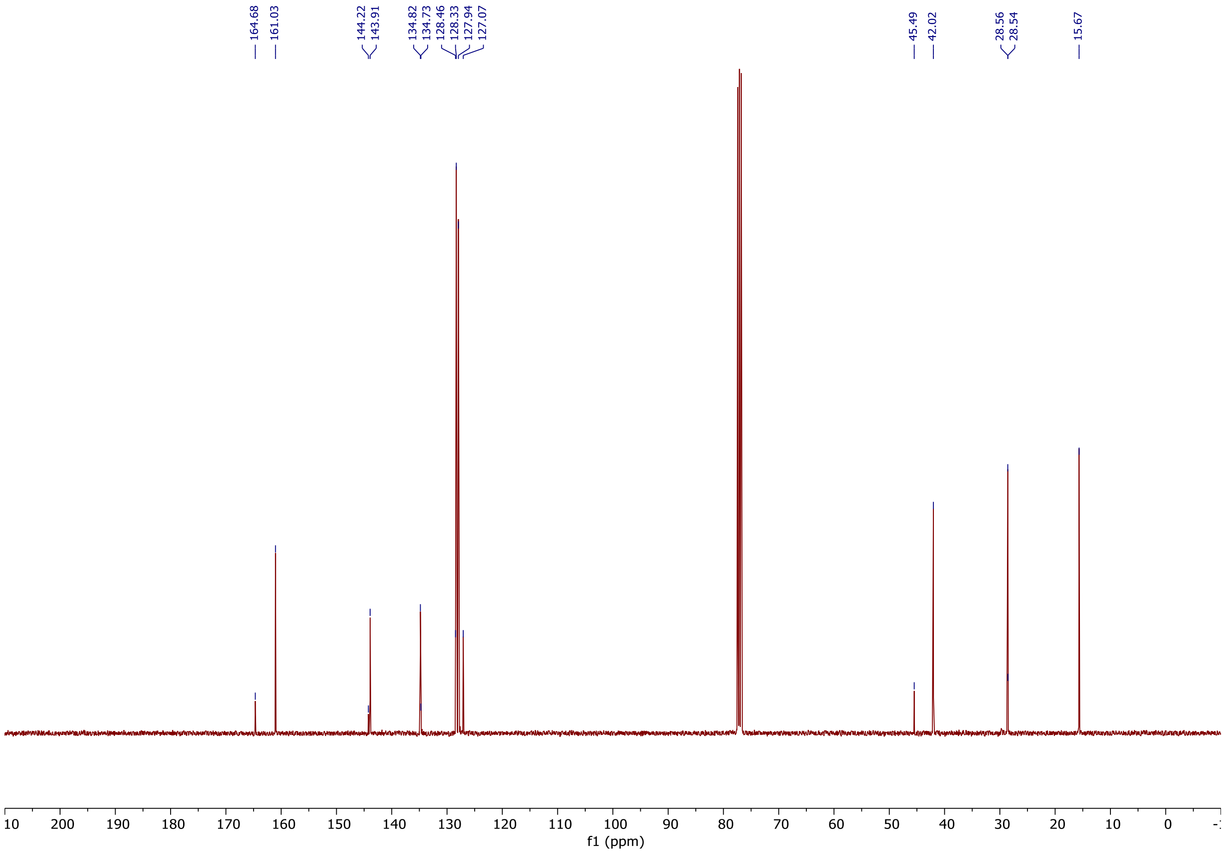
164.68
161.03
144.22
143.91
134.82
134.73
128.46
128.33
127.94
127.07
45.49
42.02
28.56
28.54
15.67
f1 (ppm)

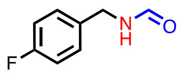
F
N
H
O

7

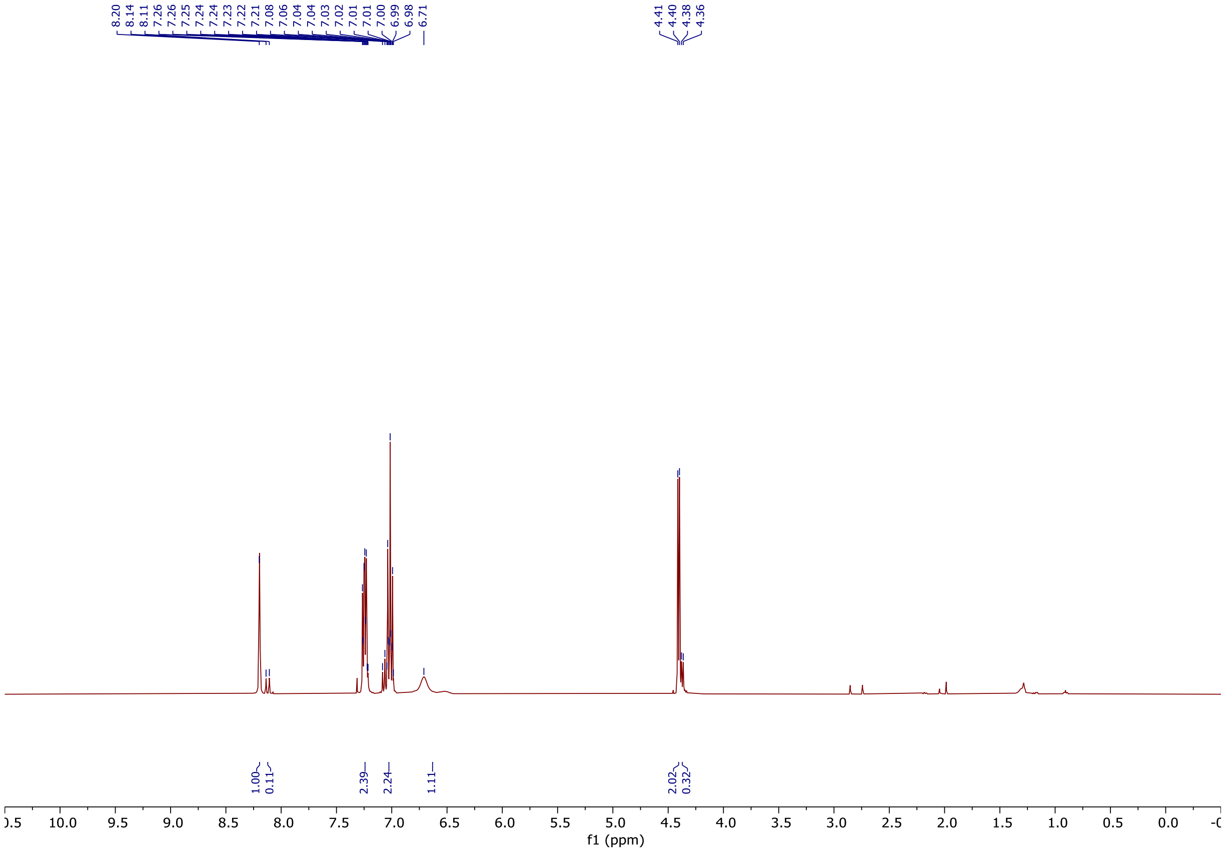
8.20
8.14
8.11
7.26
7.26
7.25
7.24
7.24
7.23
7.22
7.21
7.08
7.06
7.04
7.04
7.03
7.02
7.01
7.00
6.99
6.98
6.71
4.41
4.40
4.38
4.36
1.00
0.11
2.39
2.24
1.11
2.02
0.32
f1 (ppm)

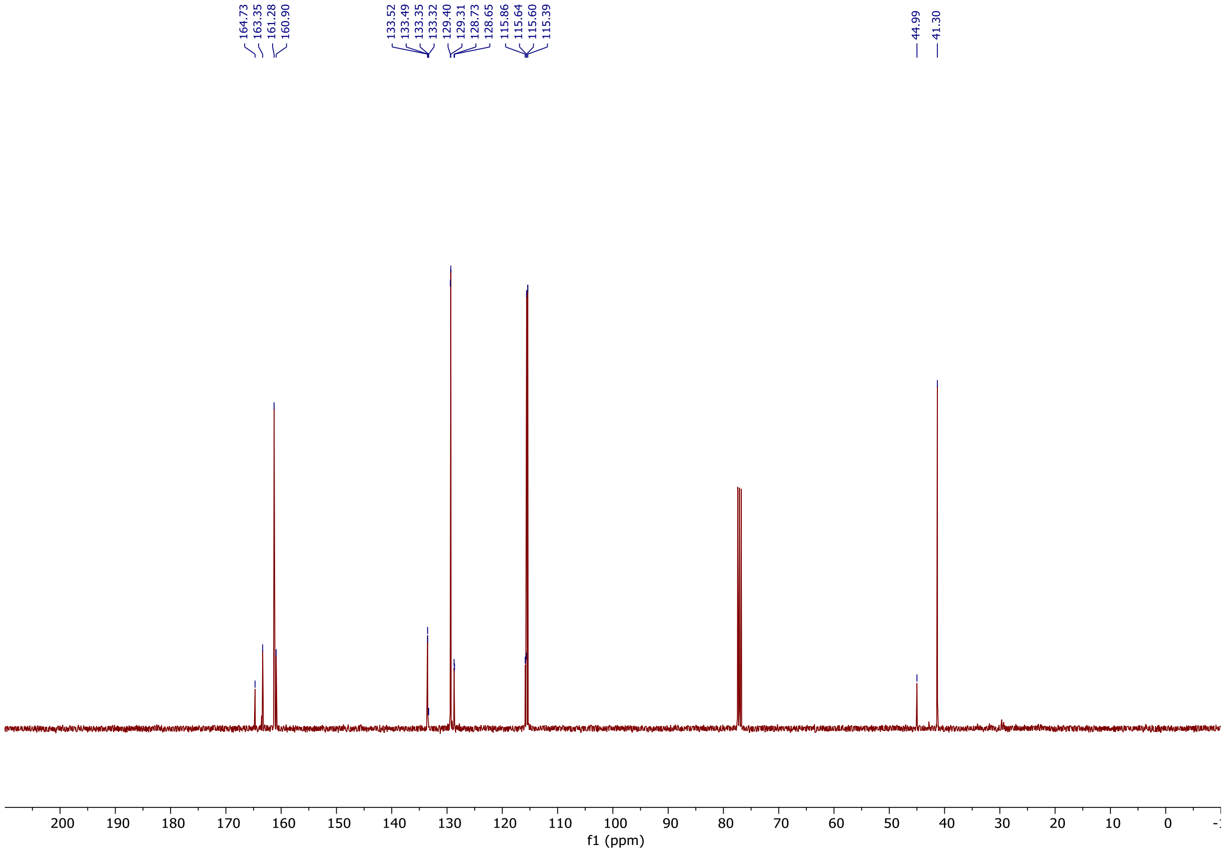
164.73
163.35
161.28
160.90
133.52
133.49
133.35
133.32
129.40
129.31
128.73
128.65
115.86
115.64
115.60
115.39
44.99
41.30
f1 (ppm)

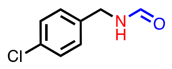

Cl
N
H
O

8

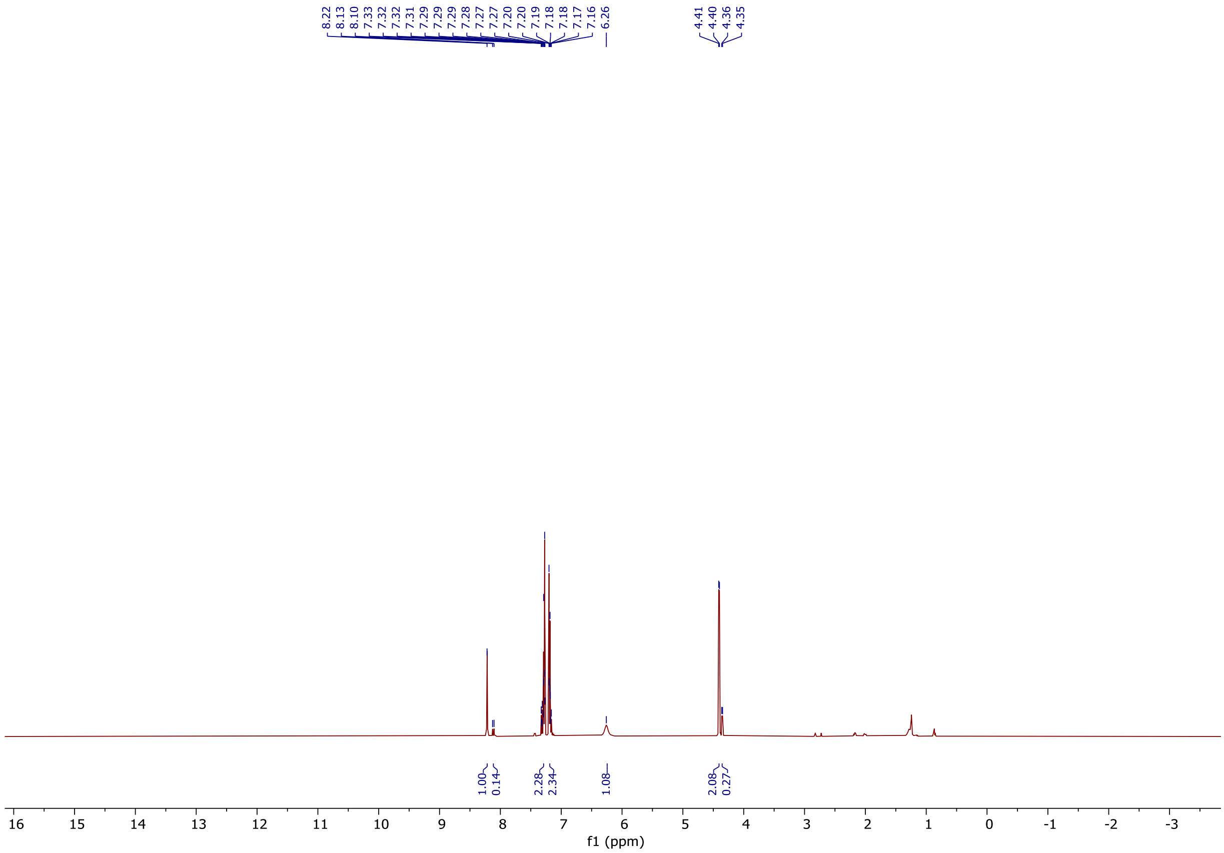

8.22 8.13 8.10 7.33 7.32 7.32 7.31 7.29 7.29 7.29 7.28 7.27 7.27 7.20 7.20 7.19 7.18 7.18 7.17 7.16 6.26
4.41 4.40 4.36 4.35
1.00 0.14 2.28 2.34 1.08 2.08 0.27
f1 (ppm)

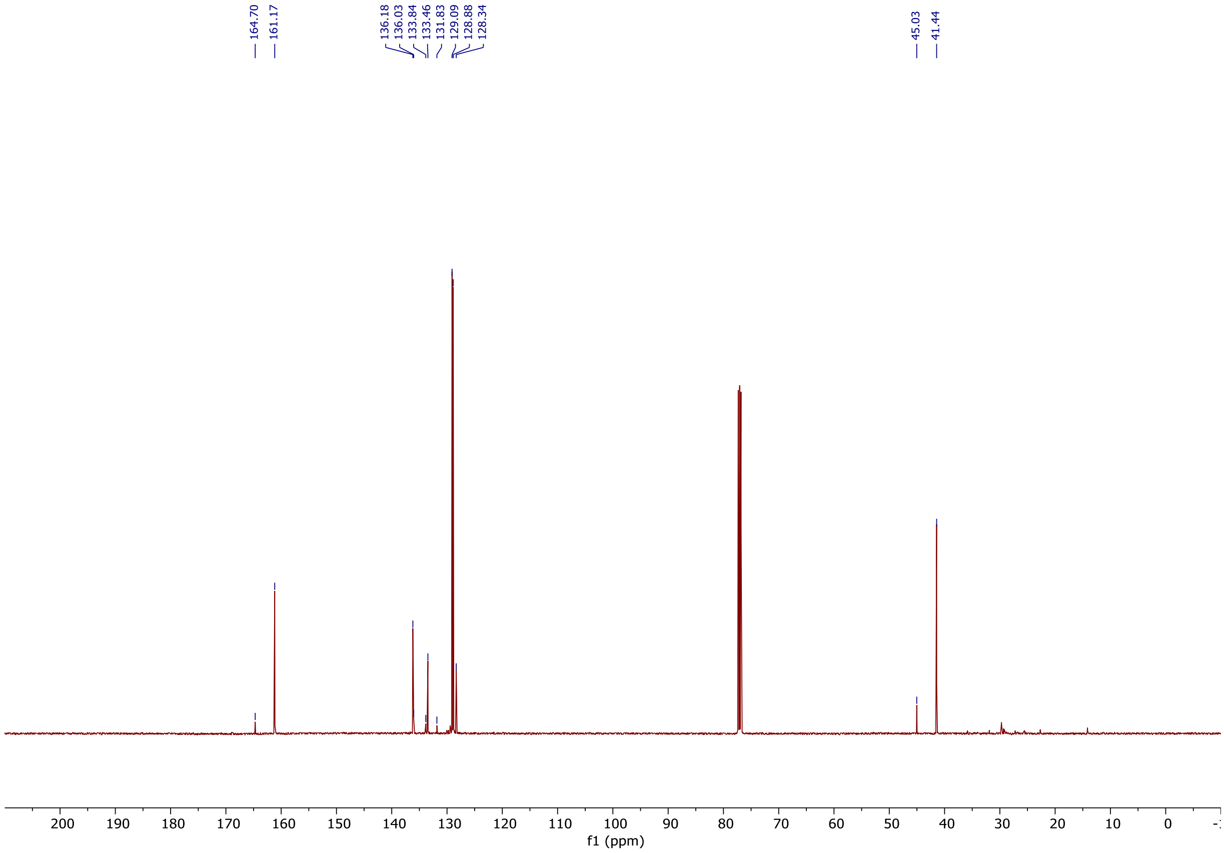

164.70 161.17
136.18 136.03 133.84 133.46 131.83 129.09 128.88 128.34
45.03 41.44
f1 (ppm)

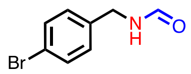

9

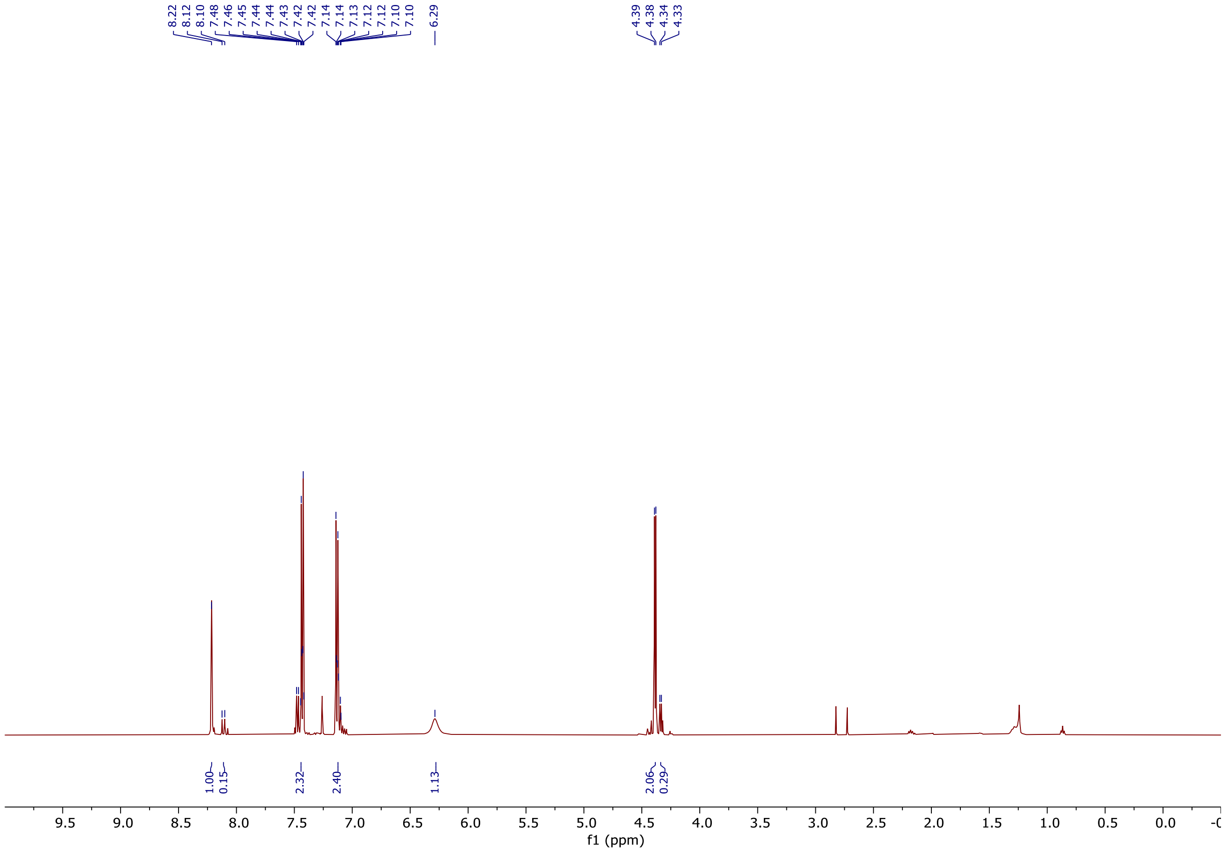
8.22
8.12
8.10
7.48
7.46
7.45
7.44
7.44
7.43
7.42
7.42
7.14
7.14
7.13
7.12
7.12
7.10
7.10
6.29
4.39
4.38
4.34
4.33
1.00
0.15
2.32
2.40
1.13
2.06
0.29
9.5 9.0 8.5 8.0 7.5 7.0 6.5 6.0 5.5 5.0 4.5 4.0 3.5 3.0 2.5 2.0 1.5 1.0 0.5 0.0
f1 (ppm)

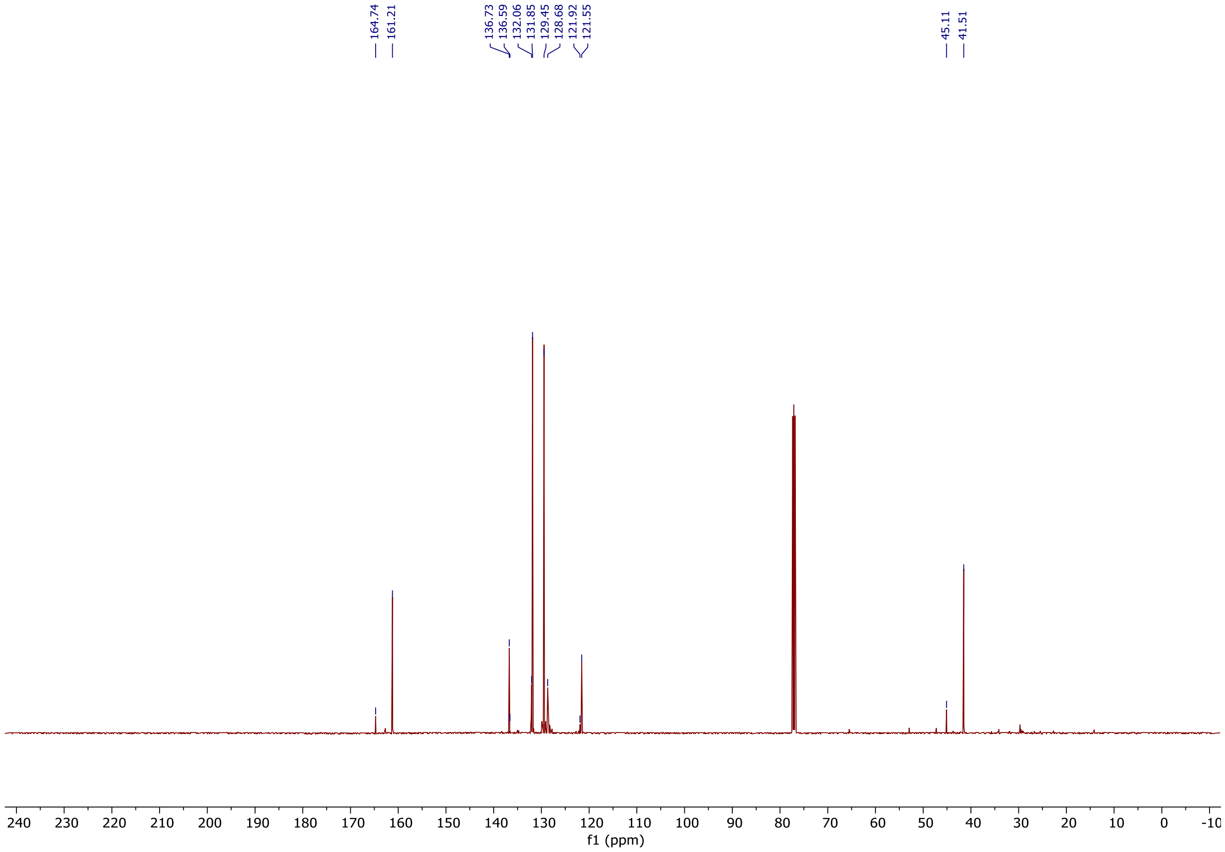
164.74
161.21
136.73
136.59
132.06
131.85
129.45
128.68
121.92
121.55
45.11
41.51
240 230 220 210 200 190 180 170 160 150 140 130 120 110 100 90 80 70 60 50 40 30 20 10 0 -10
f1 (ppm)

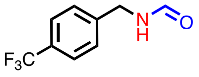
N
H
O
F3C

10

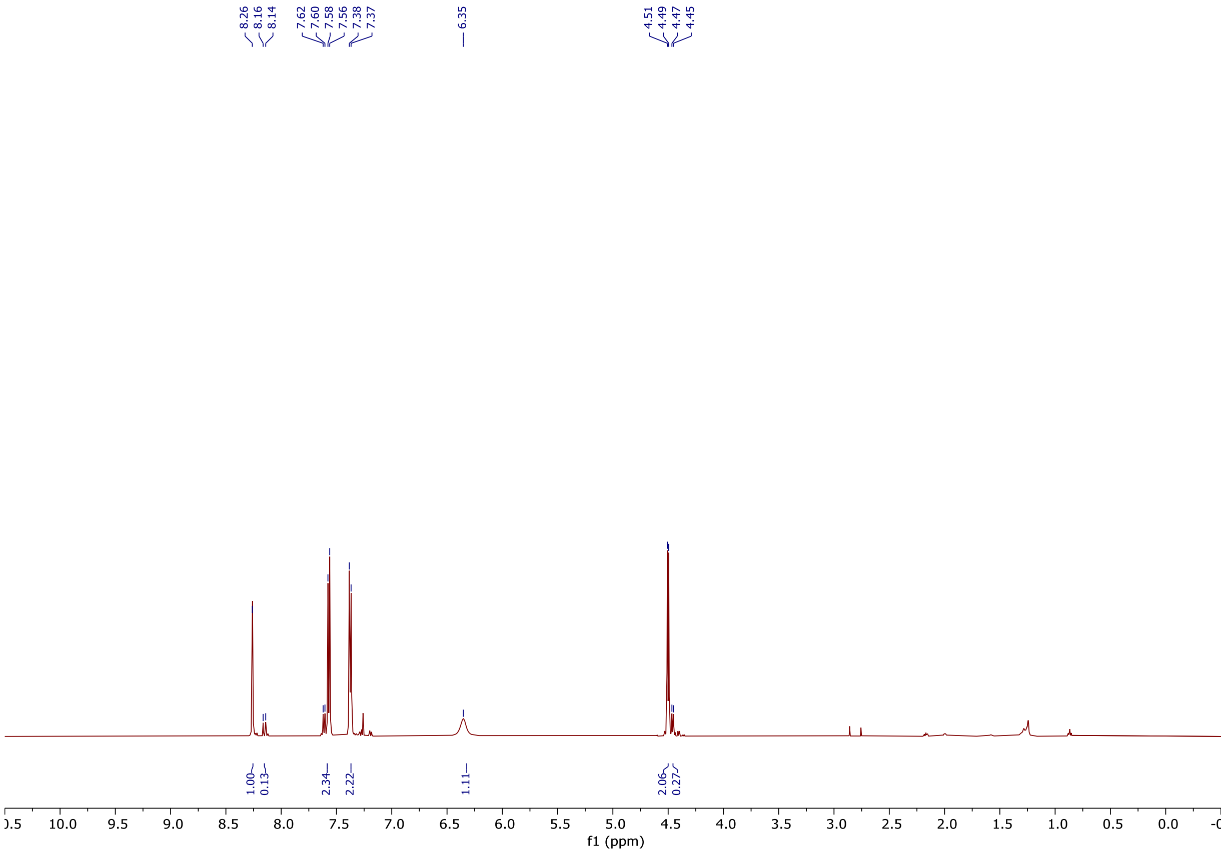
8.26
8.16
8.14
7.62
7.60
7.58
7.56
7.38
7.37
6.35
4.51
4.49
4.47
4.45
1.00
0.13
2.34
2.22
1.11
2.06
0.27
f1 (ppm)

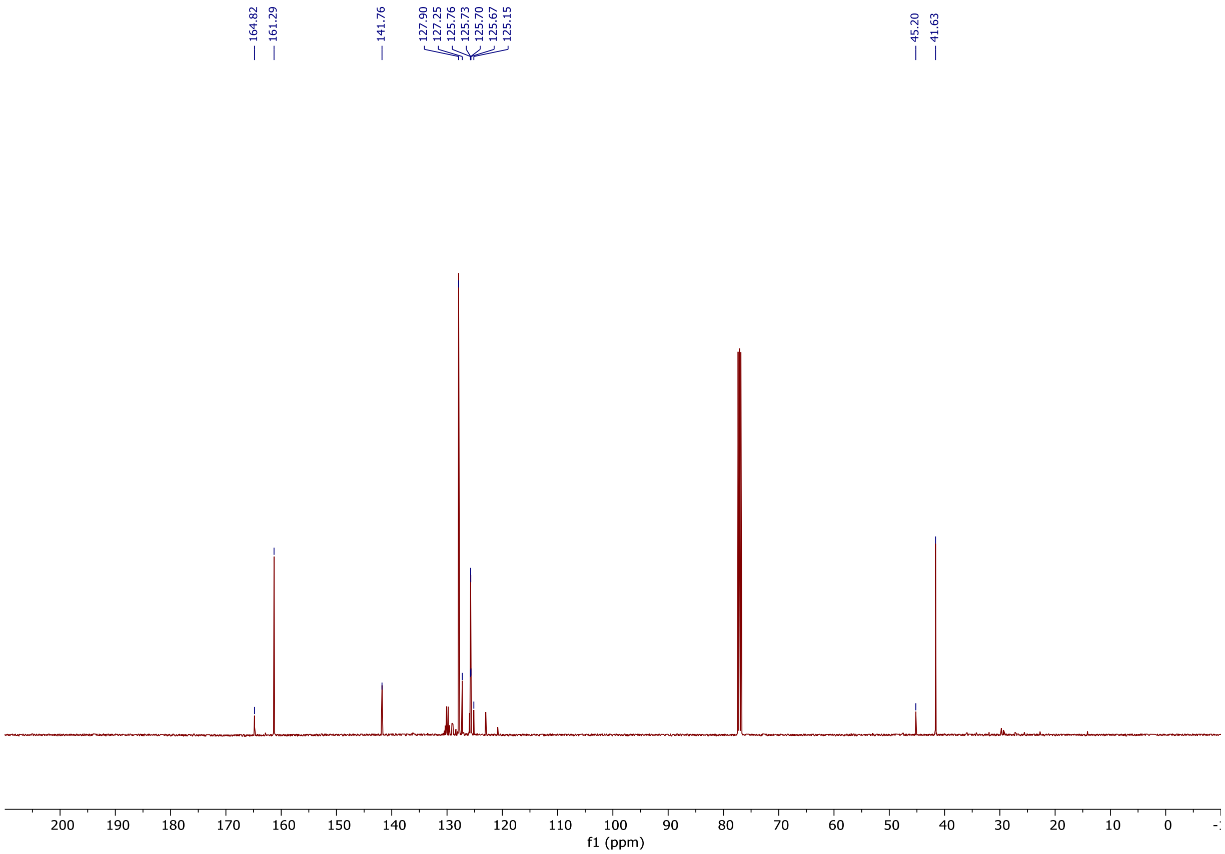
164.82
161.29
141.76
127.90
127.25
125.76
125.73
125.70
125.67
125.15
45.20
41.63
f1 (ppm)

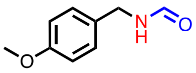
N
H
O

11

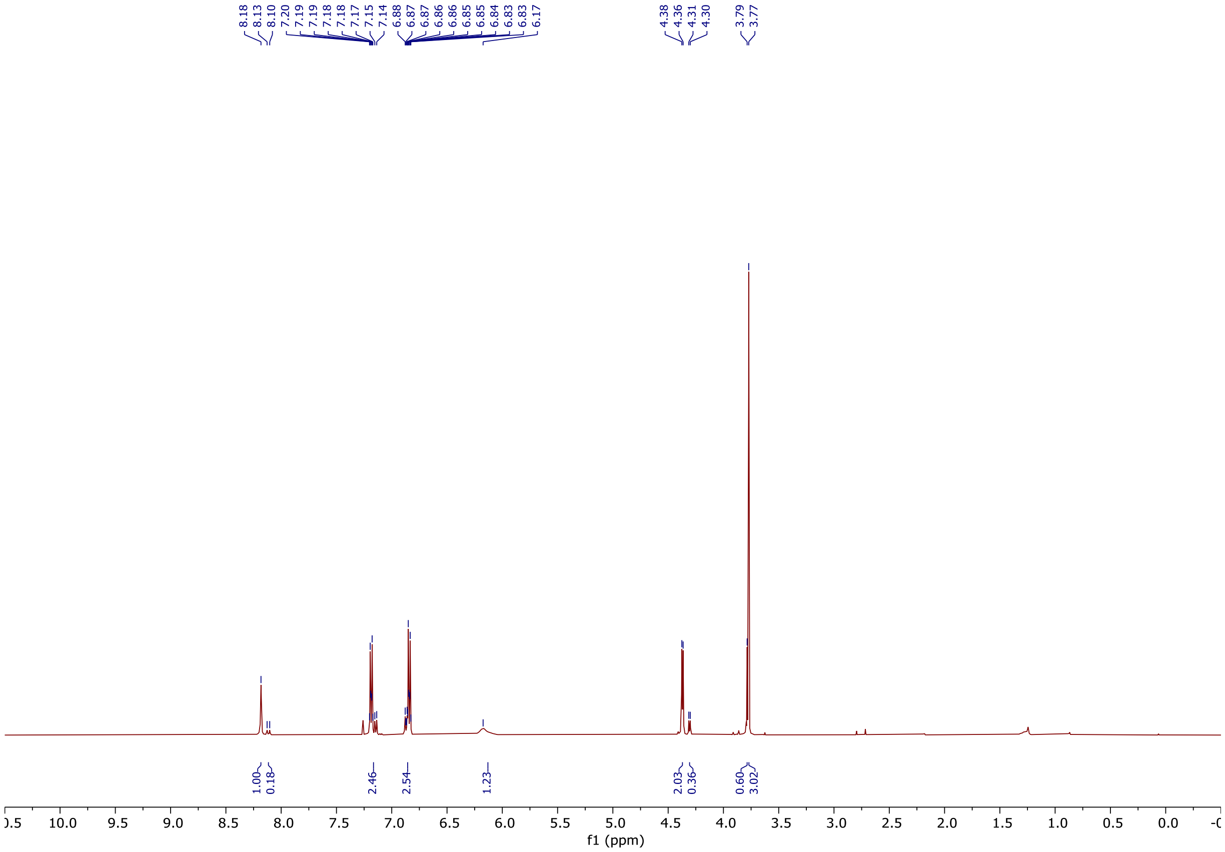
8.18
8.13
8.10
7.20
7.19
7.19
7.18
7.18
7.17
7.15
7.14
6.88
6.87
6.87
6.86
6.86
6.85
6.85
6.84
6.83
6.83
6.17
4.38
4.36
4.31
4.30
3.79
3.77
1.00
0.18
2.46
2.54
1.23
2.03
0.36
0.60
3.02
f1 (ppm)

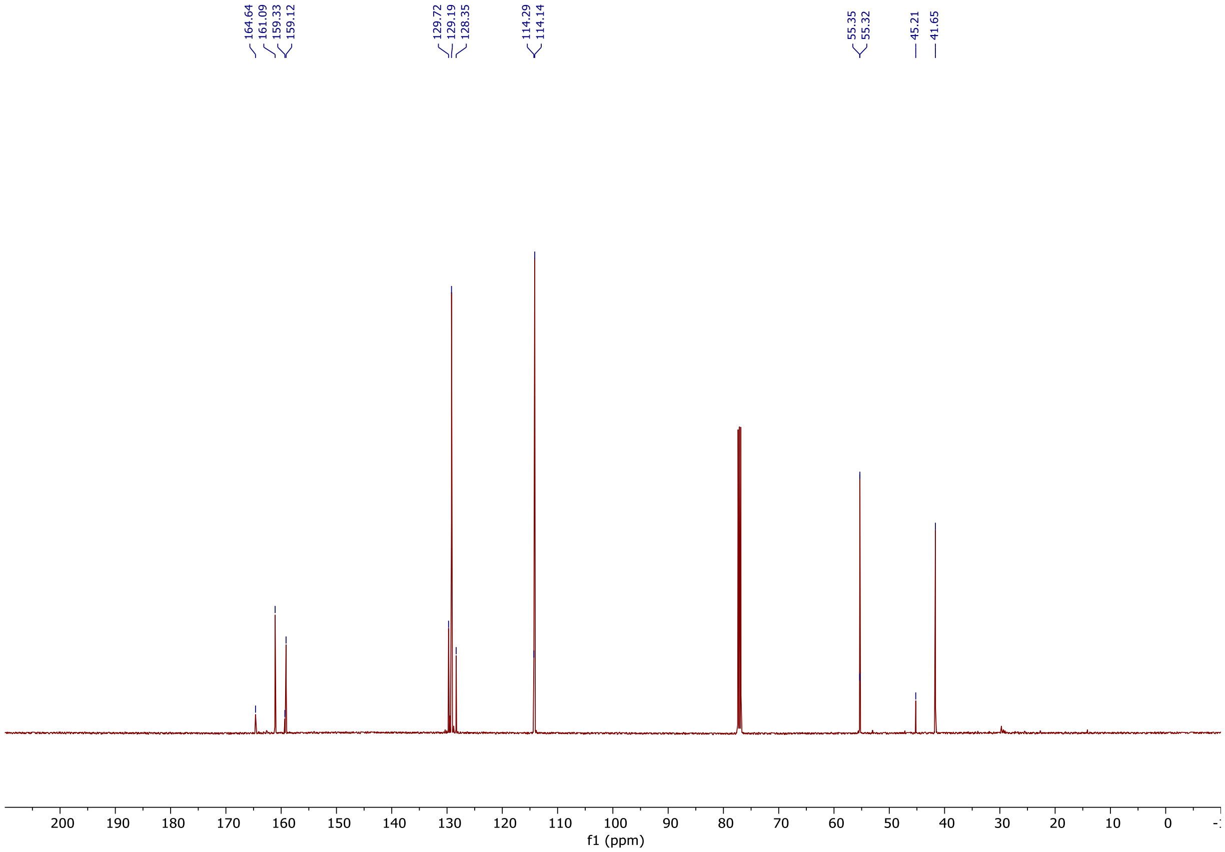
164.64
161.09
159.33
159.12
129.72
129.19
128.35
114.29
114.14
55.35
55.32
45.21
41.65
f1 (ppm)

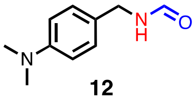
N
N
H
O

12

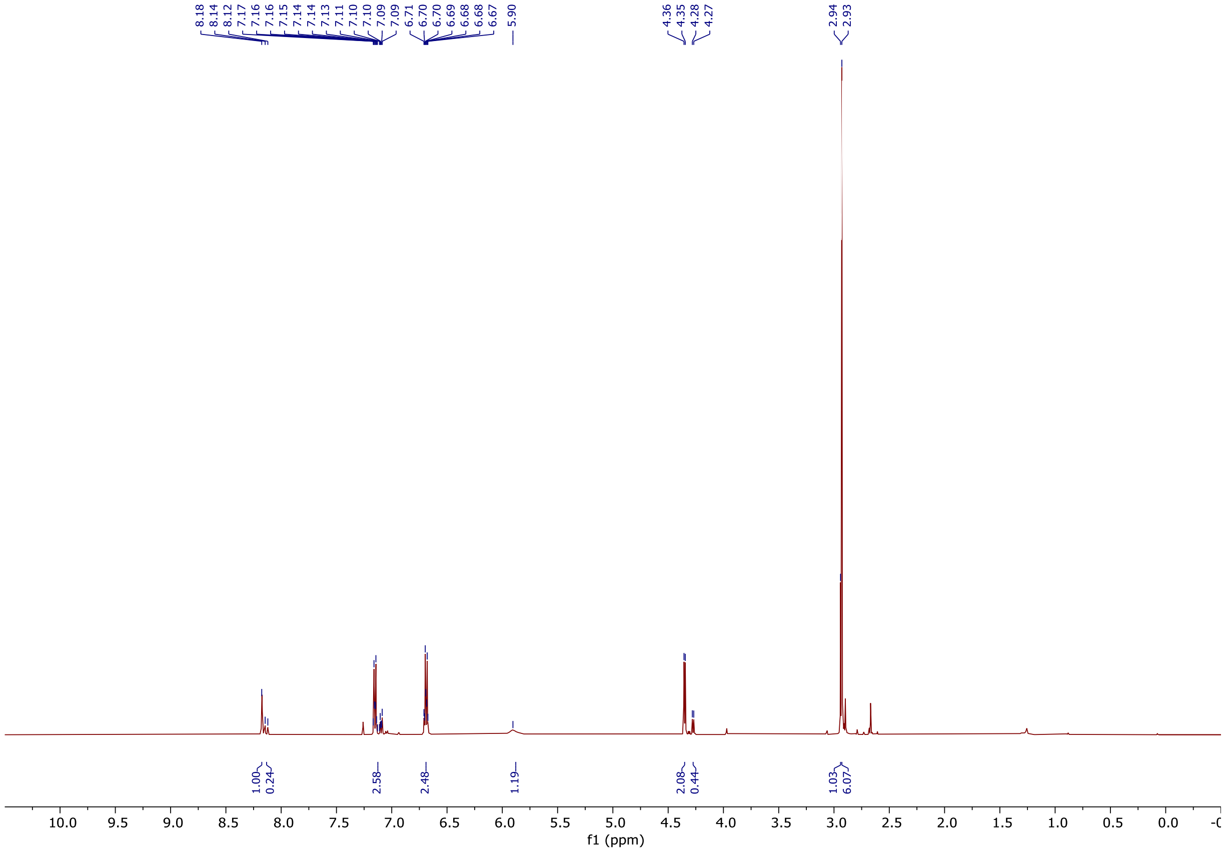
8.18
8.14
8.12
7.17
7.16
7.15
7.14
7.14
7.13
7.11
7.10
7.09
7.09
6.71
6.70
6.70
6.69
6.68
6.68
6.67
5.90
4.36
4.35
4.28
4.27
2.94
2.93
1.00
0.24
2.58
2.48
1.19
2.08
0.44
1.03
6.07
10.0 9.5 9.0 8.5 8.0 7.5 7.0 6.5 6.0 5.5 5.0 4.5 4.0 3.5 3.0 2.5 2.0 1.5 1.0 0.5 0.0
f1 (ppm)

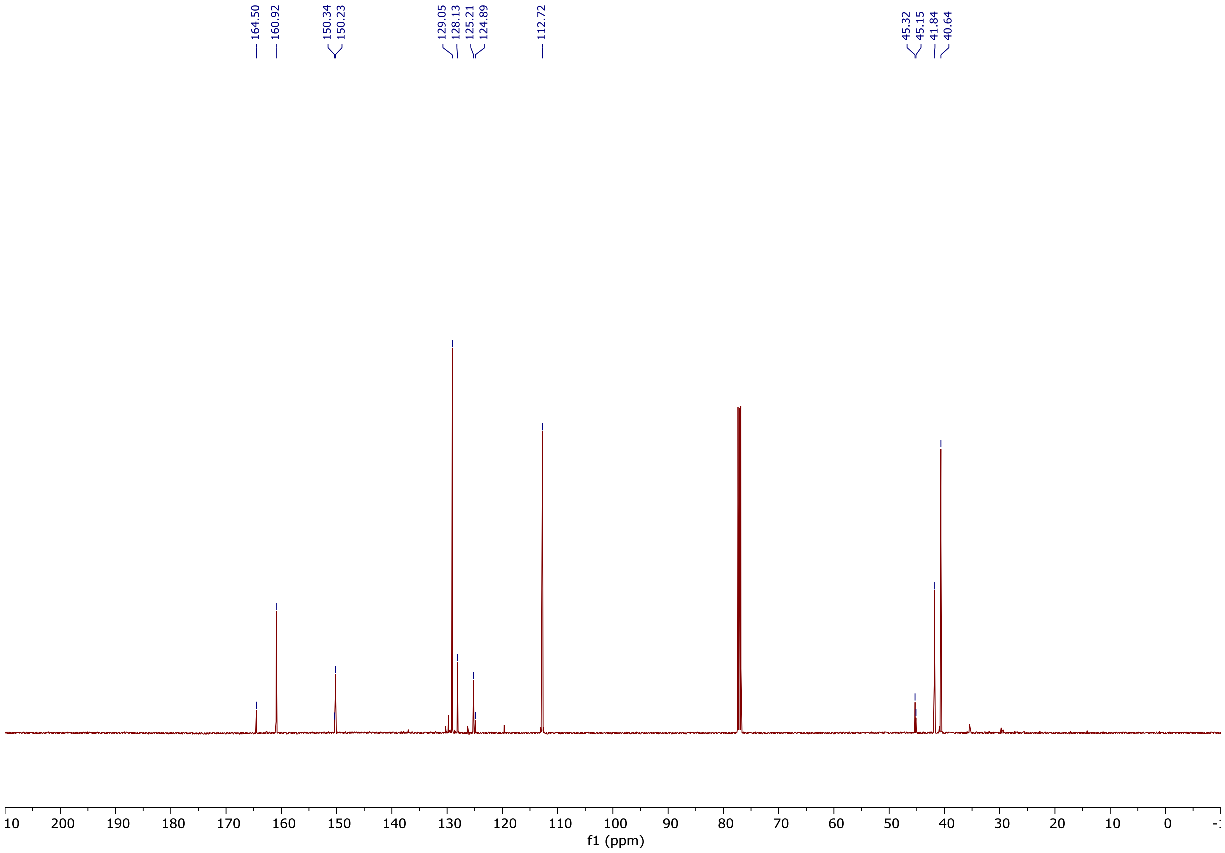
164.50
160.92
150.34
150.23
129.05
128.13
125.21
124.89
112.72
45.32
45.15
41.84
40.64
200 190 180 170 160 150 140 130 120 110 100 90 80 70 60 50 40 30 20 10 0
f1 (ppm)

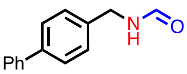
N
H
O
Ph

13

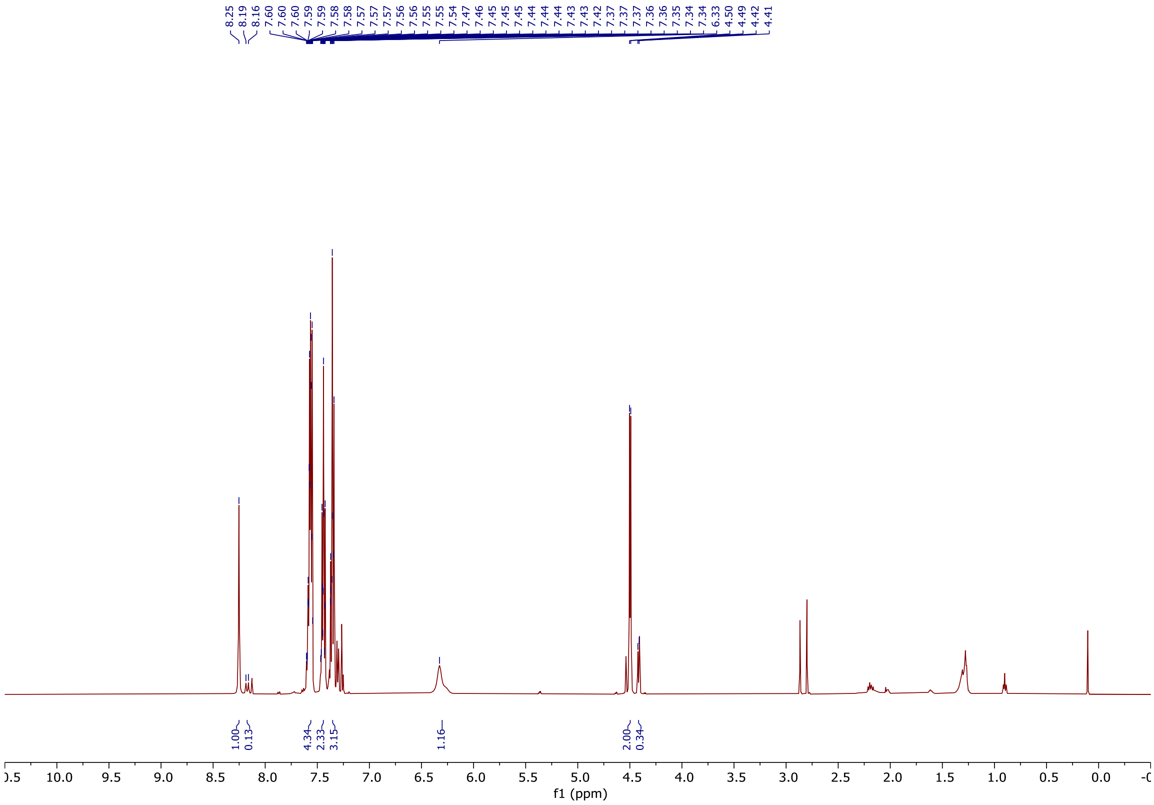
8.25
8.19
8.16
7.60
7.60
7.59
7.59
7.58
7.58
7.57
7.57
7.57
7.56
7.56
7.55
7.55
7.54
7.47
7.46
7.45
7.45
7.45
7.44
7.44
7.44
7.43
7.43
7.42
7.37
7.37
7.37
7.36
7.35
7.34
7.34
6.33
4.50
4.49
4.42
4.41
1.00
0.13
4.34
2.33
3.15
1.16
2.00
0.34
f1 (ppm)

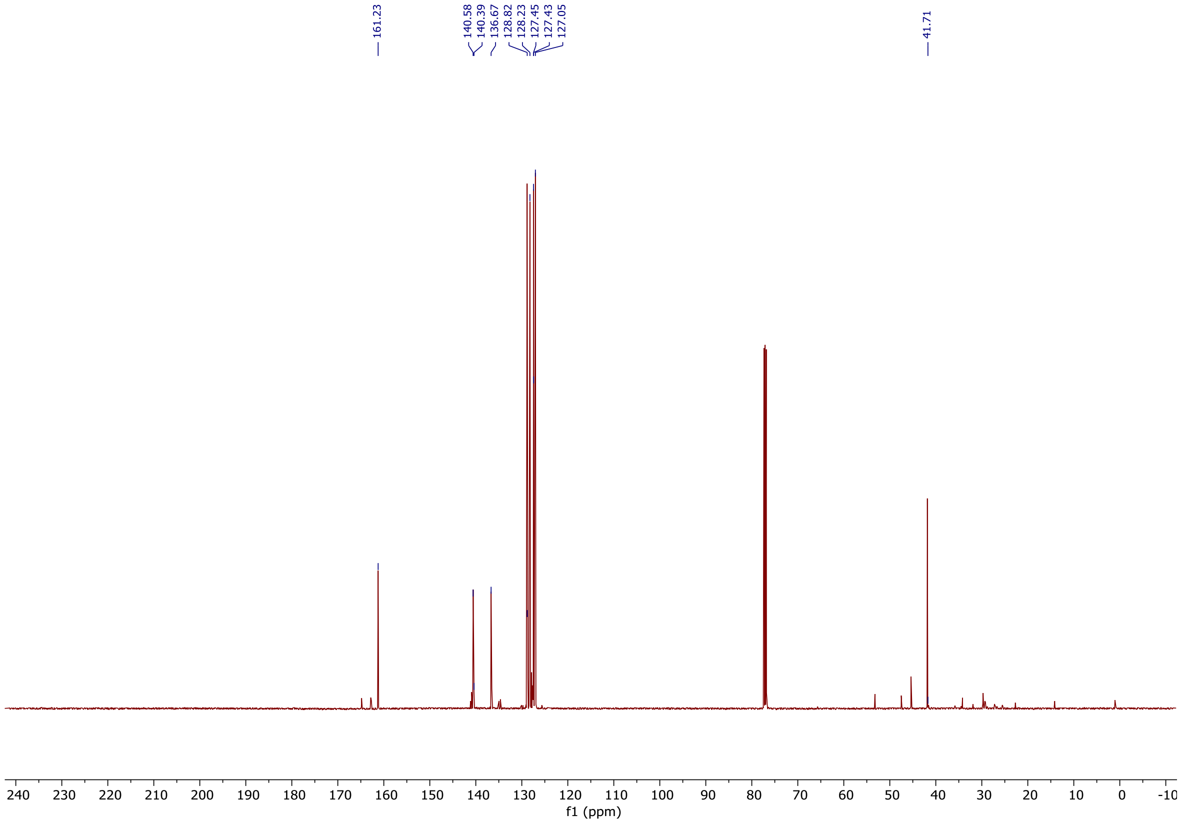
161.23
140.58
140.39
136.67
128.82
128.23
127.45
127.43
127.05
41.71
f1 (ppm)

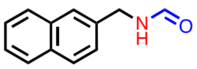
N
H
O

14

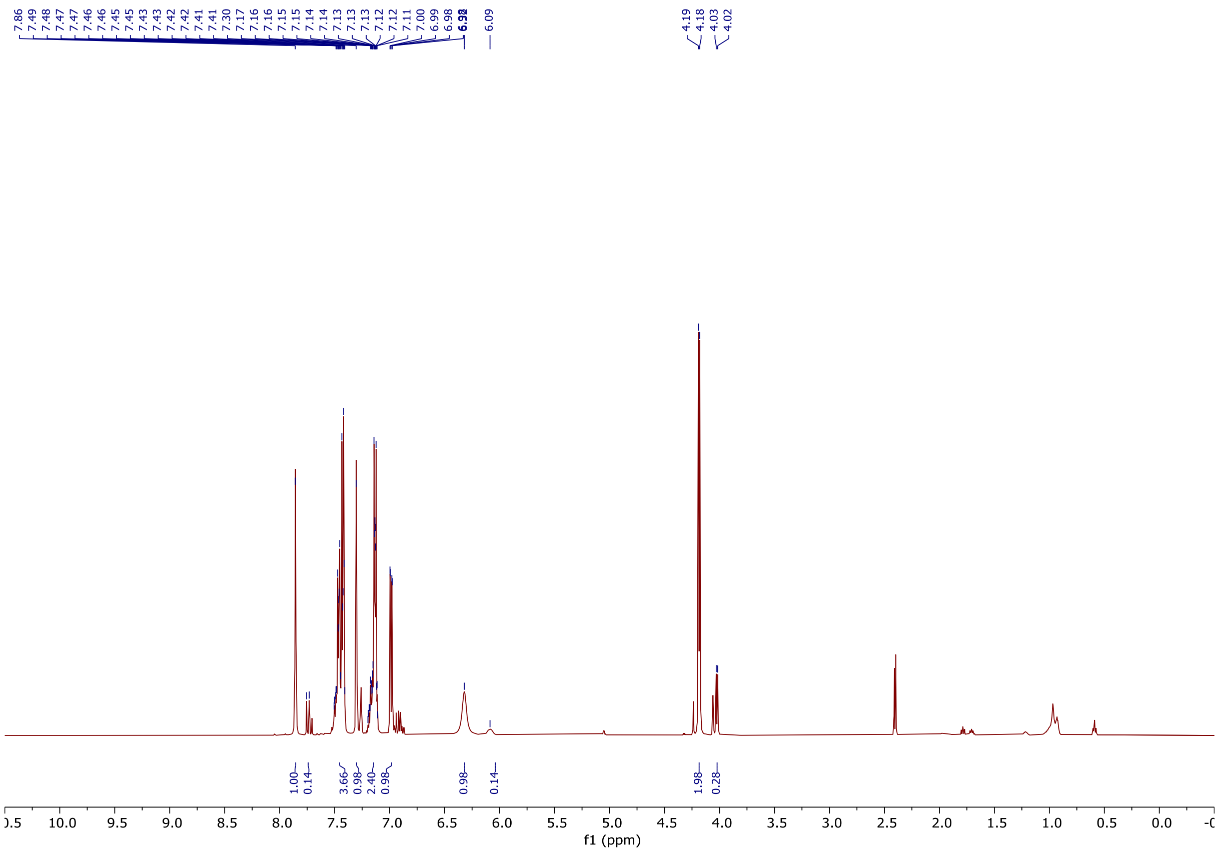
7.86
7.49
7.48
7.47
7.46
7.46
7.45
7.45
7.43
7.43
7.42
7.42
7.41
7.41
7.30
7.17
7.16
7.16
7.15
7.15
7.14
7.14
7.13
7.13
7.13
7.12
7.12
7.11
7.00
6.99
6.98
6.09
4.19
4.18
4.03
4.02
1.00
0.14
3.66
0.98
2.40
0.98
0.98
0.14
1.98
0.28
f1 (ppm)

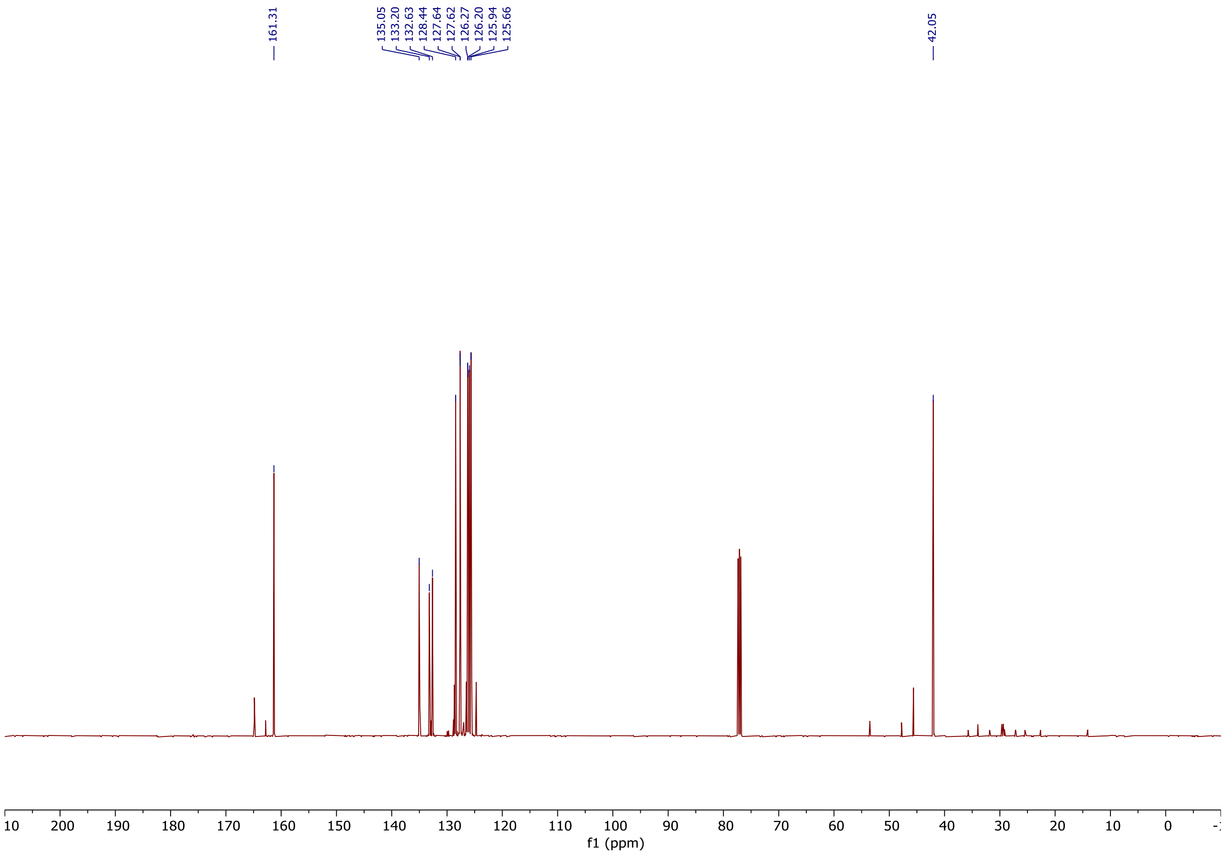
161.31
135.05
133.20
132.63
128.44
127.64
127.62
126.27
126.20
125.94
125.66
42.05
f1 (ppm)

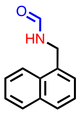
O
HN

15

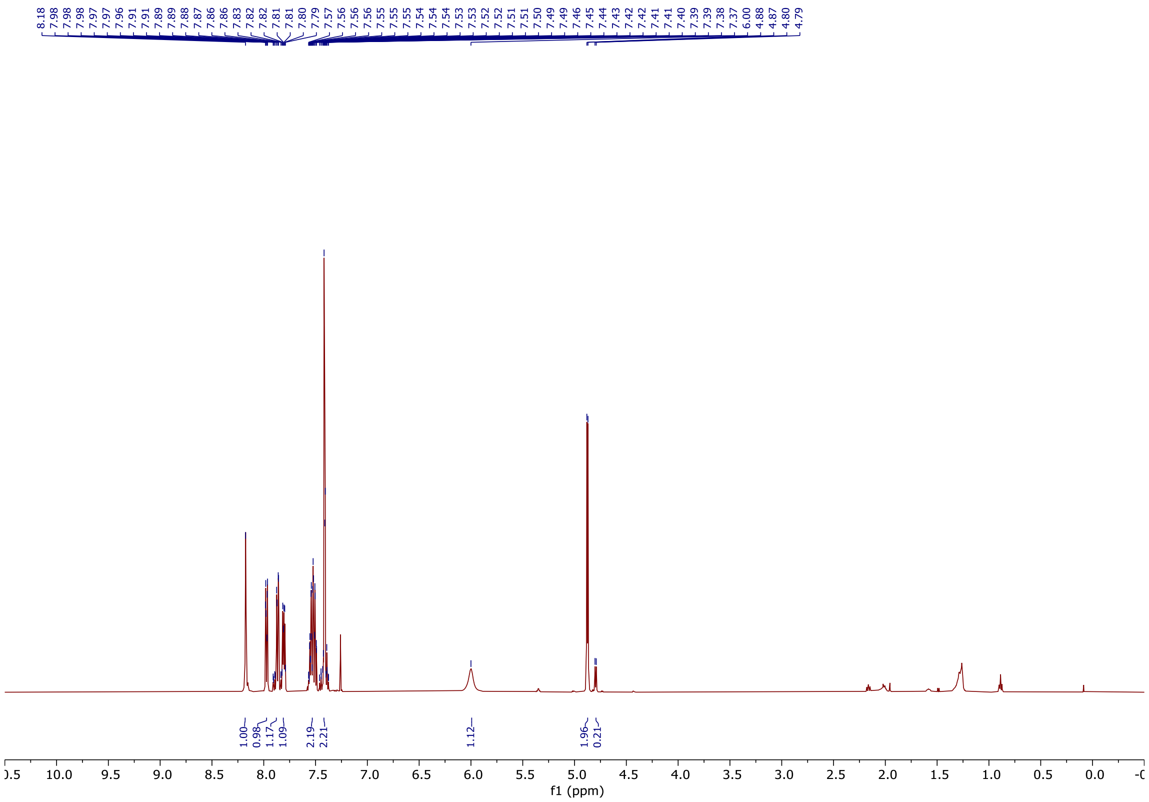
8.18
7.98
7.98
7.98
7.97
7.97
7.96
7.91
7.91
7.89
7.89
7.88
7.87
7.86
7.86
7.83
7.82
7.82
7.81
7.81
7.80
7.79
7.57
7.56
7.56
7.55
7.55
7.55
7.54
7.54
7.54
7.53
7.53
7.52
7.52
7.51
7.51
7.50
7.49
7.49
7.46
7.45
7.44
7.43
7.42
7.42
7.41
7.41
7.40
7.39
7.39
7.38
7.37
6.00
4.88
4.87
4.80
4.79
1.00
0.98
1.17
1.09
2.19
2.21
1.12
1.96
0.21
10.0
9.5
9.0
8.5
8.0
7.5
7.0
6.5
6.0
5.5
5.0
4.5
4.0
3.5
3.0
2.5
2.0
1.5
1.0
0.5
0.0
f1 (ppm)

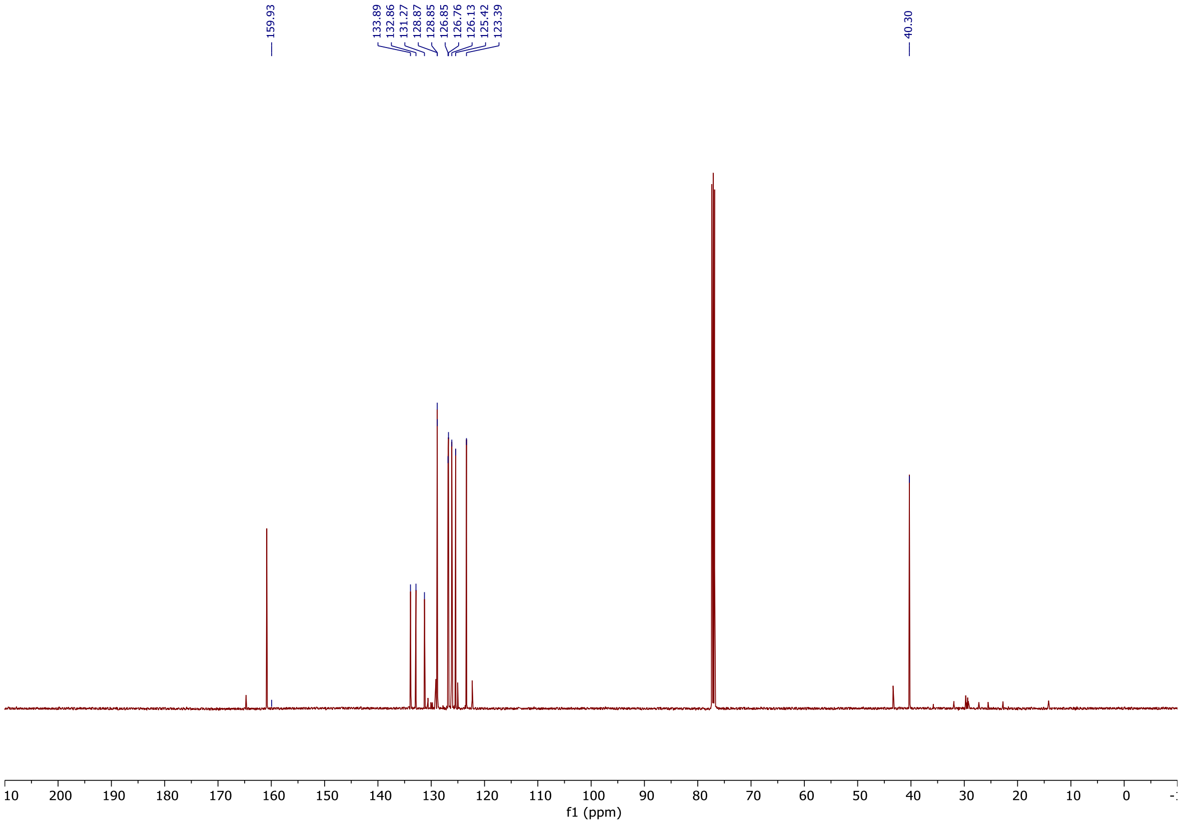
159.93
133.89
132.86
131.27
128.87
128.85
126.76
126.13
125.42
123.39
40.30
200
190
180
170
160
150
140
130
120
110
100
90
80
70
60
50
40
30
20
10
0
f1 (ppm)

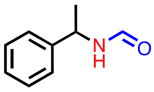

21

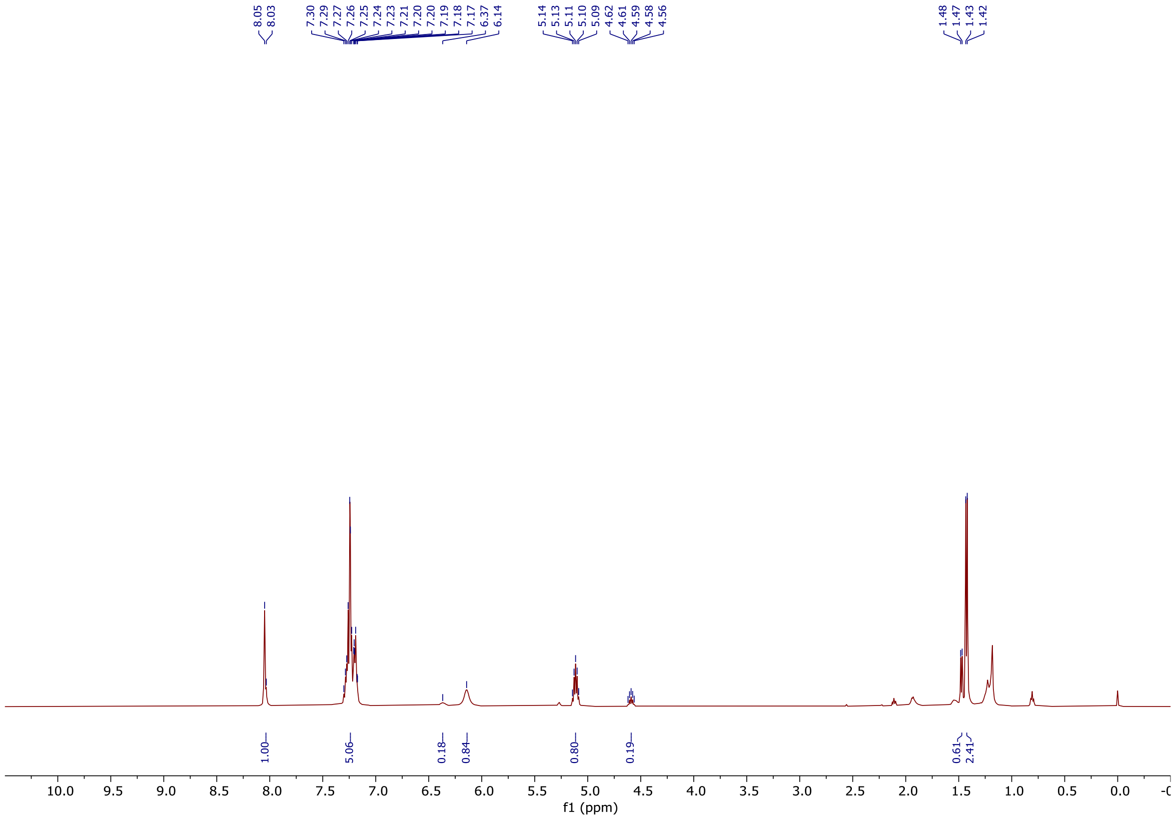
8.05
8.03
7.30
7.29
7.27
7.26
7.25
7.24
7.23
7.21
7.20
7.20
7.19
7.18
7.17
6.37
6.14
5.14
5.13
5.11
5.10
5.09
4.62
4.61
4.59
4.58
4.56
1.48
1.47
1.43
1.42
1.00
5.06
0.18
0.84
0.80
0.19
0.61
2.41
f1 (ppm)

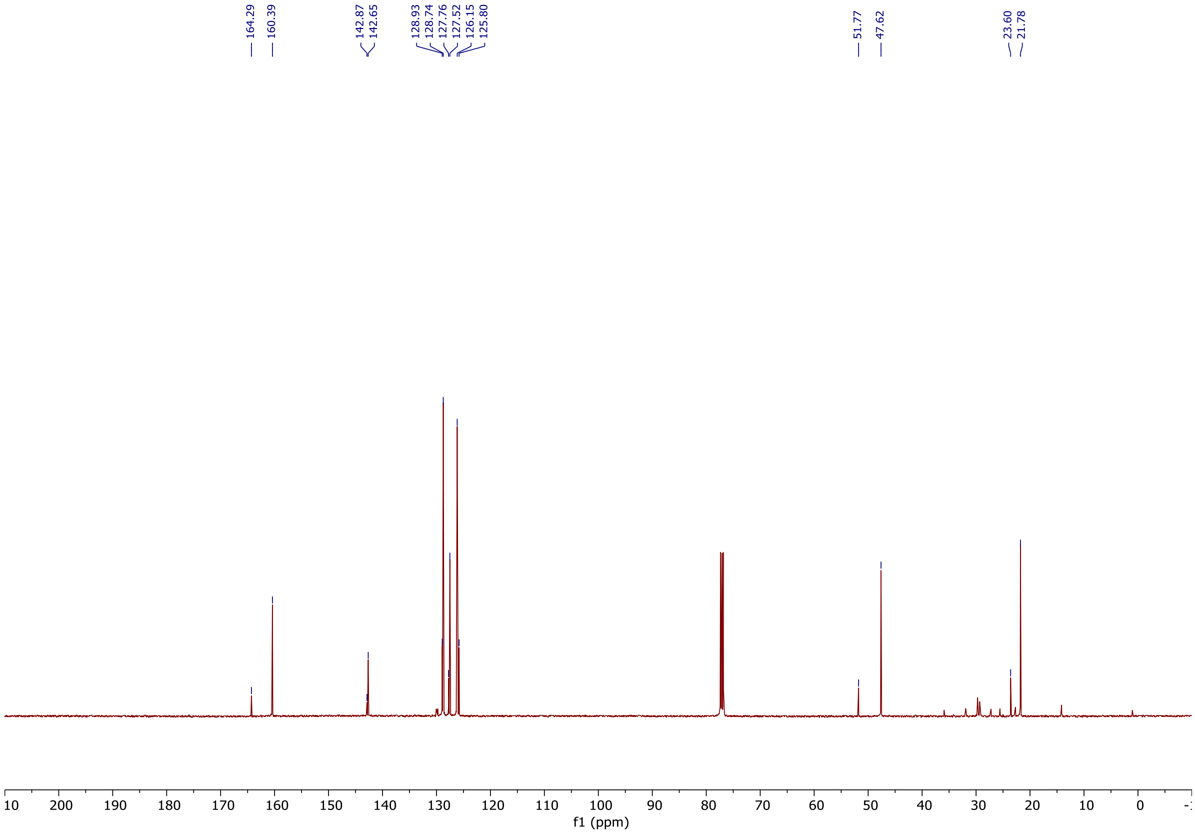
164.29
160.39
142.87
142.65
128.93
128.74
127.76
127.52
126.15
125.80
51.77
47.62
23.60
21.78
f1 (ppm)

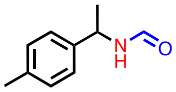
N
H
O

22

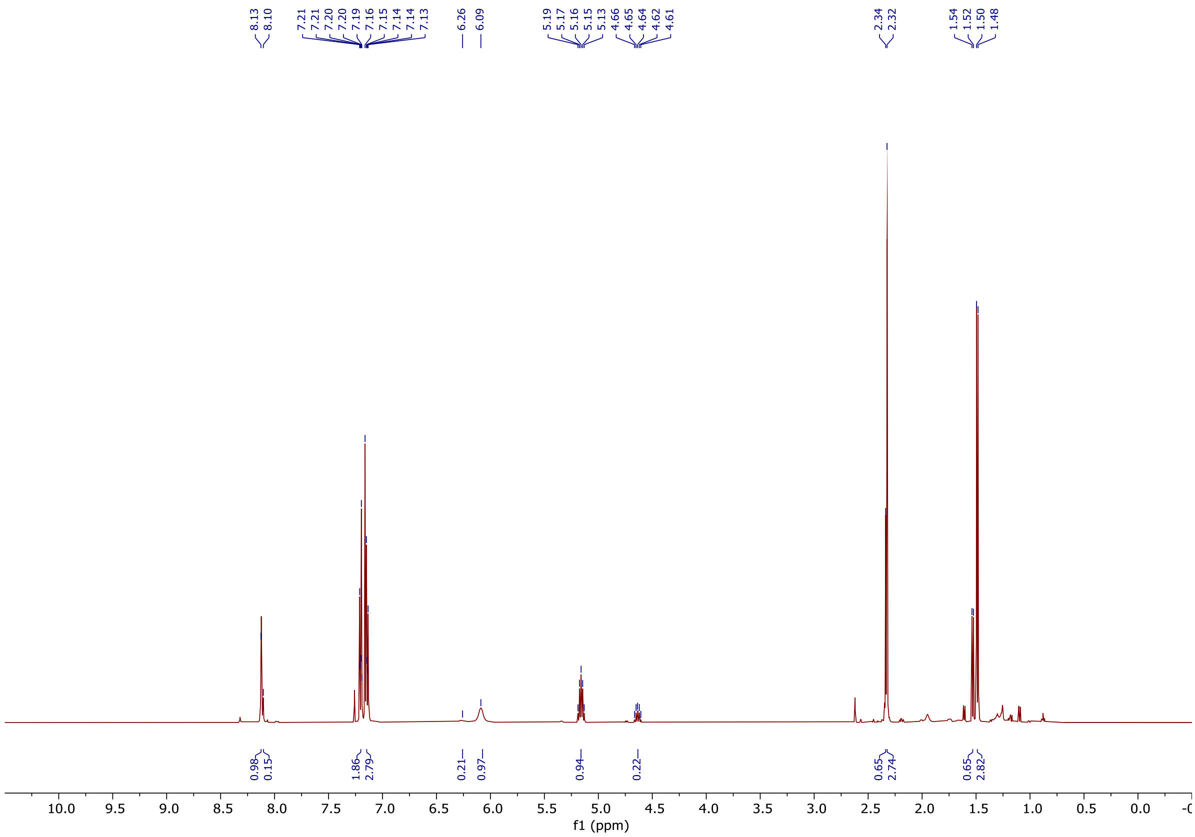
8.13
8.10
7.21
7.21
7.20
7.19
7.16
7.15
7.14
7.14
7.13
6.26
6.09
5.19
5.17
5.16
5.15
5.13
4.66
4.65
4.64
4.62
4.61
2.34
2.32
1.54
1.52
1.50
1.48
0.98
0.15
1.86
2.79
0.21
0.97
0.94
0.22
0.65
2.74
0.65
2.82
f1 (ppm)

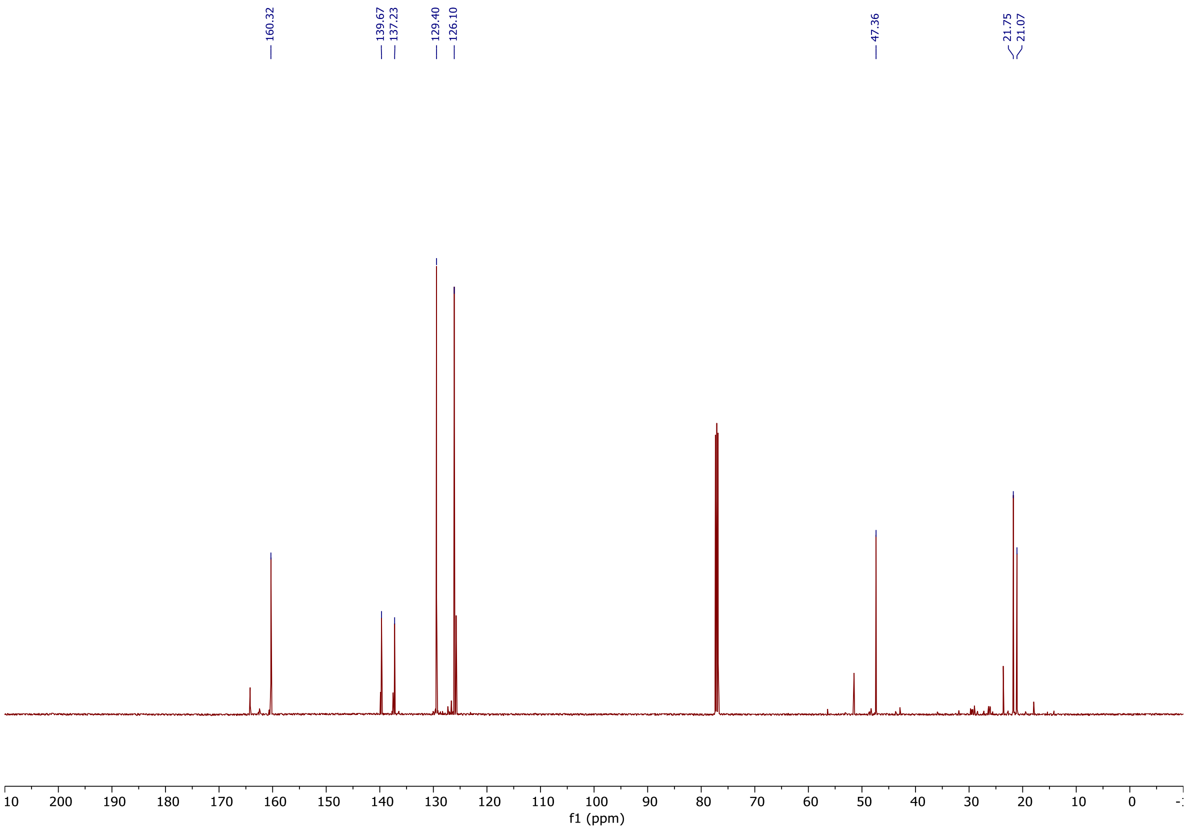
160.32
139.67
137.23
129.40
126.10
47.36
21.75
21.07
f1 (ppm)

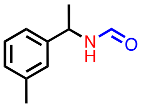
N
H
O

23

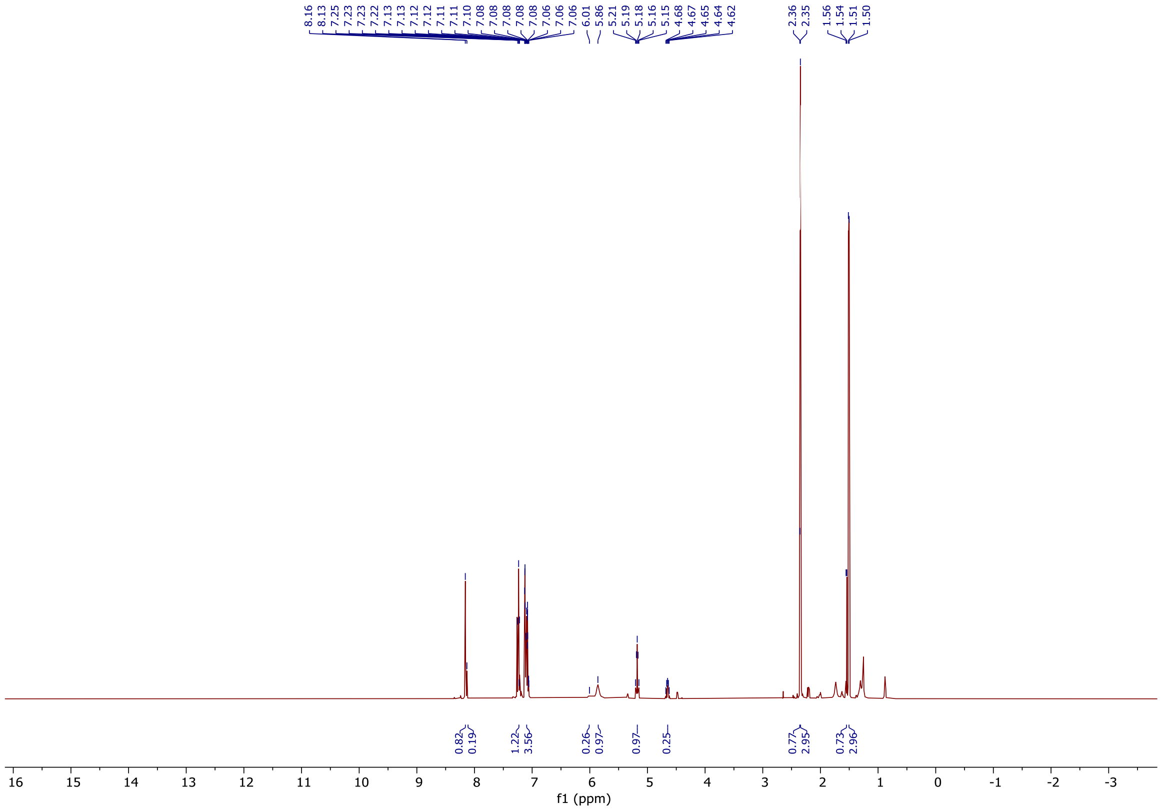
8.16
8.13
7.25
7.23
7.23
7.22
7.13
7.13
7.12
7.12
7.11
7.10
7.08
7.08
7.08
7.08
7.08
7.06
7.06
7.06
6.01
5.86
5.21
5.19
5.18
5.16
5.15
4.68
4.67
4.65
4.64
4.62
2.36
2.35
1.56
1.54
1.51
1.50
0.82
0.19
1.22
3.56
0.26
0.97
0.97
0.25
0.77
2.95
0.73
2.96
16 15 14 13 12 11 10 9 8 7 6 5 4 3 2 1 0 -1 -2 -3
f1 (ppm)

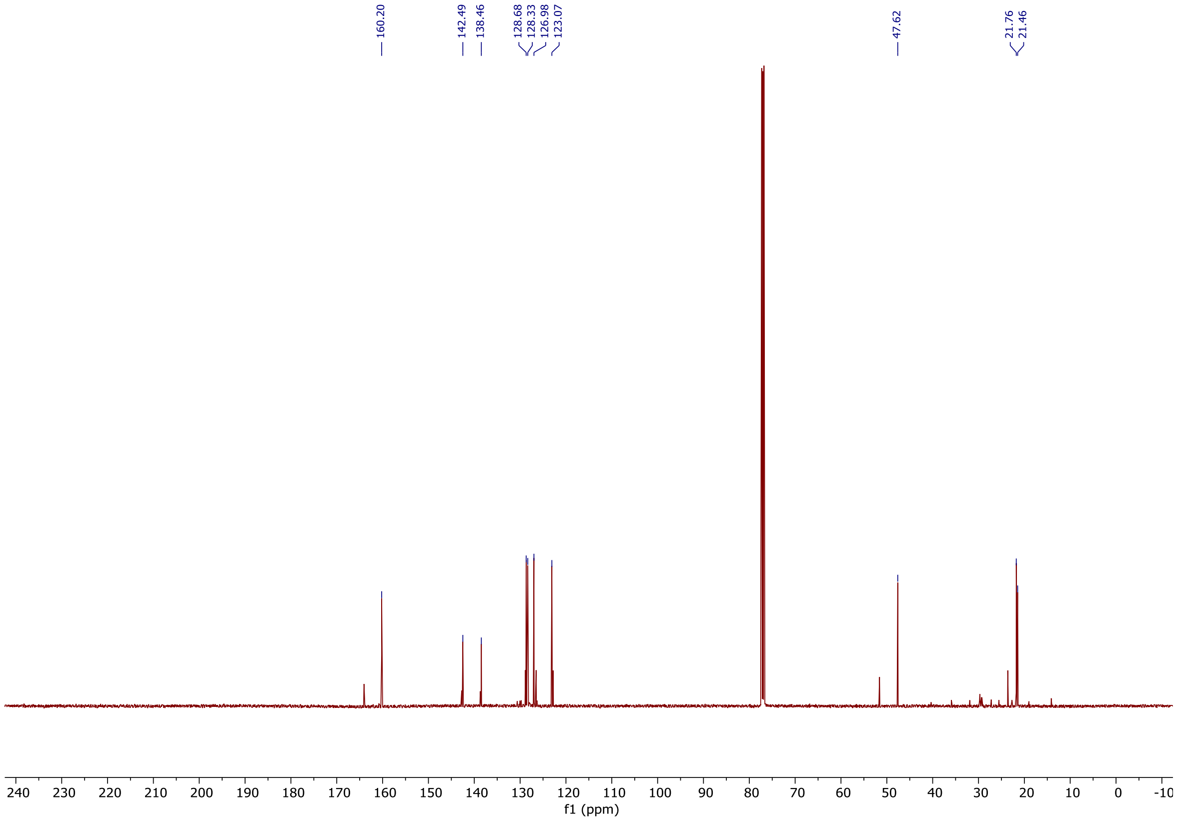
160.20
142.49
138.46
128.68
128.33
126.98
123.07
47.62
21.76
21.46
240 230 220 210 200 190 180 170 160 150 140 130 120 110 100 90 80 70 60 50 40 30 20 10 0 -10
f1 (ppm)

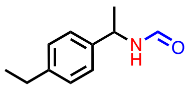
N
H
O

24

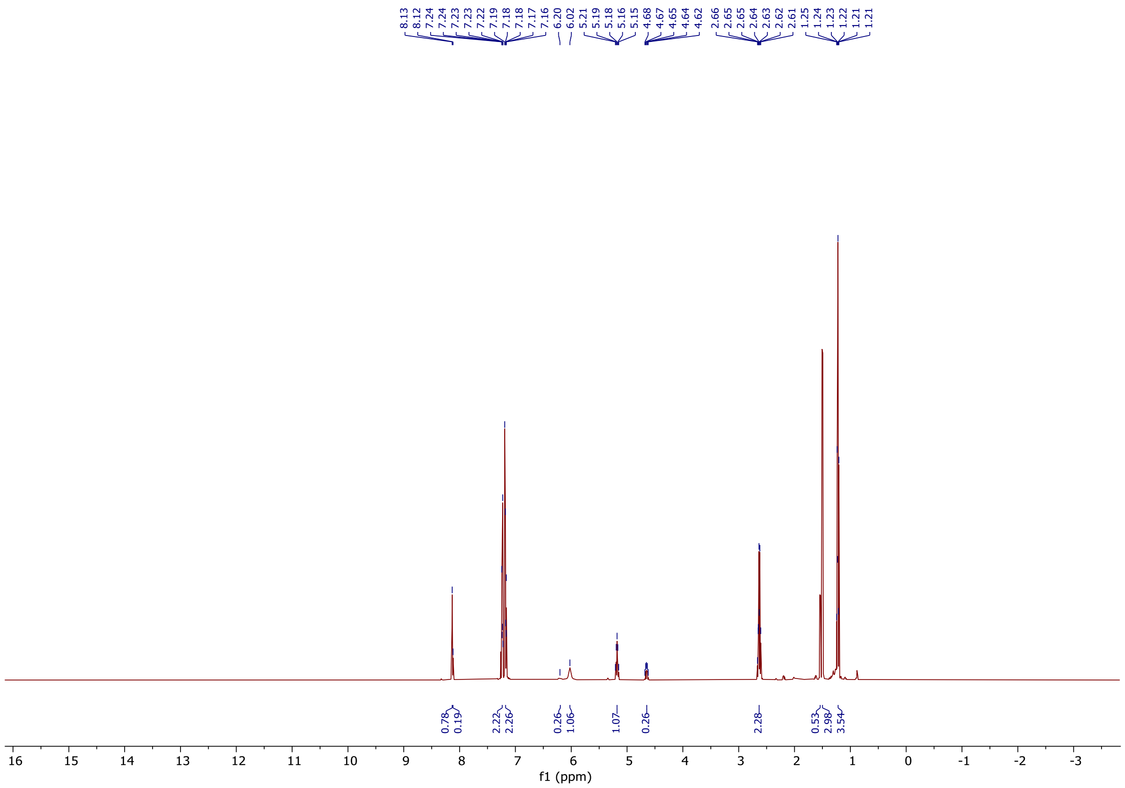
8.13 8.12 7.24 7.24 7.23 7.23 7.22 7.19 7.18 7.18 7.17 7.16 6.20 6.02 5.21 5.19 5.18 5.16 5.15 4.68 4.67 4.65 4.64 4.62 2.66 2.65 2.65 2.64 2.63 2.62 2.61 1.25 1.24 1.23 1.22 1.21 1.21
0.78 0.19 2.22 2.26 0.26 1.06 1.07 0.26 2.28 0.53 2.98 3.54
16 15 14 13 12 11 10 9 8 7 6 5 4 3 2 1 0 -1 -2 -3
f1 (ppm)

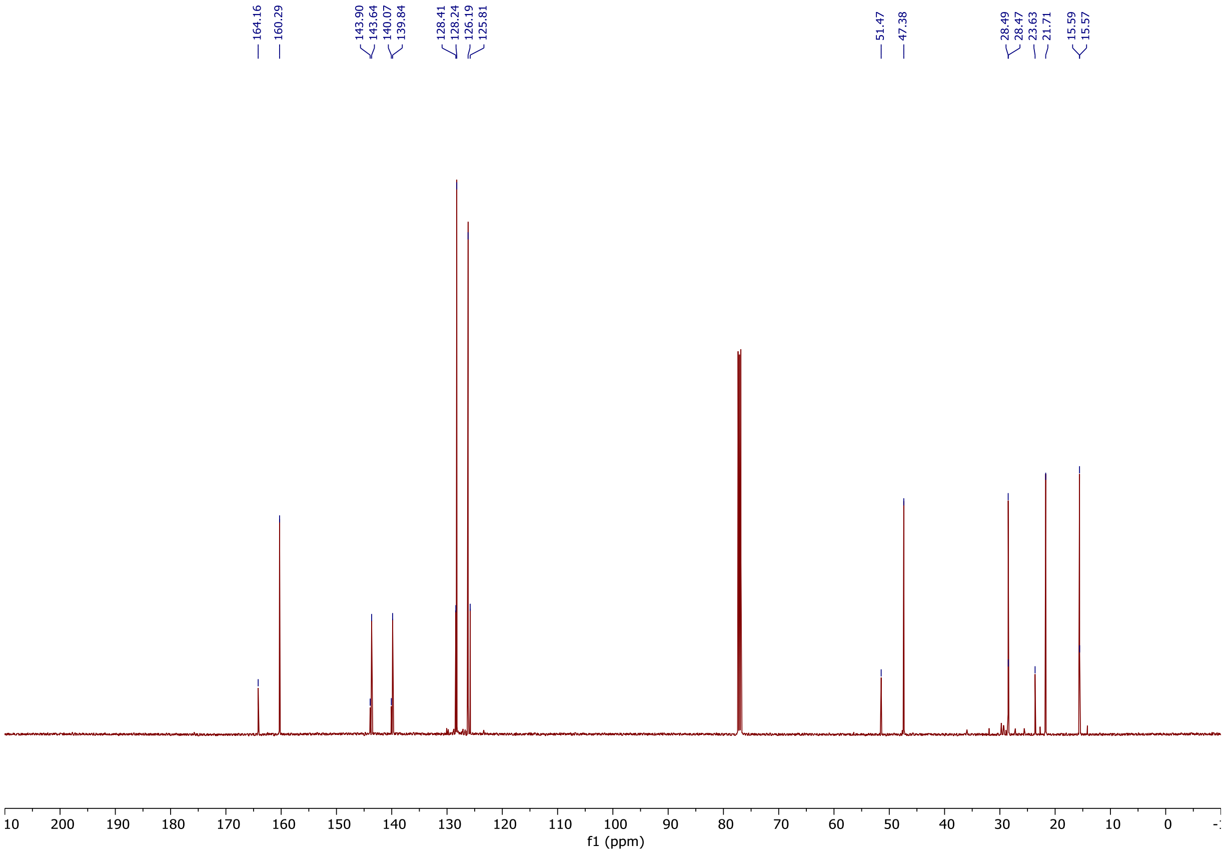
164.16 160.29 143.90 143.64 140.07 139.84 128.41 128.24 126.19 125.81 51.47 47.38 28.49 28.47 23.63 21.71 15.59 15.57
10 200 190 180 170 160 150 140 130 120 110 100 90 80 70 60 50 40 30 20 10 0
f1 (ppm)

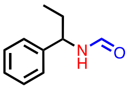

25

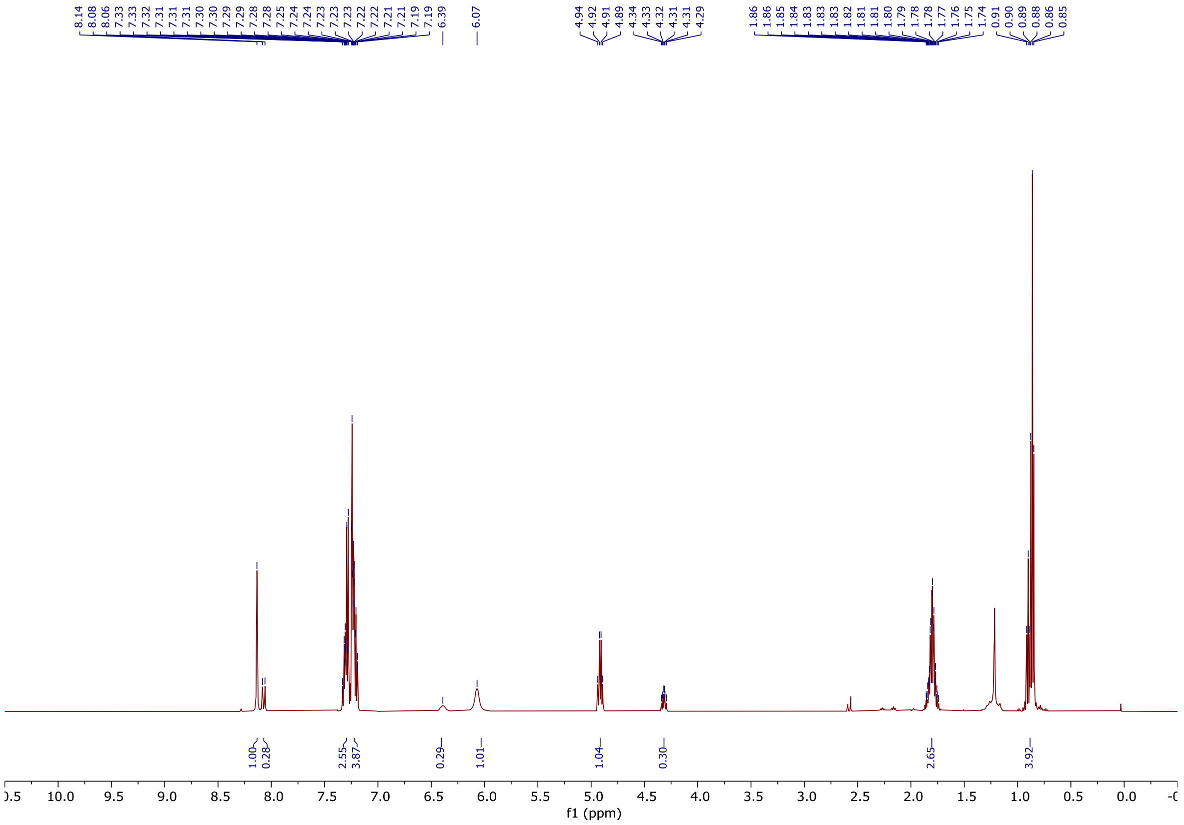

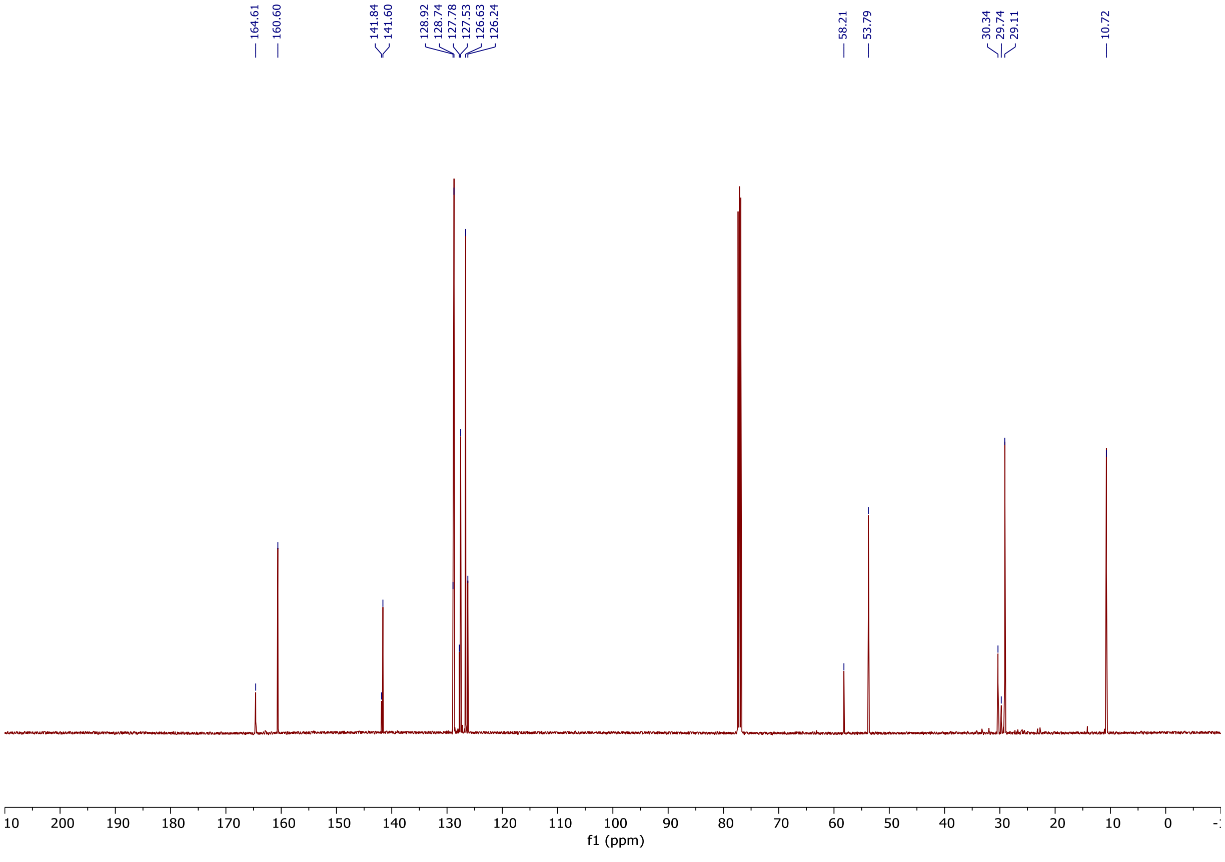

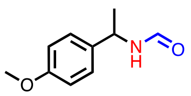

26

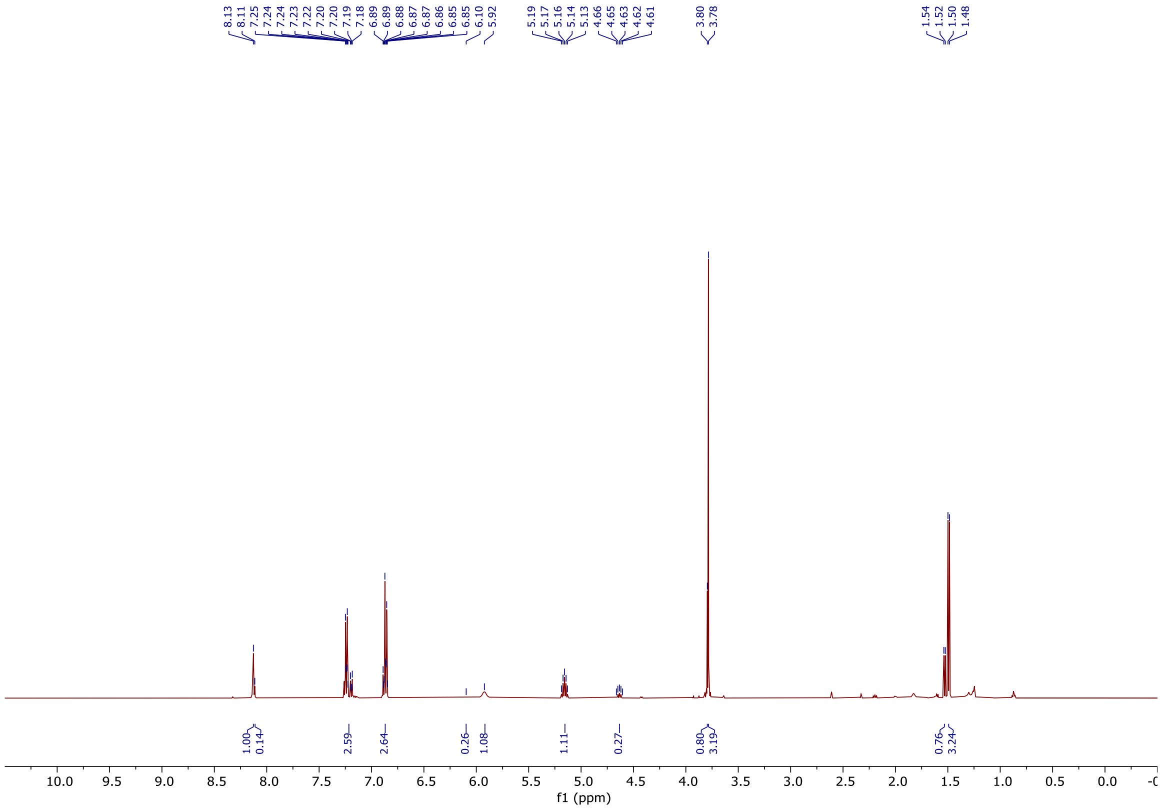
8.13 8.11 7.25 7.24 7.24 7.23 7.22 7.22 7.20 7.20 7.19 7.18 6.89 6.89 6.88 6.87 6.87 6.86 6.85 6.85 6.10 5.92
5.19 5.17 5.16 5.14 5.13 4.66 4.65 4.63 4.62 4.61
3.80 3.78
1.54 1.52 1.50 1.48
1.00 0.14 2.59 2.64 0.26 1.08 1.11 0.27 0.80 3.19 0.76 3.24
f1 (ppm)

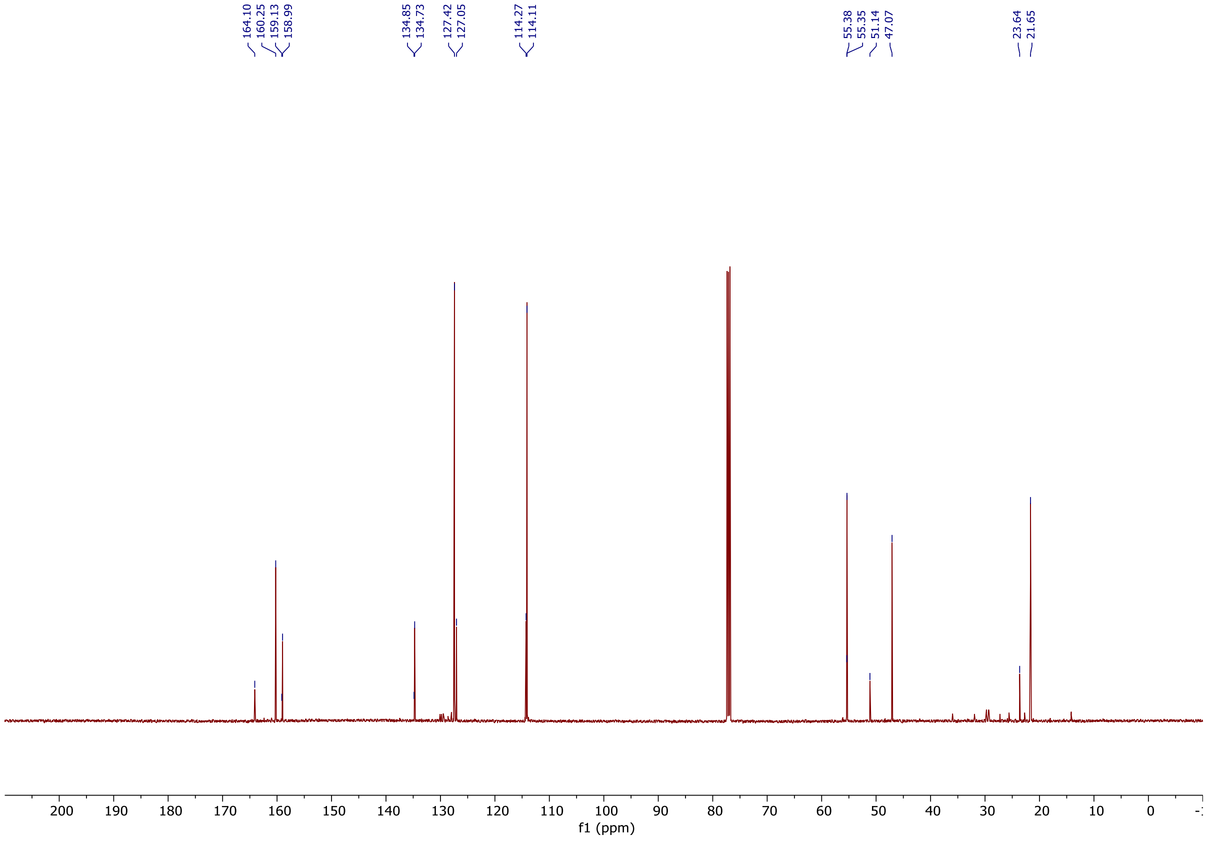
164.10 160.25 159.13 158.99
134.85 134.73 127.42 127.05
114.27 114.11
55.38 55.35 51.14 47.07
23.64 21.65
f1 (ppm)

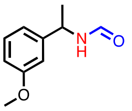
N
H
O
O

27

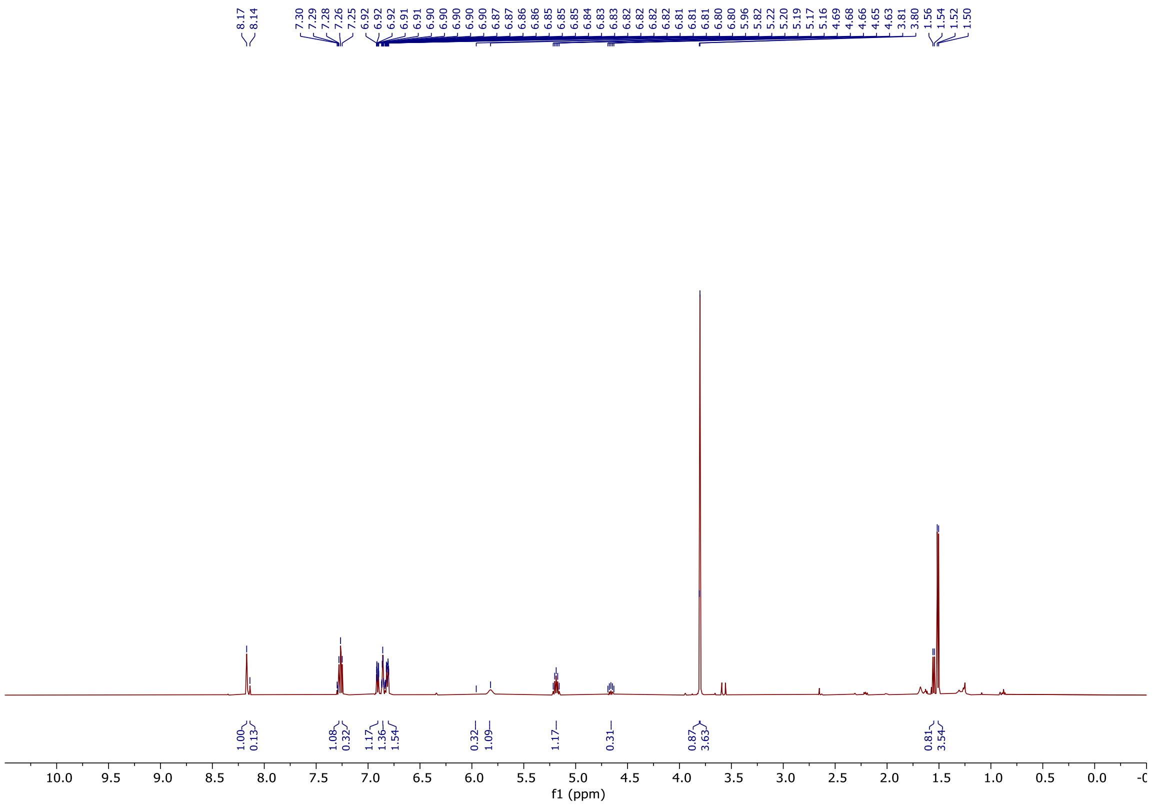
8.17
8.14
7.30
7.29
7.28
7.26
7.25
6.92
6.92
6.92
6.91
6.91
6.90
6.90
6.90
6.90
6.90
6.87
6.86
6.86
6.85
6.85
6.85
6.84
6.83
6.83
6.82
6.82
6.82
6.82
6.81
6.81
6.81
6.80
6.80
5.96
5.82
5.22
5.20
5.19
5.17
5.16
4.69
4.68
4.66
4.65
4.63
3.81
3.80
1.56
1.54
1.52
1.50
1.00
0.13
1.08
0.32
1.17
1.36
1.54
0.32
1.09
1.17
0.31
0.87
3.63
0.81
3.54
10.0
9.5
9.0
8.5
8.0
7.5
7.0
6.5
6.0
5.5
5.0
4.5
4.0
3.5
3.0
2.5
2.0
1.5
1.0
0.5
0.0
f1 (ppm)

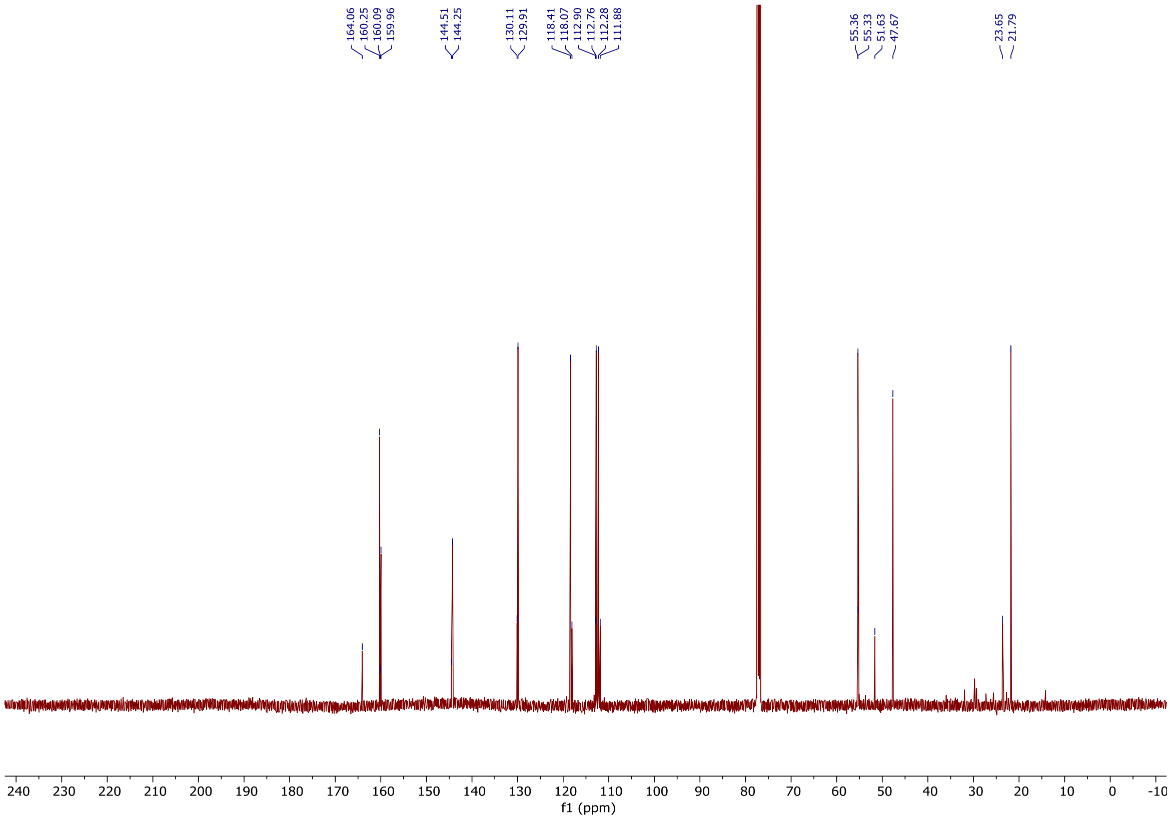
164.06
160.25
160.09
159.96
144.51
144.25
130.11
129.91
118.41
118.07
112.90
112.76
112.28
111.88
55.36
55.33
51.63
47.67
23.65
21.79
240
230
220
210
200
190
180
170
160
150
140
130
120
110
100
90
80
70
60
50
40
30
20
10
0
-10
f1 (ppm)

5. References

1. Miller, J. T.; Kropf, A. J.; Zha, Y.; Regalbuto, J. R.; Delannoy, L.; Louis, C.; Bus, E.; van Bokhoven, J. A., The effect of gold particle size on AuAu bond length and reactivity toward oxygen in supported catalysts. *J. Catal*. **2006**, 240 (2), 222-234.

2. Yin, J.; Zhang, J.; Cai, C.; Deng, G. J.; Gong, H., Catalyst-Free Transamidation of Aromatic Amines with Formamide Derivatives and Tertiary Amides with Aliphatic Amines. *Org. Lett*. **2019**, 21, 387-392.

3. Kang, B.; Hong, S. H., Hydrogen Acceptor- and Base-FreeN-Formylation of Nitriles and Amines using Methanol as C1Source. *Adv. Syn. Catal*. **2015**, 357, 834-840.

4. Ortega, N.; Richter, C.; Glorius, F., N-formylation of amines by methanol activation. *Org. Lett.* **2013**, 15, 1776-1779.

5. Chong, C. C.; Kinjo, R., Hydrophosphination of CO2 and subsequent formate transfer in the 1,3,2-diazaphospholene-catalyzed N-formylation of amines. *Angew. Chem. Int. Ed*. **2015**, 54, 12116-20.

6. Neochoritis, C. G.; Stotani, S.; Mishra, B.; Domling, A., Efficient isocyanide-less isocyanide-based multicomponent reactions. *Org. Lett*. **2015**, 17, 2002-5.

7. Chen, J.; Jia, J.; Guo, Z.; Zhang, J.; Xie, M., NH4I-promoted N-acylation of amines via the transamidation of DMF and DMA under metal-free conditions. *Tetra. Lett.* **2019**, 60, 1426-1429.

8. Kobayashi, K.; Matsumoto, N.; Matsumoto, K., Synthesis of 3,4-Dihydroisoquinolines by Cyclization of 1-Bromo-2-(2-isocyanoalkyl)benzenes with Butyllithium. *Heterocycles*, **2013**, 87, 389-397.
